# Predictors and correlates of adherence to combination antiretroviral therapy (ART) for chronic HIV infection: a meta-analysis

**DOI:** 10.1186/s12916-014-0142-1

**Published:** 2014-08-21

**Authors:** Nienke Langebeek, Elizabeth H Gisolf, Peter Reiss, Sigrid C Vervoort, Thóra B Hafsteinsdóttir, Clemens Richter, Mirjam AG Sprangers, Pythia T Nieuwkerk

**Affiliations:** Department of Internal Medicine, Rijnstate Hospital, Wagnerlaan 55, Arnhem, 6815 AD Netherlands; Department of Medical Psychology, Academic Medical Center, Meibergdreef 9, Amsterdam, 1105 AZ Netherlands; Division of Infectious Diseases, and Department of Global Health, Amsterdam Institute for Global Health and Development, Academic Medical Center, Meibergdreef 9, Amsterdam, 1105 AZ Netherlands; Stichting HIV Monitoring, Meibergdreef 9, Amsterdam, 1105 AZ Netherlands; Department of Infectious Diseases, University Medical Center, Heidelberglaan 100, Utrecht, 3584 CX Netherlands; Department of Rehabilitation, Nursing Science and Sports medicine, University Medical Center, Heidelberglaan 100, Utrecht, 3584 CX Netherlands; Department of Medical Psychology (J3-219-1), Academic Medical Center, Amsterdam, 1100 DE Netherlands

**Keywords:** Adherence, Compliance, HIV infection, Antiretroviral therapy, Meta-analysis

## Abstract

**Background:**

Adherence to combination antiretroviral therapy (ART) is a key predictor of the success of human immunodeficiency virus (HIV) treatment, and is potentially amenable to intervention. Insight into predictors or correlates of non-adherence to ART may help guide targets for the development of adherence-enhancing interventions. Our objective was to review evidence on predictors/correlates of adherence to ART, and to aggregate findings into quantitative estimates of their impact on adherence.

**Methods:**

We searched PubMed for original English-language papers, published between 1996 and June 2014, and the reference lists of all relevant articles found. Studies reporting on predictors/correlates of adherence of adults prescribed ART for chronic HIV infection were included without restriction to adherence assessment method, study design or geographical location. Two researchers independently extracted the data from the same papers. Random effects models with inverse variance weights were used to aggregate findings into pooled effects estimates with 95% confidence intervals. The standardized mean difference (SMD) was used as the common effect size. The impact of study design features (adherence assessment method, study design, and the United Nations Human Development Index (HDI) of the country in which the study was set) was investigated using categorical mixed effects meta-regression.

**Results:**

In total, 207 studies were included. The following predictors/correlates were most strongly associated with adherence: adherence self-efficacy (SMD = 0.603, *P* = 0.001), current substance use (SMD = -0.395, *P* = 0.001), concerns about ART (SMD = -0.388, *P* = 0.001), beliefs about the necessity/utility of ART (SMD = 0.357, *P* = 0.001), trust/satisfaction with the HIV care provider (SMD = 0.377, *P* = 0.001), depressive symptoms (SMD = -0.305, *P* = 0.001), stigma about HIV (SMD = -0.282, *P* = 0.001), and social support (SMD = 0.237, *P* = 0.001). Smaller but significant associations were observed for the following being prescribed a protease inhibitor-containing regimen (SMD = -0.196, *P* = 0.001), daily dosing frequency (SMD = -0.193, *P* = 0.001), financial constraints (SMD -0.187, *P* = 0.001) and pill burden (SMD = -0.124, *P* = 0.001). Higher trust/satisfaction with the HIV care provider, a lower daily dosing frequency, and fewer depressive symptoms were more strongly related with higher adherence in low and medium HDI countries than in high HDI countries.

**Conclusions:**

These findings suggest that adherence-enhancing interventions should particularly target psychological factors such as self-efficacy and concerns/beliefs about the efficacy and safety of ART. Moreover, these findings suggest that simplification of regimens might have smaller but significant effects.

**Electronic supplementary material:**

The online version of this article (doi:10.1186/s12916-014-0142-1) contains supplementary material, which is available to authorized users.

## Background

Adherence to combination antiretroviral therapy (ART) is a key predictor of antiretroviral treatment success, and is potentially amenable to intervention [1]. Sufficiently high levels of adherence to ART are necessary to achieve and sustain viral suppression and to prevent disease progression and death [2], yet, many patients infected with human immunodeficiency virus (HIV)do not succeed in achieving or maintaining adequate levels of adherence to ART [3].

Insight into predictors or correlates of non-adherence to ART offers the potential to identify patients at risk for low levels of adherence. This would enable healthcare providers to target those patients in most need, and to tailor their care appropriately. Moreover, knowledge of predictors/correlates of non-adherence to ART may help guide targets for the development of interventions to enhance or maintain adherence to ART.

Medication adherence is considered to be a complex behavior that is influenced by a wide range of factors, which have previously been categorized into sociodemographic, condition-related, treatment-related, patient-related, and interpersonal factors [4],[5]. In recent years, a number of systematic reviews and meta-analyses have investigated predictors/correlates of adherence by patients prescribed ART for chronic HIV infection. One systematic review provided a comprehensive assessment of predictors/correlates of adherence to ART, but did not aggregate findings into quantitative estimates of their effect on adherence [5]. A number of other reviews did aggregate findings into quantitative estimates, but focused only on patient-reported barriers and facilitators [1], sociodemographic factors [3], clinical, comorbid, and treatment-related factors [6] and depression [7], or investigated a particular patient population, such as drug users [8].

The objective of the present study was to carry out a comprehensive review into the current research evidence on sociodemographic, treatment-related, condition-related, patient-related, and interpersonal predictors and correlates of adherence to ART, and to aggregate the findings into quantitative estimates of their impact on adherence. Thereby, we aimed to assess the relative importance of each predictor/correlate of adherence. Studies on adherence to ART have been conducted in a variety of countries and settings, and have used a variety of research designs and adherence measurement methods. For these reasons, another aim was to assess the impact of such study design features on predictors/correlates of adherence.

## Methods

Our meta-analysis was conducted in accordance with Preferred Reporting Items for Systematic Reviews and Meta-Analyses (PRISMA) statement guidelines [9]. Two of the authors (NL and PN) searched PubMed for papers published from August 1996 to June 2014 using the following strategy: Search: (((((“adult”[MeSH Terms] AND hasabstract[text] AND ( “1996/01/01”[PDat] : “2014/06/10”[PDat] ))) AND ((“patient compliance”[MeSH Terms] OR “medication adherence”[MeSH Terms] AND hiv) AND hasabstract[text] AND ( “1996/01/01”[PDat] : “2014/06/10”[PDat] ))) AND hasabstract[text] AND ( “1996/01/01”[PDat] : “2014/06/10”[PDat] ))) NOT children[MeSH Terms] Filters: Abstract available, From 1996/01/01 to 2014/06/10. Additionally, the reference lists of the papers retrieved were reviewed for additional publications. Eligible studies had to meet the following criteria: 1) original research study, 2) written in English, 3) reporting on adult (older than 16 years of age) patients infected with HIV who 4) were being prescribed self-administered ART for chronic HIV infection, 5) using a quantitative method to assess adherence to ART, and 6) reporting a statistical association between a potential predictor/correlate and adherence. No geographical restrictions were applied. We excluded studies that focused exclusively on the following specific populations: drug users, prison inmates, homeless individuals, and patients with psychiatric disorders.

We considered the following sociodemographic predictors/correlates of adherence: age, sex, and financial constraints. Financial constraints were defined as being unemployed or having an income level in the lowest category as defined within a particular study. Treatment-related predictors/correlates were: duration of ART, number of prescribed antiretroviral pills per day (that is, pill burden), daily dosing frequency, and whether or not the regimen contained a protease inhibitor (PI). Disease-related predictors/correlates included CD4 cell count and time since HIV diagnosis. We also investigated interpersonal predictors/correlates: social support, HIV stigma and trust or satisfaction with the HIV care provider. Finally, patient-related predictors/correlates were current substance use (alcohol and drugs), depressive symptoms, adherence self-efficacy (the extent to which patients believe that they will be able to adhere), motivation to adhere, locus of control (the extent to which individuals believe that they can control events that affect them), concerns about adverse effects of ART, and beliefs in the necessity or utility of ART.

Two authors (NL and PN) independently extracted data from each study that fulfilled the inclusion criteria, using a scoring sheet. The following information was extracted: name of the first author, year of publication, sample size, country in which the study was conducted, year study was started, adherence assessment method (self-report, electronic monitoring devices (EMD), pharmacy refill, pill count), and whether patients were initiating, restarting, or switching an ART regimen, or were already on ART, and the potential predictors or correlates of adherence. Factors that were assessed at the same time as the adherence measurement were considered to be correlates, and factors assessed prior to the adherence measurement were considered to be predictors. Because we included studies conducted in any country around the world, we categorized countries according to the United Nations Human Development Index (HDI) [10] during the year the study was started into low (HDI ≤ 0.50), medium (HDI of 0.50 to 0.79), and high (HDI ≥ 0.80) development countries.

When data from the same study were reported in multiple publications, we selected the publication that had the largest sample size and/or reported the largest number of predictors/correlates. If the study evaluated adherence at multiple time points, the value of the first measurement was used to avoid dependence. When relationships between predictors/correlates and adherence were reported for discrete subgroups within a single study, the groups were included as independent samples.

The quality of the reporting of the included studies was assessed using the 22 items recommended by the Strengthening the Reporting of Observational Studies in Epidemiology (STROBE) statement [11]. Items fulfilling the STROBE statement were considered positive and items not fulfilling the statement were as-considered negative.

### Statistical analysis

We used the standardized mean difference (SMD) as the common effect size to express the magnitude of the association between predictors/correlates and adherence. If studies did not provide the SMD, we calculated it from r, correlation coefficient means and standard deviations, odds ratios, *t*-test, χ^2^ test, or F-statistic, contingency table data, or exact *P* values [12]. When studies reported an insignificant association without data, we assigned it an SMD of 0.001. We adjusted the SMD using the small sample size bias correction prior to analysis. SMD values of 0.2, 0.5, and 0.8 were interpreted as small, medium. and large effects, respectively [13].

Predictors/correlates were selected for quantitative pooling if 10 or more independent effect sizes could be calculated. Random effect models with inverse variance weights were used to aggregate individual effect sizes into pooled effect estimates with 95% confidence limits (CI), using the SPSS macro MeanES from Lipsey and Wilson [12],[14].

We examined whether the effect sizes differed significantly across levels of potential moderators of the predictor-adherence relationship, if there was heterogeneity across studies (I^2^ > 50%) and sufficient data (k > 4 in each subgroup) to support these analyses [15]. The following study design features were investigated as potential moderators: whether the factor was a predictor or correlate, whether the adherence assessment method was self-report (versus all other methods) or EMD (versus all others), whether the study was conducted in a high HDI country (versus medium and low HDI country), and whether patients were already on ART (versus initiating, restarting, or switching ART). For the moderator analyses, subgroup analyses were performed by grouping effect sizes by study design feature and assessing heterogeneity between groups using the between-group Q statistic (Q-between) within a mixed effects model using the method of moments estimation. If Q-between is significantly greater than the heterogeneity within groups due to error (Q-within), this indicates that a moderator variable explains a significant proportion of the total heterogeneity in effect sizes. The moderator analyses were conducted using the SPSS macro MetaF from Lipsey and Wilson [12],[14].

## Results

**Figure 1:**
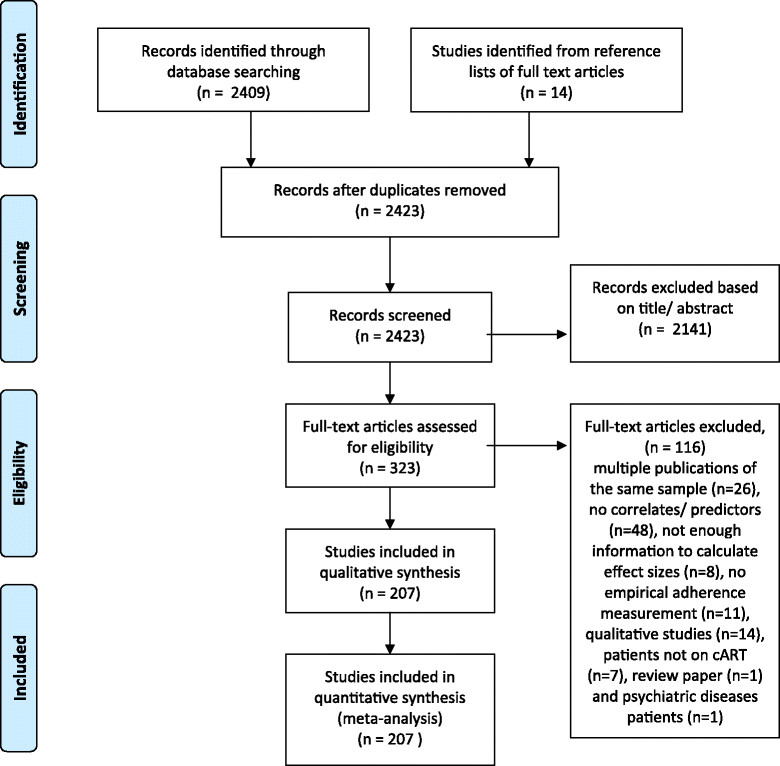
**Flow diagram of the study selection process.**

Figure 1 shows the study selection process. In total, 207 studies were included in our analysis, reporting on 103,836 patients [16]-[222]. Two hundred studies consisted of one independent sample for calculating effect sizes, five studies consisted of two samples and two studies consisted of three samples, resulting in a total of 216 independent samples (k = 216). A total of 67% (k = 145) of the samples reported on correlates of adherence, and 33% (k = 71) on predictors. A total of 54% (k = 117) of the samples included patients already on ART and 46% (k = 99) included patients who were (re)starting or switching. The following adherence assessment methods were used: 77% self-report (k = 166), 11% EMD (k = 23), 8% (k = 18) a pharmacy refill-based measure and 4% (k = 9) a pill count-based measure. A total of 67% (k = 145) of the samples originated from countries with a high HDI, 19% (k = 40) from countries with a medium HDI, and 14% (k = 31) from countries with a low HDI. For characteristics of the included studies, see Additional file 1.

Locus of control and motivation to adhere were excluded from our analysis because fewer than 10 independent effect sizes could be calculated for these two predictors/correlates.

**Figure 2:**
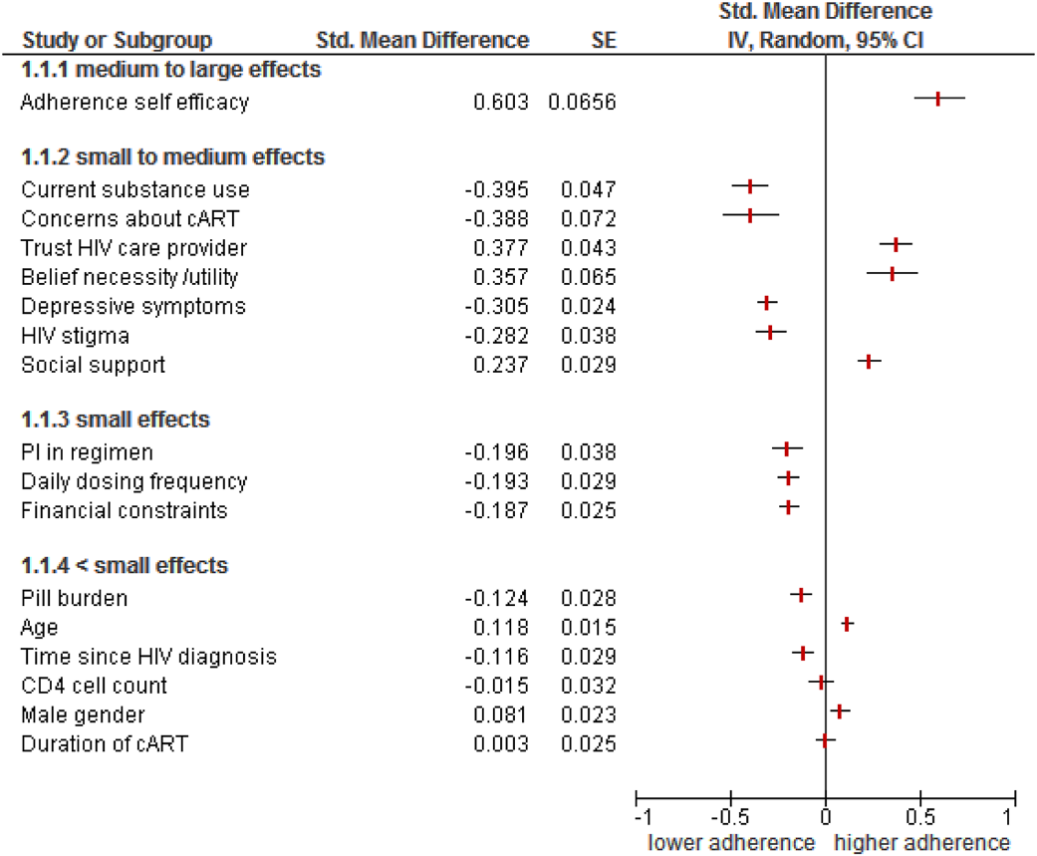
**Predictors/correlates of adherence to anti-retroviral therapy (ART).**

The strongest association with adherence was found for the predictor/correlate adherence self-efficacy, with the pooled effect size being medium to large (SMD = 0.603, 95% CI 0.47 to 0.73, k = 39, *P* = 0.001; Figure 2 (1.1.1)). The following predictors/correlates were significantly associated with adherence, with their effect sizes being small to medium: current substance use (SMD = -0.395, 95% CI -0.49 to -0.30, k = 80, *P* = 0.001), concerns about ART (SMD = -0.389, 95% CI -0.53 to -0.25, k = 14, *P* = 0.001), trust/satisfaction with the HIV healthcare provider (SMD = 0.377, 95% CI 0.29 to 0.46, k = 30, *P* = 0.001), beliefs about the necessity/utility of ART (SMD = 0.357, 95% CI 0.23 to 0.49, k = 25, p = 0.001), depressive symptoms (SMD = -0.305, 95% CI -0.35 to -0.26, k = 90, *P* = 0.001), HIV stigma (SMD = -0.282, 95% CI -0.36 to -0.21, k = 47, *P* = 0.001) and social support (SMD = 0.237, 95% CI 0.18 to 0.29, k = 67, *P* = 0.001). The predictors/correlates yielding small to medium effects are shown in Figure 2 (1.1.2).

The following predictors/correlates were significantly associated with adherence with their effect sizes being small: being prescribed a PI-containing regimen (SMD = -0.196, 95% CI -0.27 to -0.12, k = 26, *P* = 0.001), daily dosing frequency (SMD = -0.193, 95% CI -0.25 to -0.14, k = 29, *P* = 0.001) and financial constraints (SMD = -0.187, 95% CI -0.24 to -0.14, k = 110, *P* = 0.001). The predictors/correlates with small effect sizes are shown in Figure 2 (1.1.3).

The following predictors/correlates were significantly associated with adherence but their effect sizes were very small: pill burden (SMD = -0.124, 95% CI -0.18 to -0.07, k = 57, *P* = 0.001), age (SMD = 0.118, 95% CI 0.089 to 0.147, k = 158, *P* = 0.001), time since HIV diagnosis (SMD = -0.116, 95% CI -0.17 to -0.06, k = 57, *P* = 0.001) and male gender (SMD = 0.081, 95% CI 0.037 to 0.12, k = 142, *P* = 0.001). The predictors/correlates with very small effect sizes are shown in Figure 2 (1.1.4). Two predictors/correlates were not significantly associated with adherence: CD4 cell count (SMD = -0.015, 95% CI -0.079 to 0.048, k = 67, *P* = 0.64) and duration of ART (SMD = 0.003, 95% CI -0.047 to 0.052, k = 51, *P* = 0.92).

For forest plots of the individual studies examining predictors/correlates, see Additional file 2. For the scoring of included studies according to the items of the STROBE statement, see Additional file 3.

**Figure 3:**
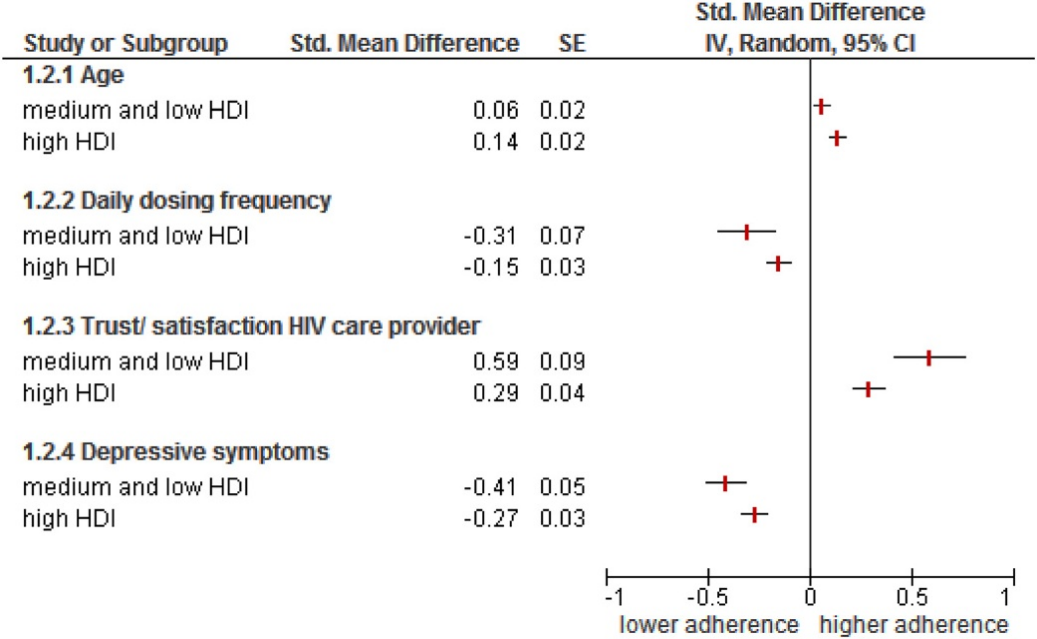
**Countries Human Development Index (HDI) as moderator of the predictor-adherence relationship.**

The study design feature “HDI of the country in which the study was conducted” was significantly associated with four predictors/correlates. Trust/satisfaction with the HIV care provider was more strongly associated with adherence in countries with a low or medium HDI than in countries with a high HDI (Figure 3 (1.2.3); Q-between = 8.04, *P* = 0.005). Daily dosing frequency was more strongly and negatively associated with adherence in countries with a medium or low HDI than in countries with a high HDI (Figure 3 (1.2.2); Q-between = 3.88, *P* = 0.049). Older age was associated with higher adherence in countries with a high HDI but not in countries with a medium or low HDI (Figure 3 (1.2.1); Q-between = 5.16, *P* = 0.02). Depressive symptoms were more strongly associated with lower levels of adherence in countries with a medium or low HDI than in countries with a high HDI (Figure 3 (1.2.4); Q-between = 4,38, *P* = 0.04).

**Figure 4:**
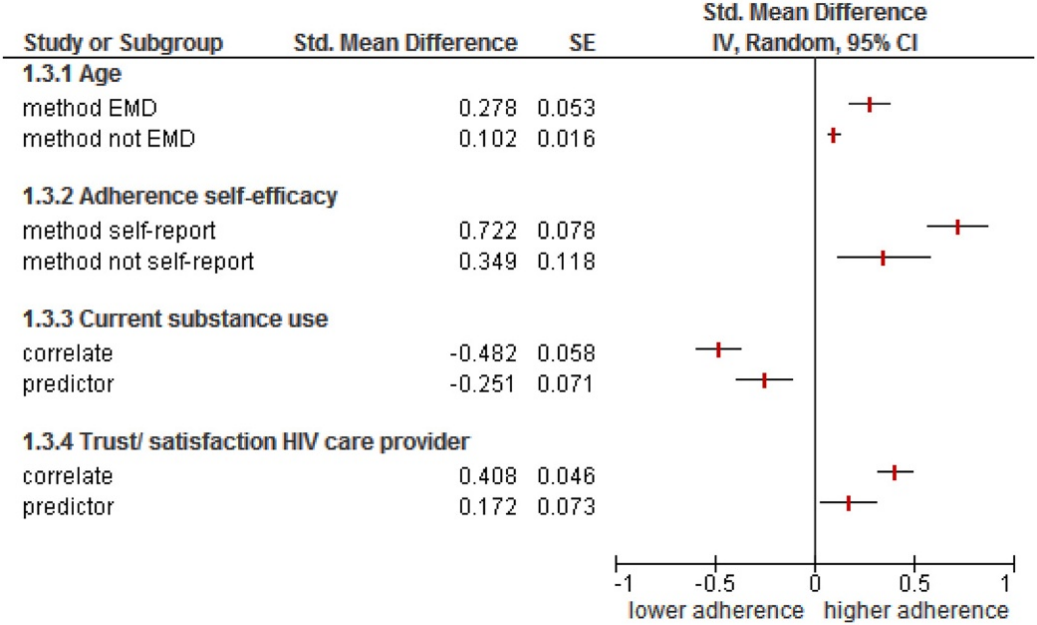
**Adherence assessment method and design as moderators of the predictor-adherence relationship.**

The adherence assessment method was significantly associated with two predictors/correlates. Adherence self-efficacy was more strongly associated with adherence in studies using self-report as adherence assessment method than in studies using another method (Figure 4 (1.3.2); Q-between = 6.94, *P* = 0.008). Older age was more strongly associated with adherence in studies using EMD as adherence assessment method than in studies using another method (Figure 4 (1.3.1); Q-between = 10.12, *P =* 0.002).

Whether investigated factors were predictors or correlates yielded two significant associations. Trust/satisfaction with the HIV care provider was more strongly related with adherence if assessed as a correlate than if assessed as a predictor (Figure 4 (1.3.4); Q-between =7.44, *P* = 006). Current substance use was more strongly and negatively related with adherence if assessed as a correlate than as a predictor (Figure 4 (1.3.3); Q-between = 6.34, *P* = 0.012).

## Discussion

In our meta-analysis based on 207 papers reporting on 103,836 patients, we obtained a comprehensive overview of the relative importance of various predictors/correlates of adherence to ART. Adherence to ART was most strongly associated with patients’ beliefs, that is, adherence self-efficacy beliefs, concerns about adverse effects of ART, and beliefs about the necessity/utility of ART, and also with current substance use, trust or satisfaction with the HIV care provider, depressive symptoms, HIV stigma, and social support. Aspects of regimen complexity, such as daily dosing frequency, pill burden, and whether the regimen included a PI, had smaller, albeit significant effects.

Our findings are consistent with a recent meta-analysis of patients with various long-term medical conditions, showing that patients’ beliefs have an important influence on medication adherence [223]. Results are also consistent with previous studies showing that substance use, depressive symptoms, and HIV stigma are associated with lower levels of adherence, and trust or satisfaction with the healthcare provider are associated with higher levels of adherence [7],[8],[180],[224]. These results should be encouraging to HIV care providers, as they suggest several avenues for intervention that could result in improved adherence. In particular, patients’ adherence-related beliefs and the relationship between the patient and their HIV care provider are factors that are in principle modifiable, and thus possible targets for adherence-enhancing interventions. Eliciting and addressing patients' beliefs and promoting an improved relationship between patient and healthcare provider have previously been associated with improved levels of adherence [225],[226].

This study has several limitations. We aimed to provide a global overview of the relative importance of various predictors/correlates of adherence to ART. Therefore, several predictors/correlates were aggregated into broad categories, for example, social support, HIV stigma, trust or satisfaction with the HIV healthcare provider, financial constraints, and substance use. Within these broad categories, distinct types of predictors/correlates may have a different impact on adherence, which would have remained undetected in the present global analysis; for example, alcohol use could have a different impact on adherence compared with cocaine use.

Another limitation is that the search was performed using a single database (PubMed) only, and included only published English-language papers. This may have influenced our results.

However, this meta-analysis has also several strengths. It provides a comprehensive overview of predictors/correlates of adherence to ART, with quantitative estimates of their impact. Moreover, this meta-analysis provides information about the relative importance of predictors/correlates.

Papers were included without restriction to geographical region, adherence assessment method, or study design. This probably resulted in a consequently large heterogeneity in effect sizes for most predictors/correlates. Guidelines for the reporting of meta-analyses of observational studies have recommended using broad inclusion criteria and then performing analyses relating design features to outcomes [227]. We thus conducted meta-regression analyses to explore the impact of study design features on predictors/correlates of adherence.

The study design feature “HDI of the country in which the study was conducted” was significantly associated with four predictors/correlates. Trust/satisfaction with the HIV healthcare provider had a stronger positive effect on adherence in countries with a low or medium HDI than in countries with a high HDI. A possible explanation for this is that in countries with low or medium HDI, patients are more dependent on their HIV healthcare provider for information, support, and care. Conversely, in countries with a high HDI, patients usually have more extensive access to healthcare providers and to information about health, HIV, and ART, making them less dependent on their HIV healthcare provider. There could also be cultural differences in the relationship between patients and healthcare providers, with the healthcare providers having more authority in low or medium income countries.

Daily dosing frequency was more strongly and negatively associated with adherence in countries with a medium or low HDI than in countries with a high HDI. One possible explanation is that achieving or maintaining high levels of adherence is more challenging in low or medium HDI countries. The added challenge of more frequent daily dosing could therefore more easily result in lower levels of adherence in these countries. However, this explanation is in contrast to results from previous studies showing higher levels of adherence in low income countries than in high income countries [3],[228].

Older age was associated with higher adherence in countries with a high HDI but not in countries with a medium or low HDI. This finding could simply reflect the fact that studies from low and medium HDI countries included few older patients. Finally, depressive symptoms were more strongly and negatively associated with adherence in countries with a medium or low HDI than in countries with a high HDI.

A remarkable finding was the limited effect of the adherence assessment method on effect sizes. Self-reports are known to overestimate adherence. We expected to find stronger associations between predictors/correlates and adherence in studies using EMDs than in studies using self-reports because EMDs are usually considered to be a more valid adherence assessment method [229]. A possible explanation for the limited effect of the adherence assessment method could be that although self-reports overestimate adherence, the rank order of patients on an adherence scale is similar for self-report and electronic monitoring, thus yielding similar associations.

Duration of ART was not related to adherence in the present meta-analysis. It is usually assumed that adherence declines with increasing length time on treatment. However, a recent study investigating the natural history of changes in adherence to ART over time has shown that the decline is non-linear, with substantial heterogeneity across studies [230]. In view of these results and the fact that the present meta-analysis included both patients already on ART and patients (re)starting ART, the absence of a relation between duration of ART and adherence is not surprising.

## Conclusions

This meta-analysis of predictor/correlates of ART showed that adherence was strongly related to patients’ adherence-related beliefs. These findings suggest that adherence-enhancing interventions should target psychological factors such as self-efficacy and necessity/concerns beliefs about ART. Additionally, simplification of regimens may have smaller, albeit significant effects.

## Authors’ contributions

All authors contributed to the design of the study. NL and PN performed database searches, study selection, and data extraction. NL performed quality assessment. PN conducted the meta-analyses and the moderator analyses. All authors reviewed and interpreted the study findings. All authors were involved in writing the manuscript. All authors read and approved the final version before submission.

## Additional files

## Electronic supplementary material

Additional file 1: Characteristics of included studies.(PDF 121 KB)

Additional file 2: Forest plots of individual studies examining predictors/correlates.(PDF 432 KB)

Additional file 3: Scoring of studies according to Strengthening the Reporting of Observational Studies in Epidemiology (STROBE) criteria.(PDF 270 KB)

Below are the links to the authors’ original submitted files for images.

Authors’ original file for figure 1

Authors’ original file for figure 2

Authors’ original file for figure 3

Authors’ original file for figure 4
