# Supplementary material for: Predictors and correlates of adherence to combination antiretroviral therapy (ART) for chronic HIV infection: a meta-analysis
Source: BMC Med. 2014 Aug 21;12:142. doi: 10.1186/s12916-014-0142-1 (PMC4148019; doi:10.1186/s12916-014-0142-1)
Supplement: Supplementary file 1 — Additional file 1: Characteristics of included studies.(PDF 121 KB) [file 12916_2014_142_MOESM1_ESM.pdf]

Additional file 1: Characteristics of included studies

Reference	N	Country	HDI	Treatment status	Adherence assessment method	Predictor/correlate	Factors
Adewuya 2010 (16)	182	Nigeria	.42	on cART	Self-report	correlate	Age, Male gender, Financial constraints Social support, Time since HIV diagnosis Duration of cART
Alakija 2010 (17)	253	Nigeria	.51	on cART	Self-report	correlate	Age, Male gender, Duration of cART, Financial constraints
Amberbir 2008 (18)	383	Ethiopia	.41	start	Self-report	correlate	Depressive symptoms, Social support
Andrade 2003 (19)	80	USA	.93	on cART	Pharmacy refill	correlate	Age, Male gender, Current substance use, CD4 cell count, depressive symptoms, duration of cART
Ammasari 2004 (20)	135	Italy	.93	on cART	Self-report	correlate	Age, Male gender, Current substance use, Financial constraints, Depressive symptoms
Anuradha 2011 (21)	250	India	.55	on cART	Pharmacy refill	correlate	Age, Male gender, Current substance use Social Support, Adherence Self Efficacy Duration of cART, CD4 count, Financial Constraints, Depressive Symptoms
Aragones 2011 (22)	847	Cuba	.78	on cART	Self-report	correlate	Age, Male gender, Time since HIV diagnosis Duration of cART, Pill burden, Daily dosing frequency, Trust/satisfaction health care provider, Self efficacy
Arrivillaga 2009 (23)	269	Colombia	.69	on cART	Self-report	correlate	Financial constraints
Babson 2013 (24)	129	USA	.91	on cART	Pill count	correlate	Current substance use
Barclay 2007 (25)	185	USA	.96	on cART	Electronic monitoring	predictor	Male gender, Time since HIV diagnosis, Current substance use, Social Support, Adherence Self Efficacy, Necessity/Utility of cART, Duration of cART, Financial Constraints, Depressive Symptoms
Beach 2005 (26)	2338	USA	.95	on cART	Self-report	correlate	Trust/satisfaction health care provider
Bell 2007 (27)	59	Malawi	.44	on cART	Electronic monitoring	predictor	Male gender, Duration of cART
Berhe 2012 (28)	348	Ethiopia	.36	on cART	Self-report	correlate	HIV stigma, Social support, depressive symptoms, current substance use, financial constraints, CD4 cell count.
Bianco 2010 (29)	242	USA	.91	on cART	Self-report	correlate	Age, Male gender, Time since HIV diagnosis, Social Support, Financial Constraints, Depressive Symptoms
Birbeck 2011 (30)	488	Zambia	.47	Start/switch	Pharmacy refill	predictor	Age, Male gender, HIV Stigma, Financial Constraints

Reference	N	Country	HDI	Treatment status	Adherence assessment method	Predictor/correlate	Factors
Blackstock 2012 (31)	175	USA	.90	on cART	Self-report	correlate	Age, Male gender , Trust/Satisfaction with health care provider, Time since HIV diagnosis, Current substance use, Duration of cART, Daily dosing frequency, Financial Constraints
Boarts 2006 (32)	57	USA	.96	on cART	Self-report	predictor	Age, Depressive symptoms
De Boer 2008 (33)	391	Netherlands	.94	on cART	Self-report	correlate	Necessity/utility cART, Concerns cART
Bonolo 2005 (34)	306	Brazil	.81	on cART	Self-report	correlate	Male gender, Financial constraints, HIV stigma Concurrent substance use, Pill burden, CD4 cell count, Time since HIV diagnosis
Bottonari 2012 (35)	192	USA	.90	on cART	Self-report	correlate	Age, Male gender, Current substance use, Financial Constraints, Depressive Symptoms
Boyer 2011 (36)	2117	Cameroon	.52	on cART	Self-report	correlate	Age, Male gender, Time since HIV diagnosis, Current substance use, HIV Stigma, Social Support, Duration of cART, CD4 cell count, Financial Constraints
Brigido 2001 (37)	182	Brasil	.78	on cART	Self-report	correlate	Age, Male gender, CD4 cell count, Financial Constraints
Brown 2013 (38)	116	USA	.91	on cART	Self-report	correlate	Age, Male gender, Financial constraints, duration of cART, Pill burden, Adherence self efficacy, Necessity/utility cART
Busher 2012 (39)	99	USA	.91	Start/switch	Self-report	predicto	Male gender, Pill burden, Dosing frequency, CD4 cell count, Financial constraints
Cahn 2004 (40)	315	Argentina, Italy, Brazil, Canada, Thailand, Mexico	.80	Start/switch	Self-report	predictor	Age, Pill Burden, PI containing regimen, Daily dosing frequency
Cambiano 2010 (41)	2060	UK	.86	on cART	Pharmacy refill	predictor	Age, Duration of cART
Campbell 2010 (42)	122	Guatamala	.56	on cART	Pharmacy refill	correlate	Duration of cART, Pill burden
Campos 2010 (43)	293	Brasil	.78	Start/switch	Self-report	predictor	Age, Male gender, Current substance use, CD4 cell count, Financial Constraints, Depressive Symptoms
Carballo 2004 (44)	235	Spain	.95	on cART	Self-report	correlate	Age, Financial constraints, Time since HIV diagnosis, Pill burden
Cardarelli 2008 (45)	103	USA	.91	on cART	Self-report	correlate	Age, Male gender, Current substance use, HIV Stigma, Social Support, Pill Burden, Depressive Symptoms
Carmody 2003 (46)	67	Brasil	.79	on cART	Pharmacy refill	correlate	Age, Male gender, CD4 cell count, Pill burden

Reference	N	Country	HDI	Treatment status	Adherence assessment method	Predictor/correlate	Factors
Catz 2000 (47)	72	USA	.93	on cART	Self-report	correlate	Age, Male gender, Time since HIV diagnosis, Current substance use, Social Support, Adherence Self Efficacy, Pill Burden, Duration of cART, Financial Constraints, Depressive Symptoms
Cauldbeck 2009 (48)	53	India	.60	on cART	Self-report	correlate	Age, Male gender , Time since HIV diagnosis Pill Burden, Duration of cART, Financial Constraints
Cha 2008 (49)	215	USA	.91	on cART	Self-report	correlate	Depressive symptoms, Adherence self efficacy, Social support
Chesney 2000 (50)	75	USA	.94	on cART	Self-report	correlate	Current substance use, Depressive Symptoms Financial Constraints, Social Support, Adherence Self Efficacy
Colbert 2013 (51)	302	USA	.93	on cART	Electronic monitoring	predictor	Age, male gender, Financial constraints, Adherence self efficacy, Pill burden
Cooper 2010 (52)	87	UK	.85	Start/switch	Electronic monitoring	predictor	Age, Male gender, Daily dosing frequency, CD4 cell count, Necessity/utility cART, Concerns about cART
Cooper 2011 (53)	234	UK	.86	Start/switch	Self-report	predictor	Necessity/utility cART, Concerns about cART
Corless 2013 (54)	1571	USA	.93	on cART	Self-report	correlate	Trust/satisfaction health care provider
Dale 2014 (55)	138	USA	.91	on cART	Self-report	correlate	CD4 cell count
Diabate (56)	591	Cote d’Ivoire	.48	on cART	Self-report	predictor	Age, Social support, Pill burden, CD4 cell count
Dlamini 2009 (58)	698	Lesotho, Malawi, South Africa, Swaziland, Tanzania	.48	on cART	Self-report	predictor	HIV stigma
DiIorio 2007 (57)	236	USA	.96	on cART	Self-report	correlate	Age, Male gender, Financial constraints, Adherence self-efficacy, Depressive symptoms Social support, HIV stigma, Trust/satisfaction health care provider
Do 2010 (59)	300	Botswana	.63	on cART	Self-report	correlate	Age, Male gender, Financial constraints Duration of cART, Pill burden, Depressive symptoms, Concurrent substance use, HIV stigma
Dorz 2003 (60)	109	Italy	.93	on cART	Self-report	correlate	Age, Male gender, Financial constraints
Duggan 2009 (61)	129	USA	.91	on cART	Self-report	correlate	Age, Male gender, Financial Constraints

Reference	N	Country	HDI	Treatment status	Adherence assessment method	Predictor/correlate	Factors
Duong 2001 (62)	149		.94	on cART	Self-report	correlate	Age, Male gender, Necessity/Utility of cART, Adherence self efficacy, Social support
Durante 2003 (63)	63	USA	.94	on cART	Self-report	correlate	Trust/Satisfaction with health care provider, HIV Stigma, Social Support
Eholie 2007 (64)	308	Cote dIvoire	.48	on cART	Self-report	correlate	Age, Male gender, Financial constraint, Time since HIV diagnosis, PI containing regimen
Elul 2013 (65)	1408	Rwanda	.45	on cART	Self-report	correlate	Male gender, Financial constraints, CD4 cell count, Current substance use.
Etard 2007 (66)	158	Senegal	.46	Start/switch	Pharmacy refill	predictor	Age, Male gender, Duration of cART<br>PI containing regimen
Etienne 2010 (67)	921	Kenya, Uganda, Zambia, Nigeria, Rwanda	.42	on cART	Self-report	correlate	Male gender, Concurrent substance use, Depressive symptoms
Ettenhofer 2009 (68)	431	USA	.95	on cART	Electronic monitoring	predictor	Age
Falang 2012 (69)	461	Nigeria	.45	on cART	Self-report	correlate	Age, Male gender, Current substance use, HIV Stigma, Pill Burden, Daily dosing frequency, Financial Constraints
Farley 2010 (70)	222	Nigeria	.51	on cART	Pharmacy refill	correlate	Depressive Symptoms
Fatima 2013 (71)	199	Brasil	.78	Start/switch	Self-report	predictor	Age, Financial constraints, HIV stigma, Current substance use, Pill burden, CD4 cell count, Depressive symptoms
Feldman 2013 (72)	2399	USA	.95	on cART	Self-report	correlate	Age, Male gender, Depressive symptoms
Ferguson 2002 (73)	149	USA	.94	on cART	Self-report	predictor	Male gender, Social Support
Finnoccharion 2011 (74)	168	USA	.95	on cART	Electronic monitoring	predictor	Age, Male gender, Current substance use, Social Support, Adherence Self Efficacy, PI containing regimen, Daily dosing frequency, CD4 cell count, Financial Constraints, Depressive Symptoms
Fong 2003 (75)	161	Hong Kong	.92	on cART	Self-report	correlate	Age, Male gender, Time since HIV diagnosis, Duration of cART, Daily dosing frequency, PI containing regimen, Pill burden, HIV stigma
Ford 2010 (76)	207	South Africa	.67	Start/switch	Self-report	correlate	Age, Male gender, CD4 cell count
Frain 2009 (77)	75	USA	.91	on cART	Self-report	correlate	Trust/Satisfaction with health care provider, HIV Stigma, Concerns about cART, CD4 cell count, Financial Constraints

Reference	N	Country	HDI	Treatment status	Adherence assessment method	Predictor/correlate	Factors
Franke 2010 (78)	134	Peru	.72	Start/switch	Self-report	predictor	Age, Male gender, CD4 cell count, Financial constraints, Depressive symptoms, Social support, HIV stigma, Adherence self efficacy
Garcia 2006 (81)	182	Brazil	.81	on cART	Self-report	correlate	Age, Male gender, Social Support, Duration of cART, Financial Constraints
Gauchet 2007 (82)	122	France	.93	on cART	Self-report	correlate	Age, Male gender , Trust/Satisfaction with health care provider, Time since HIV diagnosis, Pill Burden, Necessity/Utility of cART, Concerns about cART
Gao 2000 (80)	72	USA	.94	on cART	Self-report	correlate	Age, Daily dosing frequency, Financial Constraints
Gay 2011 (83)	302	USA	.95	on cART	Self-report	correlate	Age, Male gender, Time since HIV diagnosis, Pill Burden, PI containing regimen, CD4 cell count, Financial Constraints
Gionotti 2012 (84)	2114	Italy	.87	on cART	Self-report	correlate	Age, CD4 cell count, Pill burden, Daily dosing frequency
Gibbie 2007 (85)	72	Australia	.96	on cART	Self-report	correlate	Age, Social Support, Financial Constraints, Depressive Symptoms
Giday 2010 (86)	510	Ethiopia	.33	on cART	Self-report	correlate	Age, Male gender, Financial constraints, HIV stigma, Social support
Gifford 2000 (87)	133	USA	.93	on cART	Self-report	correlate	Age, Male gender, Social Support, Adherence Self Efficacy, Pill Burden, Daily dosing frequency, CD4 cell count, Depressive Symptoms
Glass 2010 (88)	5664	Switzerland	.95	on cART	Self-report	predictor	Age, Male gender, Social Support, PI containing regimen, CD4 cell count
Godin 2005 (89)	376	Canada	.96	on cART	Self-report	predictor	Age, Male gender, CD4 cell count, Trust/Satisfaction with health care provider, Time since HIV diagnosis, Social Support, Adherence Self Efficacy
Gokarn 2012 (90)	300	India	.61	on cART	Self-report	correlate	Age, Male gender, Financial constraints, CD4 cell count, HIV stigma,
Golin 2002 (91)	117	USA	.93	on cART	Electronic monitoring	predictor	Age, Male gender, Time since HIV diagnosis, Current substance use, Social Support, Adherence Self Efficacy, Pill Burden, Duration of cART, Daily dosing frequency, CD4 cell count, Financial Constraints, Depressive Symptoms

Reference	N	Country	HDI	Treatment status	Adherence assessment method	Predictor/correlate	Factors
Gonzalez 2004 (92)	90	USA	.95	on cART	Self-report	correlate	Age, Male gender, Time since HIV diagnosis, Current substance use, Social Support, Pill Burden, Duration of cART, CD4 cell count, Financial Constraints, Depressive Symptoms
Gonzalez 2007 (93)	325	USA	.93	on cART	Electronic monitoring	predictor	Age, Pill Burden, Necessity/Utility of cART, Concerns about cART, Financial Constraints, Depressive Symptoms
Gordillo 1999 (94)	366	Spain	.89	on cART	Self-report	correlate	Age, Social Support, CD4 cell count, Depressive Symptoms
Graham 2007 (95)	87	USA	.96	on cART	Pharmacy refill	correlate	Male gender, Financial constraints
Hanif 2013 (96)	632	Brasil	.81	on cART	Self-report	Orrelate	Male gender, Social support, Financial constraints, Depressive symptoms, duration of cART
Hansana 2013 (97)	346	Laos	.52	on cART	Self-report	orrelate	Age, Time since HIV diagnosis
Haubrich 1999 (98)	173	USA	.93	on cART	Self-report	predictor	Age, Male gender, Trust/Satisfaction with health care provider, Current substance use
Heckman 2004 (99)	272	USA	.93	on cART	Self-report	correlate	Age, Male gender, Trust/Satisfaction with health care provider, Current substance use Social Support, Financial Constraints, Depressive Symptoms
Holmes 2007 (100)	116	USA	.96	on cART	Electronic monitoring	predictor	Financial constraints, HIV stigma, Trust/satisfaction health care provider, Depressive symptoms, Social support
Holzemer 1999 (102)	420	USA	.93	on cART	Self-report	correlate	Age, Male gender, Social Support, Financial Constraints, Depressive Symptoms
Horne 2007 (103)	117	United Kingdom	.93	on cART	Self-report	predictor	Age, Time since HIV diagnosis, Pill Burden Necessity/Utility of cART, Concerns about cART CD4 cell count, Depressive Symptoms
Howard 2002 (104)	161	USA	.93	on cART	Electronic monitoring	predictor	Age, Current substance use, PI containing regimen, Duration of cART, Daily dosing frequency, CD4 cell count, Financial Constraints
Huang 2013 (105)	199	China	.69	on cART	Self-report	correlate	Adherence self efficacy
Huynh 2013 (106)	142	USA	.90	on cART	Self-report	correlate	Male gender, Social support, Financial constraints, Time since HIV diagnosis, CD4 cell count.
Ickovics 2002 (107)	93	USA	.94	Start/switch	Self-report	predictor	Age, Male gender, Current substance use, Adherence Self Efficacy, CD4 cell count, Financial Constraints, Depressive Symptoms

Reference	N	Country	HDI	Treatment status	Adherence assessment method	Predictor/correlate	Factors
Ingersoll 2004 (108)	120	USA	.95	on cART	Self-report	correlate	Age, Male gender
Jacquet 2010 (109)	2065	Benin, Cote dIvoire, Mali	.38	on cART	Self-report	correlate	Age, Male gender, CD4 cell count Duration of cART, Concurrent substance use
Johnson 2003 (110)	2765	USA	.94	on cART	Self-report	correlate	Age, Male gender, Time since HIV diagnosis, Current substance use, Social Support, Pill Burden, Daily dosing frequency, CD4 cell count, Financial Constraints, Depressive Symptoms, Trust/Satisfaction with health care provider, Adherence Self Efficacy
Johnson 2012 (111)	293	USA	.90	on cART	Self-report	correlate	Depressive symptoms, Necessity/ utility cART, concerns about cART
Juday 2011 (112)	461	USA	.96	on cART	Self-report	correlate	Age, Male gender, Time since HIV diagnosis, Current substance use, Pill Burden, Financial Constraints, Depressive Symptoms
Kacanek 2010 (113)	177	USA	.91	on cART	Self-report	predictor	Age, Male gender, Financial constraints, Duration of cART, Concurrent substance use, Social support
Kalichman 1999 (114)	182	USA	.93	on cART	Self-report	correlate	Age, Male gender, CD4 cell count, Trust/Satisfaction with health care provider, Time since HIV diagnosis, Social Support, Financial Constraints
Kalichman 2003 (115)	255	USA	.91	on cART	Self-report	correlate	Depressive symptoms, Social support Current substance use
Kalichman 2008 (116)	145	USA	.91	on cART	Unannounced pillcount	predictor	Age, Depressive symptoms, Social support HIV stigma, Current substance use, Time since HIV diagnosis
Kalichman 2010 (117)	188	USA	.91	on cART	Unannounced pillcount	predictor	Age, Male gender, CD4 cell count, Current substance use, HIV Stigma, Financial Constraints, Depressive Symptoms
Kamau 2011 (118)	354	Kenya	.50	on cART	Self-report	correlate	Age, Male gender, Financial Constraints
Kerr 2012 (119)	288	Thailand	.67	on cART	Self-report	correlate	Age, Male gender, Time since HIV diagnosis, Current substance use, Adherence Self Efficacy Pill Burden, Duration of cART, Financial Constraints, Depressive Symptoms
King 2012 (120)	326	USA	.90	on cART	Self-report	correlate	Age, male gender, Depressive symptoms, Current substance use
Kleeberger 2001 (121)	539	USA	.93	on cART	Self-report	correlate	Age, Current substance use, Pill Burden, CD4 cell count, Depressive Symptoms

Reference	N	Country	HDI	Treatment status	Adherence assessment method	Predictor/correlate	Factors
Kumar 2009 (122)	1192	USA	.93	on cART	Self-report	correlate	Age, Male gender, Social Support, Financial Constraints, Depressive Symptoms
Kunutsor 2010 (123)	967	Uganda	.42	on cART	Pill count	predictor	Age, Male gender, Duration of cART
Kyser 2011 (124)	528	USA	.91	on cART	Self-report	correlate	Age, Male gender, Financial constraints CD4 cell count, Time since HIV diagnosis, PI containing regimen, Daily dosing frequency Depressive symptoms, Concurrent substance use
Ladefoged 2012 (125)	46	Greenland	.96	on cART	Self-report	correlate	Age, Male gender, duration of cART, Financial constraints, Social support, Current substance use
Lazo 2007 (126)	1944	USA	.93	Start/switch	Self-report	predictor	Age, Current substance use, PI containing regimen, CD4 cell count, Financial Constraints, Depressive Symptoms
Leombruni 2009 (127)	130	Italy	.87	on cART	Self-report	correlate	Age, Male gender, Time since HIV diagnosis, CD4 cell count, Depressive Symptoms
Leserman 2008 (128)	105	USA	.91	on cART	Self-report	correlate	Age, Male gender, Financial constraints, Current substance use
Li 2010 (129)	386	Thailand	.78	on cART	Self-report	correlate	Age, Male gender, Time since HIV diagnosis, HIV Stigma, Social Support, Financial Constraints, Depressive Symptoms
Li 2011 (130)	202	China	.67	on cART	Self-report	correlate	HIV Stigma, Adherence Self Efficacy
Luszczynska 2007 (131)	104	India	.60	on cART	Self-report	correlate	Age, Male gender, Social Support, Adherence Self Efficacy
Lyimo 2014 (132)	158	Tanzania	.47	on cART	Self-report	predictor	Current substance use, HIV stigma
Lyman 2009 (133)	189	USA	.91	Start/switch	Electronic monitoring	predictor	Adherence Self Efficacy
Maggiolo 2002 (134)	597	Italy	.92	on cART	Self-report	correlate	Daily dosing frequency, duration of cART, Pill burden
Malow 2013 (135)	194	Haiti	.45	on cART	Self-report	correlate	Age, Male gender, Depressive symptoms, Necessity/utility cART, Concerns about cART
Maqutu 2011 (136)	688	South Africa	.62	on cART	Pill count	predictor	Age, Male gender, CD4 cell count
Mannheimer 2002 (137)	1095	USA	.93	on cART	Self-report	predictor	Age, Male gender, Pill Burden, PI containing regimen, CD4 cell count
Mathews 2002 (138)	164	USA	.93	on cART	Electronic monitoring	predictor	Age, Male gender, Current substance use, Adherence Self Efficacy, Necessity/Utility of cART
McAllister 2013 (139)	335	Australia	.93	on cART	Self-report	correlate	Age, Male gender, Financial constraints

Reference	N	Country	HDI	Treatment status	Adherence assessment method	Predictor/correlate	Factors
McDonnell Holstad 2006 (102)	120	USA	.96	on cART	Self-report	correlate	Male gender, Time since HIV diagnosis, Current substance use, Duration of cART, Financial Constraints
Mellins 2003 (140)	62	USA	.93	on cART	Self-report	predictor	Age, Time since HIV diagnosis, Current substance use, HIV Stigma, Adherence Self Efficacy, Pill Burden, CD4 cell count
Molassiotis 2002 (141)	139	Hong Kong	.90	on cART	Self-report	correlate	Age, Male gender, Time since HIV diagnosis, Social Support, Adherence Self Efficacy, PI containing regimen, Daily dosing frequency, Depressive Symptoms
Moralejo 2006 (142)	143	Spain	.95	on cART	Pharmacy refill	correlate	Age, Male gender, Trust/Satisfaction with health care provider, Current substance use Adherence Self Efficacy, Depressive Symptoms
Mugavero 2009 (143)	289	USA	.94	on cART	Self-report	predictor	Age, Male gender, Current substance use, Depressive Symptoms
Murphy 2004 (144)	115	USA	.95	on cART	Self-report	correlate	Age, Male gender, Trust/Satisfaction with health care provider, Current substance use, Social Support, Depressive Symptoms
Murri 2001 (145)	140	Italy	.90	on cART	Self-report	predictor	Age, Male gender, Current substance use, Depressive Symptoms
Nakimuli 2009 (146)	122	Uganda	.42	on cART	Self-report	correlate	Age, Male gender, Duration of cART
Nakimuli 2013 (147)	400	Uganda	.45	on cART	Pill count	predictor	Age, Male gender, Financial constraints, Adherence self efficacy, Social support
Negash 2013 (148)	355	Ethiopia	.36	on cART	Self-report	correlate	Male gender, HIV stigma, Depressive symptoms
Nel 2013 (149)	94	South Africa	.62	on cART	Self-report	correlate	Depressive symptoms
Nelsen 2013 (150)	244	USA	.90	on cART	Self-report	correlate	Age, Male gender, Time since HIV diagnosis, CD4 cell count
Nelson 2013 (151)	1676	USA	.94	Start/switch	Pharmacy refill	predictor	PI containing regimen
Nieuwkerk 2001 (152)	160	Netherlands	.92	on cART	Self-report	predictor	Age, Male gender, CD4 cell count
Nillson Schonnesson 2006 (153)	144	Sweden	.94	on cART	Self-report	predictor	Age, Male gender, Trust/Satisfaction with health care provider, Time since HIV diagnosis, Social Support, Adherence Self Efficacy, Pill Burden, PI containing regimen, Necessity/Utility of cART, Daily dosing frequency, Concerns about cART, CD4 cell count, Depressive Symptoms
Nozaki 2011 (154)	518	Zambia	.43	on cART	Self-report	correlate	Age, Male gender, Duration of cART, Social support, HIV stigma, Financial constraints

Reference	N	Country	HDI	Treatment status	Adherence assessment method	Predictor/correlate	Factors
OConnor 2013 (155)	5295	USA	.94	Start/switch	Self-report	predictor	Age, Male gender, Pill burden, Daily dosing frequency, PI containing regimen
Oku 2014 (156)	393	Nigeria	.46	on cART	Self-report	correlate	Age, Male gender, Financial constraints
Orrell 2003 (157)	289	South Africa	.67	Start/switch	Pill count	predictor	Age, Male gender, CD4 cell count, Daily dosing frequency, Financial constraints, PI containing regimen
Oguyi 2007 (158)	97	Uganda	.49	on cART	Electronic monitoring	predictor	Age, Male gender, Current substance use, CD4 cell count, Financial Constraints, Depressive Symptoms
Parutti 2006 (159)	171	Italy	.93	Start/switch	Pharmacy refill	predictor	Age, Male gender, Current substance use, Pill Burden, PI containing regimen, Financial Constraints
Paterson 2000 (160)	99	USA	.94	on cART	Electronic monitoring	predictor	Age, Male gender, Financial Constraints, Depressive Symptoms, Necessity/utility cART
Pefura 2013 (161)	889	Cameroon	.48	on cART	Self-report	correlate	Male gender, Current substance use, CD4 cell count, Depressive symptoms, duration of cART
Peltzer 2010 (162)	519	South Africa	.60	on cART	Self-report	correlate	Age, Male gender, Financial constraints, CD4 cell count, Time since HIV diagnosis, Depressive symptoms, Concurrent substance use, HIV stigma, Social support
Peretti 2006 (163)	1809	France	.94	on cART	Self-report	correlate	Age, Time since HIV diagnosis, Current substance use, HIV Stigma, Financial Constraints
Pinheiro 2002 (164)	195	Brasil	.75	on cART	Self-report	correlate	Age, Male gender, Adherence Self Efficacy, Necessity/Utility of cART, Duration of cART, Daily dosing frequency, Financial Constraints
Plankey 2009 (165)	1671	USA	.93	on cART	Self-report	predictor	Age, Current substance use
Poguette 2013 (166)	74	USA	.90	on cART	Electronic monitoring	predictor	Age, Male gender, Depressive symptoms, Pill burden, duration of cART
Power 2003 (167)	73	USA	.94	on cART	Self-report	correlate	Age, Male gender, Social support
Pratt 2001 (168)	260	USA	.93	on cART	Self-report	correlate	Daily dosing frequency, Depressive symptoms, Male gender, Pill burden, Social support, Current substance use
Protopopescu 2009 (169)	1010	France	.92	on cART	Self-report	predictor	Age, Male gender, Trust/Satisfaction with health care provider, Time since HIV diagnosis, Social Support, Pill Burden, PI containing regimen, Daily dosing frequency, Financial Constraints, Depressive Symptoms, CD4 cell count, Concurrent substance use

Reference	N	Country	HDI	Treatment status	Adherence assessment method	Predictor/correlate	Factors
Raboud 2011 (170)	779	Canada	.91	on cART	Self-report	correlate	Age, Male gender, Financial constraints, Daily dosing frequency, Depressive symptoms, Social support, HIV stigma, Time since HIV diagnosis, Duration of cART, PI containing regimen
Ramadhani 2007 (171)	150	Tanzania	.53	on cART	Self-report	correlate	Age, Male gender, Depressive symptoms, HIV stigma, Duration of cART, CD4 cell count
Rao 2012 (172)	720	USA	.91	on cART	Self-report	correlate	HIV stigma, Depressive symptoms
Reynolds 2004 (173)	980	USA	.94	Start/switch	Self-report	correlate	Social Support, Depressive Symptoms
Remien 2007 (174)	200	Brazil	.81	On cART	Self-report	correlate	Age, Male gender, Financial constraints, Pill burden, Daily dosing frequency, Social support, Trust/satisfaction health care provider, Adherence self-efficacy, Necessity/utility cART, Concerns about cART
Rintamaki 2006 (175)	204	USA	.94	on cART	Self-report	correlate	Age, Male gender, HIV Stigma, Pill Burden
Rodrigues 2012 (176)	150	India	.55	on cART	Pill count	predictor	Age, Male gender, CD4 cell count, Financial constraints, Time since HIV diagnosis, duration of cART
Rougemont 2009 (177)	219	Cameroon	.52	Start/switch	Pharmacy refill	predictor	Male gender, HIV Stigma, Daily dosing frequency, CD4 cell count
Safren 2005 (178)	304	India	.60	on cART	Self-report	correlate	Age, Male gender, Financial constraints Duration of cART
Sasaki 2012 (179)	157	Zambia	.43	on cART	Self-report	correlate	Age, Male gender, Time since HIV diagnosis, HIV stigma
Sayles 2009 (180)	142	USA	.91	on cART	Self-report	correlate	Age, Male gender, CD4 cell count, Time since HIV diagnosis, Financial constraints, HIV stigma
Schneider 2004 (181)	554	USA	.95	on cART	Self-report	correlate	Age, Provider trust /satisfaction
Seguy (182)	689	Brazil	.81	on cART	Pharmacy refill	correlate	Age, Male gender, Time since HIV diagnosis, CD4 cell count, PI containing regimen, Pill burden
Sellier 2006 (183)	39	France	.96	on cART	Self-report	correlate	Age, Male gender, Financial constraints, Duration of cART, Pill burden, HIV stigma
Servellen van 2002 (184)	182	USA	.94	on cART	Self-report	correlate	Age, Male gender, Trust/Satisfaction with health care provider, Social Support, Financial Constraints, Depressive Symptoms
Shah 2007 (185)	276	India	.61	on cART	Self-report	correlate	Age, Financial constraints, Pill burden, Social support, Adherence self efficacy
Sherr 2008, 2010 (186, 187)	502	United Kingdom	.95	on cART	Self-report	correlate	Age, Male gender, HIV Stigma, Social Support, Financial Constraints, Depressive Symptoms

Reference	N	Country	HDI	Treatment status	Adherence assessment method	Predictor/correlate	Factors
Shuter 2008 (188)	64	USA	.96	on cART	Electronic monitoring	predictor	Age, Male gender, Current substance use, CD4 cell count, Depressive Symptoms
Silva 2009 (189)	412	Brasil	.70	on cART	Self-report	correlate	Age, Male gender, Duration of cART, Time since HIV infection, Pill burden, Financial constraints, Depressive symptoms
Simoni 2002 (190)	50	USA	.93	on cART	Self-report	correlate	Age, Male gender, Time since HIV diagnosis, Current substance use, Social Support, Adherence Self Efficacy, Depressive Symptoms
Simoni 2012 (191)	1809	USA	.91	on cART	Electronic monitoring	predictor	Age, Male gender, Current substance use, Financial Constraints, Depressive Symptoms
Singh 1999 (192)	123	USA	.93	on cART	Pharmacy refill	predictor	Age, Financial Constraints, Social Support
Sodergard 2006 (193)	946	Sweden	.96	on cART	Self-report	correlate	Age, Concurrent substance use, Duration of cART, Social support, Daily dosing frequency
Spire 2002 (194)	445	France	.92	on cART	Self-report	predictor	Age, Male gender, Trust/Satisfaction with health care provider, Time since HIV diagnosis, Current substance use, HIV Stigma, Social Support, Necessity/Utility of cART, CD4 cell count, Financial Constraints
Stirratt 2006 (195)	215	USA	.96	on cART	Electronic monitoring	predictor	Adherence self-efficacy, HIV stigma, Pill burden, Depressive symptoms
Sullivan 2007 (196)	5887	USA	.94	on cART	Self-report	correlate	Age, Current substance use, Pill Burden, PI containing regimen, Duration of cART, Depressive Symptoms
Sumari 2011 (197)	202	Netherlands	.90	on cART	Pharmacy refill	correlate	Age, Male gender, Time since HIV diagnosis, HIV Stigma, PI containing regimen, Necessity/Utility of cART, Duration of cART, Concerns about cART, Depressive Symptoms
Tadios 2006 (198)	431	Ethiopia	.40	on cART	Self-report	correlate	Age, Financial constraints, HIV stigma, Necessity/utility cART, Adherence self efficacy, Depressive symptoms, Provider trust/ satisfaction
Tedaldi 2012 (199)	539	USA	.95	on cART	Self-report	predictor	Male gender, Current substance use, Time since HIV diagnosis, Depressive symptoms
Teixera 2012 (200)	144	Brasil	.81	on cART	Self-report	correlate	Current substance use
Thrasher 2008 (201)	1886	USA	.93	on cART	Self-report	correlate	Age, Male gender, Trust/Satisfaction with health care provider, Time since HIV diagnosis, Social Support, Pill Burden, Necessity/Utility of cART, CD4 cell count, Depressive Symptoms

Reference	N	Country	HDI	Treatment status	Adherence assessment method	Predictor/correlate	Factors
Tiyou 2010 (202)	306	Ethiopia	.35	on cART	Self-report	correlate	Male gender, Social Support, Financial Constraints
Tran 2013 (203)	1016	Vietnam	.59	on cART	Self-report	correlate	Financial constraints, duration of cART, Adherence self efficacy
Trotta 2003 (204)	596	Italy	.93	on cART	Self-report	correlate	Age, CD4 cell count, PI containing regimen
Ubbiali 2008 (205)	478	Italy	.87	on cART	Self-report	correlate	Male gender
Ukwe 2010 (206)	299	Nigeria	.45	on cART	Self-report	predictor	Age, Male gender, Trust/Satisfaction with health care provider, Current substance use, Necessity/Utility of cART, Financial Constraints
Unge 2010 (207)	352	Kenya	.54	Start/on cART	Self-report	predictor	Age, Male gender, Duration cART, Current substance use, Financial constraints, Social support, HIV stigma
Uuskula 2012 (208)	144	Estonia	.81	on cART	Self-report	correlate	Age, Male gender, Time since HIV diagnosis, Current substance use, Pill Burden, Necessity/Utility of cART, Duration of cART, Daily dosing frequency, Concerns about cART, CD4 cell count, Financial Constraints, Depressive Symptoms
Venkatesh 2010 (209)	198	India	.53	on cART	Self-report	correlate	Age, Male gender, Current substance use, Duration of cART, CD4 cell count, Financial Constraints, Depressive Symptoms
Vyavahakar 2007 (210)	224	USA	.96	on cART	Self-report	correlate	Age, Social Support, Financial Constraints
Wagner 2002 (211)	61	USA	.94	on cART	Electronic monitoring	predictor	Age, Male gender, Adherence Self Efficacy, Pill Burden, PI containing regimen, Necessity/Utility of cART, Daily dosing frequency, CD4 cell count, Financial Constraints
Wagner 2012 (212)	182	USA	.91	on cART	Electronic monitoring	predictor	Age, Time since HIV diagnosis, Current substance use, HIV Stigma, Financial Constraints, Depressive Symptoms
Waite 2008 (213)	204	USA	.94	on cART	Self-report	correlate	HIV Stigma
Wanchu 2006 (214)	200	India	.60	On cART	Self-report	correlate	Male gender
Wang 2007 (215)	181	China	.77	on cART	Self-report	correlate	Age, Male gender, Financial constraints, Duration of cART, Necessity/utitily cART, Concurrent substance use, Trust /satisfaction health care provider, HIV stigma

Reference	N	Country	HDI	Treatment status	Adherence assessment method	Predictor/correlate	Factors
Wasti 2010 (216)	330	Nepal	.45	on cART	Self-report	correlate	Age, Male gender, Current substance use, HIV Stigma, Pill Burden, Necessity/Utility of cART, Duration of cART, Concerns about cART, Financial Constraints
Watt 2010 (217)	340	Tanzania	.40	on cART	Self-report	correlate	Adherence self efficacy, Provider trust/satisfaction
Weaver 2005 (218)	322	USA	.95	on cART	Electronic monitoring	predictor	Age, Time since HIV diagnosis, Social Support, Pill Burden, Financial Constraints
Webb 2009 (219)	168	USA	.94	on cART	Self-report	correlate	Depressive Symptoms
Weidle 2006 (220)	987	Uganda		Start/switch	Pill count	predictor	Age, Male gender, Depressive symptoms, CD4 cell count, Concurrent substance use
Woods 2009 (221)	79	USA	.96	on cART	Electronic monitoring	predictor	Age, Time since HIV diagnosis, Pill Burden, Daily dosing frequency, Duration of cART, CD4 cell count
Woodward 2013 (222)	136	USA	.95	on cART	Self-report	correlate	Social support, Depressive symptoms
Yun 2005 (223)	506	USA	.93	on cART	Pharmacy refill	correlate	Age, Male gender, Current substance use, Financial Constraints
