# Supplementary material for: Predictors and correlates of adherence to combination antiretroviral therapy (ART) for chronic HIV infection: a meta-analysis
Source: BMC Med. 2014 Aug 21;12:142. doi: 10.1186/s12916-014-0142-1 (PMC4148019; doi:10.1186/s12916-014-0142-1)

Additional file 1: Forest plots of individual studies examining predictors/correlates

Adherence self-efficacy

Study or Subgroup	Std. Mean Difference	SE	Weight	Std. Mean Difference IV, Random, 95% CI
Mellins 2003	-0.05	0.36		-0.05 [-0.76, 0.66]
Kerr 2012	0	0.18		0.00 [-0.35, 0.35]
Wagner 2002	0	0.26		0.00 [-0.51, 0.51]
Barclay 2 2007	0	0.3		0.00 [-0.59, 0.59]
Tadios 2006	0	0.12		0.00 [-0.24, 0.24]
Mathews 2002	0.08	0.16		0.08 [-0.23, 0.39]
Anuradha 2012	0.1	0.36		0.10 [-0.61, 0.81]
Golin 2002	0.1	0.19		0.10 [-0.27, 0.47]
Simoni 2002	0.14	0.28		0.14 [-0.41, 0.69]
Johnson 2006	0.32	0.04		0.32 [0.24, 0.40]
Moralejo 2006	0.4	0.18		0.40 [0.05, 0.75]
Godin 2005	0.41	0.1		0.41 [0.21, 0.61]
Colbert 2013	0.43	0.19		0.43 [0.06, 0.80]
Nakimulli 2013	0.43	0.1		0.43 [0.23, 0.63]
Barclay 1 2007	0.44	0.17		0.44 [0.11, 0.77]
Stirratt 2006	0.45	0.14		0.45 [0.18, 0.72]
Li 2011	0.47	0.16		0.47 [0.16, 0.78]
Lynam 2006	0.47	0.15		0.47 [0.18, 0.76]
Reynolds 2002	0.52	0.11		0.52 [0.30, 0.74]
Ickovics 2002	0.63	0.22		0.63 [0.20, 1.06]
Pinheiro 2002	0.68	0.15		0.68 [0.39, 0.97]
Huang 2013	0.75	0.18		0.75 [0.40, 1.10]
Duong 2001	0.76	0.27		0.76 [0.23, 1.29]
Chesney 2000	0.77	0.25		0.77 [0.28, 1.26]
Shah 2007	0.78	0.14		0.78 [0.51, 1.05]
Remien 2007	0.78	0.21		0.78 [0.37, 1.19]
Gifford 2000	0.79	0.18		0.79 [0.44, 1.14]
Nilsson 2006	0.81	0.18		0.81 [0.46, 1.16]
Mollassiotis 2002	0.84	0.25		0.84 [0.35, 1.33]
Aragones 2011	0.89	0.08		0.89 [0.73, 1.05]
Luszczynska 2007	0.95	0.21		0.95 [0.54, 1.36]
Cha 2008	0.98	0.14		0.98 [0.71, 1.25]
Finnocchario 2011	1	0.16		1.00 [0.69, 1.31]
Catz 2000	1.06	0.32		1.06 [0.43, 1.69]
Watt 2010	1.11	0.17		1.11 [0.78, 1.44]
Brown 2013	1.15	0.21		1.15 [0.74, 1.56]
Franke 2010	1.15	0.46		1.15 [0.25, 2.05]
Tran 2013	1.19	0.08		1.19 [1.03, 1.35]
DiIorio 2007	1.63	0.15		1.63 [1.34, 1.92]

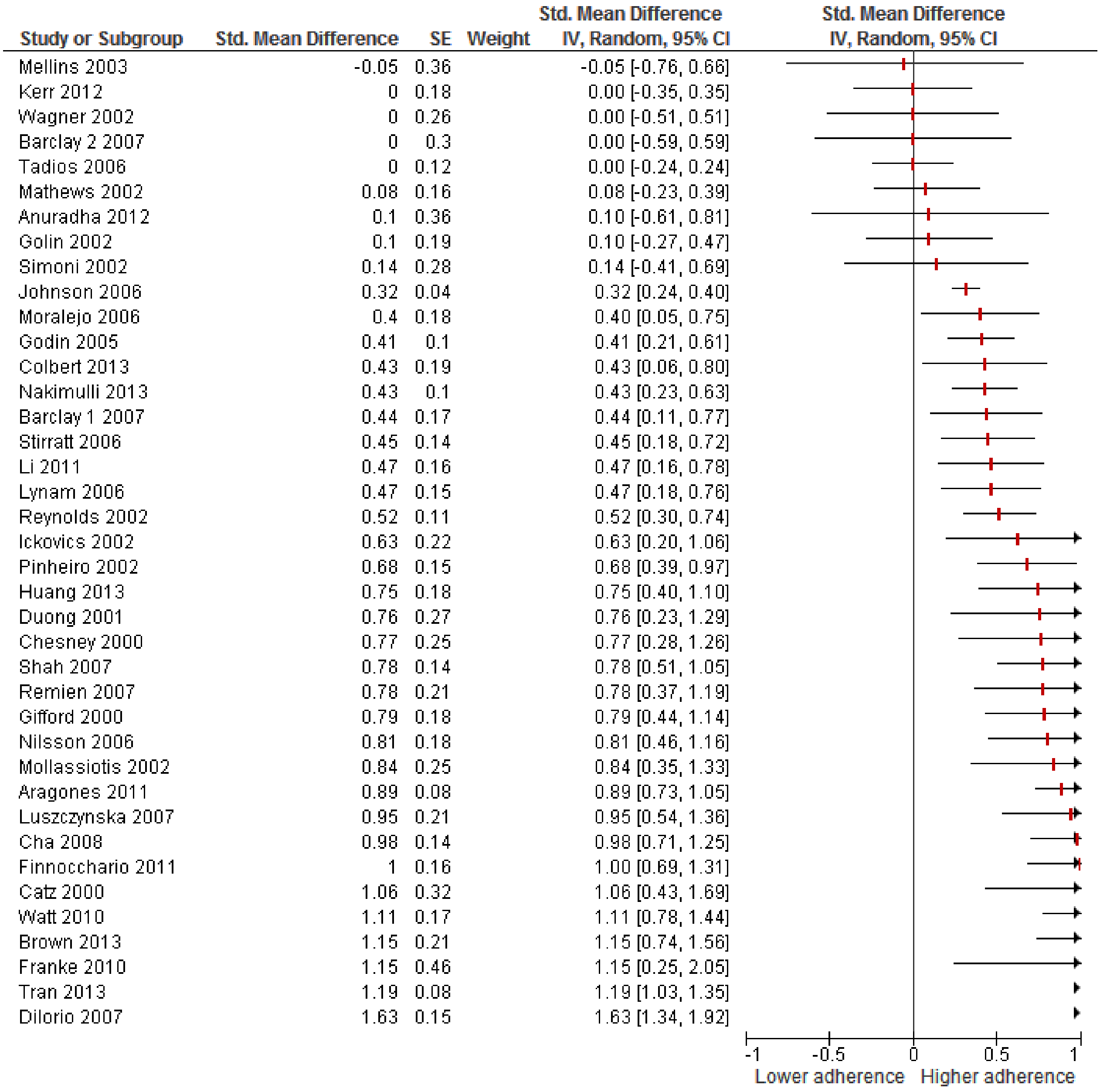

Current substance use

Study or Subgroup	Std. Mean Difference	SE	Weight	Std. Mean Difference IV, Random, 95% CI
Peltzer 2010	-2.41	0.14	1.3%	-2.41 [-2.68, -2.14]
Mellins 2003	-1.74	0.348	0.7%	-1.74 [-2.42, -1.06]
Berhe 2013	-1.3	0.12	1.3%	-1.30 [-1.54, -1.06]
Plankey 2009	-1.24	0.047	1.5%	-1.24 [-1.33, -1.15]
Bonolo 2005	-1.16	0.13	1.3%	-1.16 [-1.41, -0.91]
Wasti 2012	-1.04	0.135	1.3%	-1.04 [-1.30, -0.78]
Falang 2012	-1.01	0.112	1.3%	-1.01 [-1.23, -0.79]
Ladefoged 2012	-0.86	0.33	0.8%	-0.86 [-1.51, -0.21]
King 2012	-0.83	0.12	1.3%	-0.83 [-1.07, -0.59]
Venkatesh 2010	-0.71	0.149	1.2%	-0.71 [-1.00, -0.42]
Boyer 2011	-0.63	0.042	1.5%	-0.63 [-0.71, -0.55]
Howard 2002	-0.63	0.168	1.2%	-0.63 [-0.96, -0.30]
Heckman 2004	-0.62	0.126	1.3%	-0.62 [-0.87, -0.37]
Catz 2000	-0.62	0.252	1.0%	-0.62 [-1.11, -0.13]
Gonzalez 2004	-0.61	0.23	1.0%	-0.61 [-1.06, -0.16]
Ammassari 2004	-0.6	0.188	1.1%	-0.60 [-0.97, -0.23]
Chesney 2000	-0.54	0.25	1.0%	-0.54 [-1.03, -0.05]
Etienne 2010	-0.54	0.07	1.4%	-0.54 [-0.68, -0.40]
Finnocchario 2011	-0.53	0.165	1.2%	-0.53 [-0.85, -0.21]
Murphy 2004	-0.53	0.187	1.1%	-0.53 [-0.90, -0.16]
Fatima 2 2013	-0.53	0.2	1.1%	-0.53 [-0.92, -0.14]
Sodergard 2006	-0.51	0.07	1.4%	-0.51 [-0.65, -0.37]
Blackstock 2012	-0.5	0.165	1.2%	-0.50 [-0.82, -0.18]
Cardarelli 2008	-0.48	0.206	1.1%	-0.48 [-0.88, -0.08]
Pratt 2001	-0.46	0.13	1.3%	-0.46 [-0.71, -0.21]
Uuskula 2012	-0.45	0.164	1.2%	-0.45 [-0.77, -0.13]
Andrade 2013	-0.45	0.23	1.0%	-0.45 [-0.90, 0.00]
Haubrich 1999	-0.43	0.164	1.2%	-0.43 [-0.75, -0.11]
Teixera 2012	-0.43	0.17	1.2%	-0.43 [-0.76, -0.10]
Fatima 1 2013	-0.43	0.16	1.2%	-0.43 [-0.74, -0.12]
Yun 2005	-0.42	0.082	1.4%	-0.42 [-0.58, -0.26]
Golin 2002	-0.41	0.184	1.1%	-0.41 [-0.77, -0.05]
Barclay 1 2007	-0.41	0.184	1.1%	-0.41 [-0.77, -0.05]
Mollassiotis 2002	-0.41	0.184	1.1%	-0.41 [-0.77, -0.05]
Elul 2013	-0.39	0.06	1.4%	-0.39 [-0.51, -0.27]
Lyimo 2014	-0.39	0.16	1.2%	-0.39 [-0.70, -0.08]
Pefura 2013	-0.39	0.07	1.4%	-0.39 [-0.53, -0.25]
Leserman 2008	-0.39	0.2	1.1%	-0.39 [-0.78, 0.00]
Kalichman 2003	-0.38	0.21	1.1%	-0.38 [-0.79, 0.03]
Kleeberger 2001	-0.36	0.081	1.4%	-0.36 [-0.52, -0.20]
Peretti 3 2006	-0.33	0.101	1.4%	-0.33 [-0.53, -0.13]
Campos 2010	-0.33	0.122	1.3%	-0.33 [-0.57, -0.09]
Simoni 2012	-0.32	0.041	1.5%	-0.32 [-0.40, -0.24]
Jacquet 2010	-0.3	0.08	1.4%	-0.30 [-0.46, -0.14]
Kalichman 2010	-0.29	0.141	1.3%	-0.29 [-0.57, -0.01]

Std. Mean Difference IV, Random, 95% CI

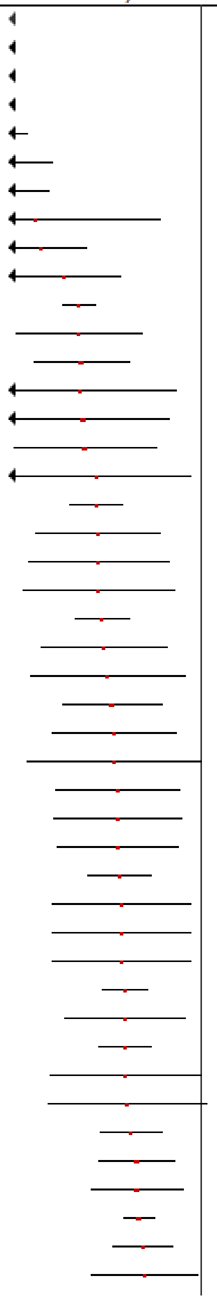

[illegible]	[illegible]	[illegible]	[illegible]
Mugavero 2009	-0.28	0.121	-0.28 [-0.52, -0.04]
Peretti 2 2006	-0.27	0.081	-0.27 [-0.43, -0.11]
Kyser 2011	-0.27	0.12	-0.27 [-0.51, -0.03]
Do 2010	-0.27	0.15	-0.27 [-0.56, 0.02]
Nakimulli 2013	-0.26	0.1	-0.26 [-0.46, -0.06]
Peretti 1 2006	-0.24	0.081	-0.24 [-0.40, -0.08]
Juday 2011	-0.23	0.101	-0.23 [-0.43, -0.03]
Holstad 2006	-0.22	0.181	-0.22 [-0.57, 0.13]
Spire 2002	-0.22	0.101	-0.22 [-0.42, -0.02]
Protopopescu 2009	-0.22	0.06	-0.22 [-0.34, -0.10]
Silva 2009	-0.21	0.1	-0.21 [-0.41, -0.01]
Kacanek 2010	-0.21	0.15	-0.21 [-0.50, 0.08]
Tedaldi 2012	-0.2	0.09	-0.20 [-0.38, -0.02]
Sullivan 2007	-0.2	0.02	-0.20 [-0.24, -0.16]
Lazo 2 2007	-0.2	0.06	-0.20 [-0.32, -0.08]
Ukwe 2010	-0.18	0.12	-0.18 [-0.42, 0.06]
Johnson 2003	-0.16	0.04	-0.16 [-0.24, -0.08]
Kumar 2009	-0.14	0.06	-0.14 [-0.26, -0.02]
Parruti 2006	-0.11	0.16	-0.11 [-0.42, 0.20]
Glass 2010	-0.11	0.02	-0.11 [-0.15, -0.07]
Wagner 2011	-0.1	0.14	-0.10 [-0.37, 0.17]
Shuter 2008	-0.08	0.26	-0.08 [-0.59, 0.43]
Anuradha 2012	-0.06	0.12	-0.06 [-0.30, 0.18]
Babson 2013	-0.03	0.18	-0.03 [-0.38, 0.32]
Bottonari 2012	-0.01	0.14	-0.01 [-0.28, 0.26]
Unge 2010	0	0.1	0.00 [-0.20, 0.20]
Mathews 2002	0	0.16	0.00 [-0.31, 0.31]
Kerr 2012	0	0.12	0.00 [-0.24, 0.24]
Simoni 2002	0	0.3	0.00 [-0.59, 0.59]
Barclay 2 2007	0	0.3	0.00 [-0.59, 0.59]
Oyugi 2007	0	0.2	0.00 [-0.39, 0.39]
Ickovics 2002	0.07	0.22	0.07 [-0.36, 0.50]
Murri 2001	0.14	0.18	0.14 [-0.21, 0.49]
Lazo 1 2007	0.21	0.08	0.21 [0.05, 0.37]
Weidle 2006	0.72	0.07	0.72 [0.58, 0.86]

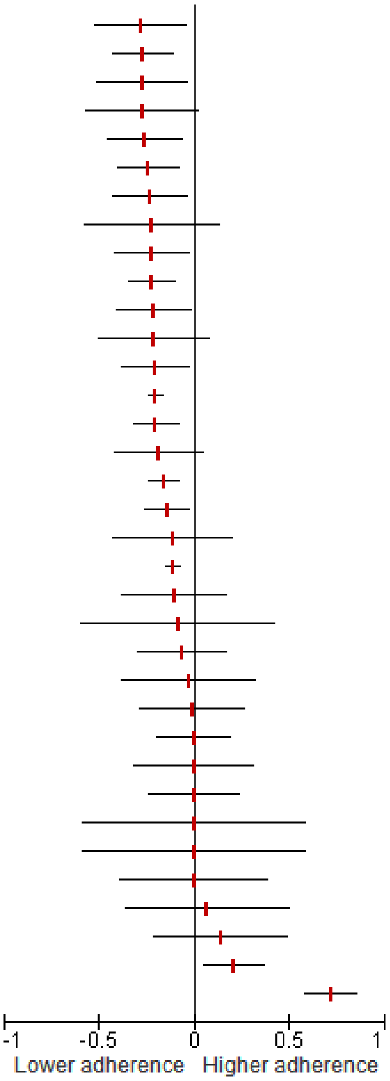

Concerns about cART

Study or Subgroup	Std. Mean Difference	SE	Weight	Std. Mean Difference IV, Random, 95% CI
Uuskula 2012	-0.76	0.171		-0.76 [-1.10, -0.42]
Malow 2013	-0.74	0.15		-0.74 [-1.03, -0.45]
Nilsson 2006	-0.72	0.17		-0.72 [-1.05, -0.39]
Horne 2007	-0.66	0.19		-0.66 [-1.03, -0.29]
Wasti 2012	-0.65	0.126		-0.65 [-0.90, -0.40]
Gonzalez 2007	-0.45	0.123		-0.45 [-0.69, -0.21]
Cooper 2010	-0.4	0.22		-0.40 [-0.83, 0.03]
Cooper 2011	-0.34	0.14		-0.34 [-0.61, -0.07]
de Boer 2008	-0.32	0.101		-0.32 [-0.52, -0.12]
Sumari 2011	-0.21	0.141		-0.21 [-0.49, 0.07]
Frain 2009	-0.08	0.24		-0.08 [-0.55, 0.39]
Gauchet 2007	-0.08	0.18		-0.08 [-0.43, 0.27]
Johnson 2012	-0.03	0.12		-0.03 [-0.27, 0.21]
Remien 2007	0	0.2		0.00 [-0.39, 0.39]

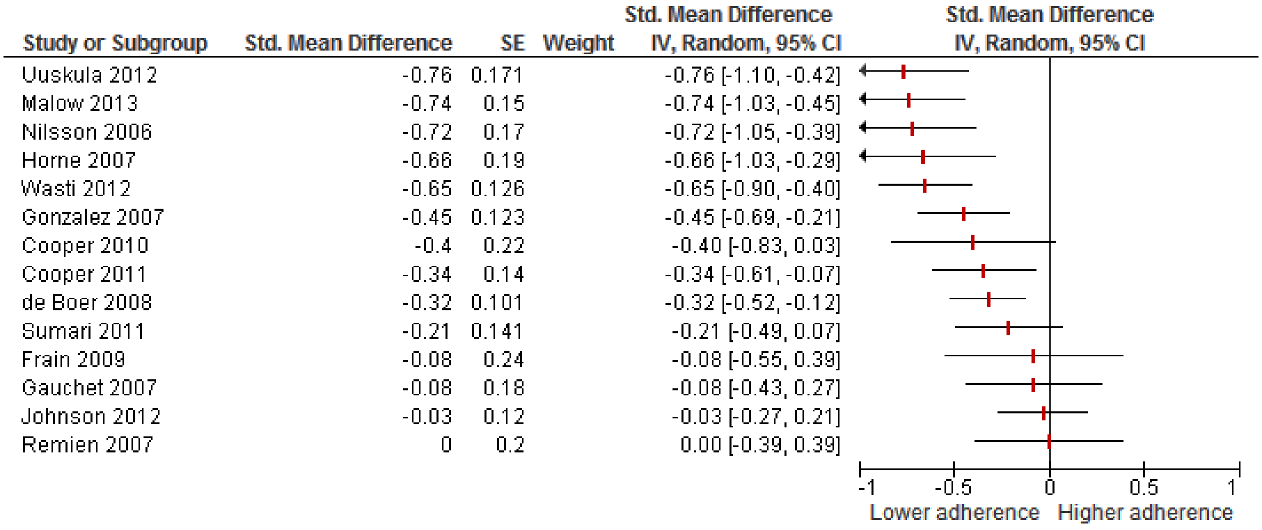

Trust /satisfaction HIV care provider

Study or Subgroup	Std. Mean Difference	SE	Weight	Std. Mean Difference IV, Random, 95% CI
Haubrich 1999	0	0.16		0.00 [-0.31, 0.31]
Godin 2005	0	0.1		0.00 [-0.20, 0.20]
Durante 2003	0	0.26		0.00 [-0.51, 0.51]
Murphy 2004	0.1	0.18		0.10 [-0.25, 0.45]
Thrasher 2008	0.1	0.04		0.10 [0.02, 0.18]
Holmes 2007	0.16	0.19		0.16 [-0.21, 0.53]
Protopopescu 2009	0.16	0.06		0.16 [0.04, 0.28]
Ukwe 2010	0.17	0.12		0.17 [-0.07, 0.41]
Servellen 2002	0.2	0.141		0.20 [-0.08, 0.48]
Spire 2002	0.24	0.101		0.24 [0.04, 0.44]
Kalichman 1999	0.27	0.141		0.27 [-0.01, 0.55]
Johnson 2006	0.28	0.04		0.28 [0.20, 0.36]
Dilorio 2007	0.28	0.13		0.28 [0.03, 0.53]
Corless 1 2013	0.28	0.05		0.28 [0.18, 0.38]
Schneider 2004	0.3	0.09		0.30 [0.12, 0.48]
Aragones 2011	0.3	0.08		0.30 [0.14, 0.46]
Beach 2005	0.35	0.04		0.35 [0.27, 0.43]
Nilsson 2006	0.36	0.163		0.36 [0.04, 0.68]
Moralejo 2006	0.4	0.163		0.40 [0.08, 0.72]
Golin 2002	0.41	0.184		0.41 [0.05, 0.77]
Corless 2 2013	0.43	0.2		0.43 [0.04, 0.82]
Heckman 2004	0.47	0.123		0.47 [0.23, 0.71]
Gauchet 2007	0.53	0.186		0.53 [0.17, 0.89]
Frain 2009	0.55	0.249		0.55 [0.06, 1.04]
Corless 3 2013	0.66	0.2		0.66 [0.27, 1.05]
Blackstock 2012	0.75	0.171		0.75 [0.41, 1.09]
Remien 2007	0.86	0.21		0.86 [0.45, 1.27]
Wang 2007	0.88	0.2		0.88 [0.49, 1.27]
Tadios 2006	0.99	0.13		0.99 [0.74, 1.24]
Watt 2010	1.02	0.23		1.02 [0.57, 1.47]

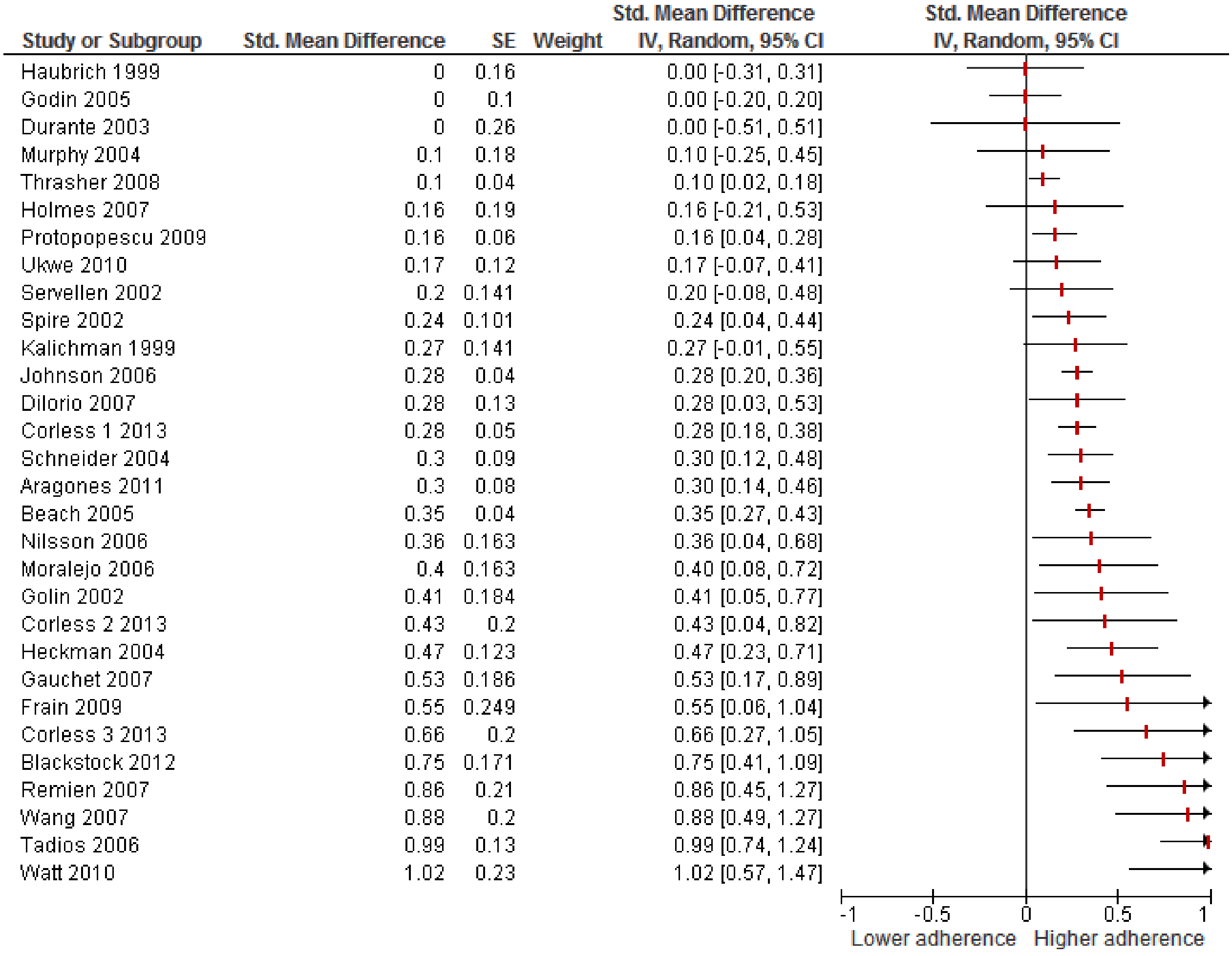

Belief necessity/utility of ART

Study or Subgroup	Std. Mean Difference	SE	Weight	Std. Mean Difference IV, Random, 95% CI
Ukwe 2010	-0.18	0.12		-0.18 [-0.42, 0.06]
Wagner 2002	0	0.26		0.00 [-0.51, 0.51]
Remien 2007	0	0.2		0.00 [-0.39, 0.39]
Johnson 2012	0.16	0.12		0.16 [-0.08, 0.40]
Thrasher 2008	0.22	0.04		0.22 [0.14, 0.30]
Uuskula 2012	0.23	0.161		0.23 [-0.09, 0.55]
Cooper 2010	0.25	0.22		0.25 [-0.18, 0.68]
Pinheiro 2002	0.25	0.141		0.25 [-0.03, 0.53]
Duong 2001	0.27	0.27		0.27 [-0.26, 0.80]
Cooper 2011	0.31	0.14		0.31 [0.04, 0.58]
Gonzalez 2007	0.32	0.122		0.32 [0.08, 0.56]
Sumari 2011	0.32	0.142		0.32 [0.04, 0.60]
French 2011	0.33	0.05		0.33 [0.23, 0.43]
de Boer 2008	0.37	0.102		0.37 [0.17, 0.57]
Brown 2013	0.47	0.19		0.47 [0.10, 0.84]
Gauchet 2007	0.49	0.185		0.49 [0.13, 0.85]
Horne 2007	0.5	0.186		0.50 [0.14, 0.86]
Nilsson 2006	0.54	0.166		0.54 [0.21, 0.87]
Spire 2002	0.56	0.104		0.56 [0.36, 0.76]
Barclay 2007	0.59	0.146		0.59 [0.30, 0.88]
Malow 2013	0.74	0.15		0.74 [0.45, 1.03]
Wasti 2012	0.8	0.129		0.80 [0.55, 1.05]
Mathews 2002	0.83	0.174		0.83 [0.49, 1.17]
Paterson 2000	0.86	0.28		0.86 [0.31, 1.41]
Wang 2007	0.9	0.2		0.90 [0.51, 1.29]

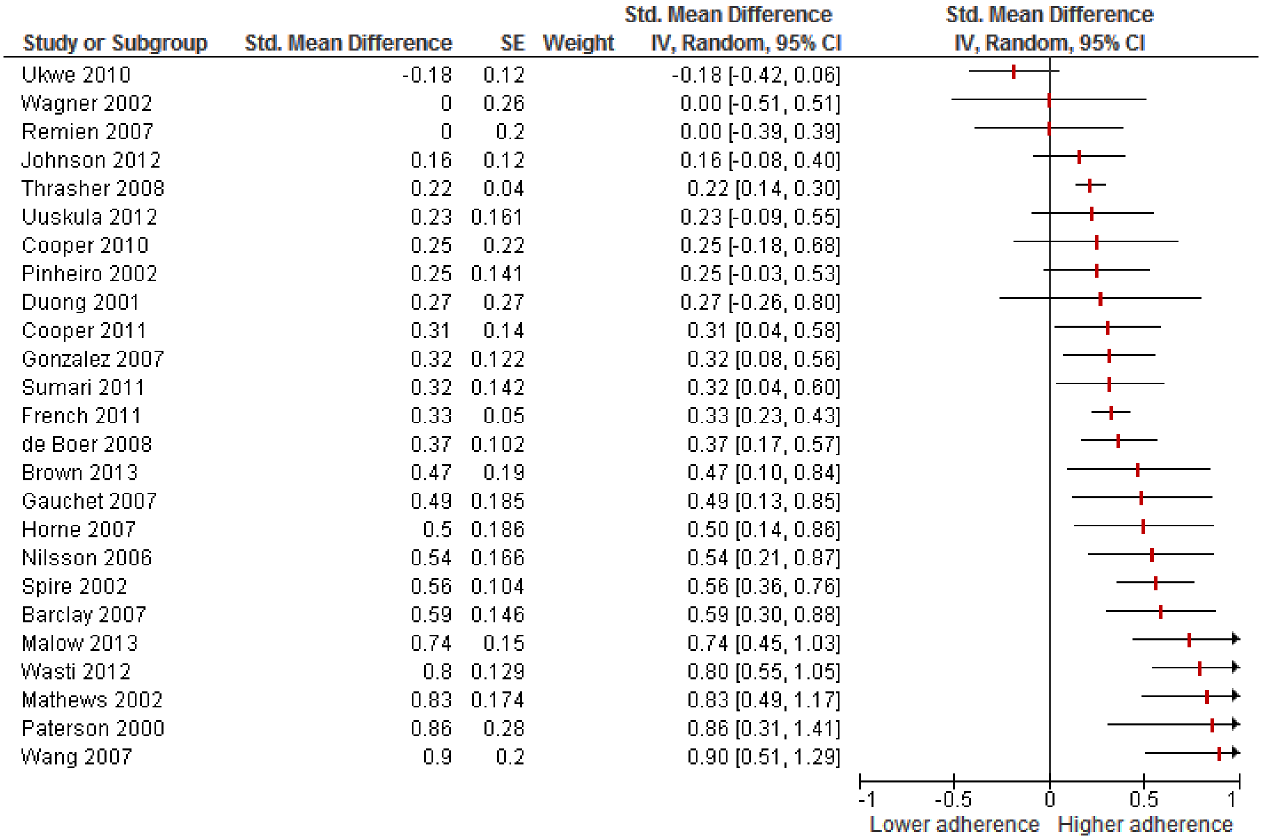

Depressive symptoms

Study or Subgroup	Std. Mean Difference	SE	Weight	Std. Mean Difference IV, Random, 95% CI
Negash 2013	-1.38	0.13		-1.38 [-1.63, -1.13]
Simoni 2002	-0.94	0.332		-0.94 [-1.59, -0.29]
Berhe 2013	-0.9	0.11		-0.90 [-1.12, -0.68]
Boarts 2006	-0.87	0.28		-0.87 [-1.42, -0.32]
Ammassari 2004	-0.84	0.195		-0.84 [-1.22, -0.46]
Gibbie 2007	-0.79	0.258		-0.79 [-1.30, -0.28]
Finnocchario 2011	-0.76	0.171		-0.76 [-1.10, -0.42]
Catz 2000	-0.75	0.257		-0.75 [-1.25, -0.25]
Woodward 2013	-0.74	0.18		-0.74 [-1.09, -0.39]
Reynolds 2002	-0.703	0.127		-0.70 [-0.95, -0.45]
Tedaldi 2012	-0.63	0.09		-0.63 [-0.81, -0.45]
Nel 2013	-0.62	0.21		-0.62 [-1.03, -0.21]
Cardarelli 2008	-0.61	0.209		-0.61 [-1.02, -0.20]
Nilsson 2006	-0.61	0.167		-0.61 [-0.94, -0.28]
Amberbir 2008	-0.6	0.12		-0.60 [-0.84, -0.36]
Kalichman 2010	-0.6	0.146		-0.60 [-0.89, -0.31]
Dilorio 2007	-0.59	0.13		-0.59 [-0.84, -0.34]
Paterson 2000	-0.56	0.28		-0.56 [-1.11, -0.01]
Webb 2009	-0.54	0.166		-0.54 [-0.87, -0.21]
Fatima 1 2013	-0.53	0.16		-0.53 [-0.84, -0.22]
Pratt 2001	-0.53	0.13		-0.53 [-0.78, -0.28]
Venkatesh 2010	-0.52	0.145		-0.52 [-0.80, -0.24]
Malow 2013	-0.47	0.15		-0.47 [-0.76, -0.18]
Rao 2012	-0.47	0.09		-0.47 [-0.65, -0.29]
Cha 2008	-0.47	0.14		-0.47 [-0.74, -0.20]
Ickovics 2002	-0.46	0.226		-0.46 [-0.90, -0.02]
Weidle 2006	-0.46	0.06		-0.46 [-0.58, -0.34]
Farley 2010	-0.39	0.143		-0.39 [-0.67, -0.11]
Mollassiotis 2002	-0.38	0.183		-0.38 [-0.74, -0.02]
Campos 2010	-0.38	0.122		-0.38 [-0.62, -0.14]
Etienne 2010	-0.37	0.07		-0.37 [-0.51, -0.23]
Sullivan 2007	-0.37	0.02		-0.37 [-0.41, -0.33]
Johnson 2003	-0.35	0.041		-0.35 [-0.43, -0.27]
Horne 2007	-0.35	0.183		-0.35 [-0.71, 0.01]
Lazo 1 2007	-0.35	0.081		-0.35 [-0.51, -0.19]
Bianco 1 2011	-0.34	0.162		-0.34 [-0.66, -0.02]
Kalichman 2003	-0.33	0.13		-0.33 [-0.58, -0.08]
Tadios 2006	-0.32	0.12		-0.32 [-0.56, -0.08]
Gordillo 1999	-0.32	0.101		-0.32 [-0.52, -0.12]
Servellen 2002	-0.32	0.142		-0.32 [-0.60, -0.04]
Feldman 2013	-0.32	0.04		-0.32 [-0.40, -0.24]
Ramadhani 2007	-0.31	0.22		-0.31 [-0.74, 0.12]
Holmes 2007	-0.3	0.19		-0.30 [-0.67, 0.07]
Sumari 2011	-0.28	0.141		-0.28 [-0.56, -0.00]
Simoni 2012	-0.28	0.04		-0.28 [-0.36, -0.20]
Anuradha 2012	-0.28	0.121		-0.28 [-0.52, -0.04]
Do 2010	-0.27	0.15		-0.27 [-0.56, 0.02]
Franke 2010	-0.26	0.46		-0.26 [-1.16, 0.64]
[illegible]	[illegible]	[illegible]		[illegible]

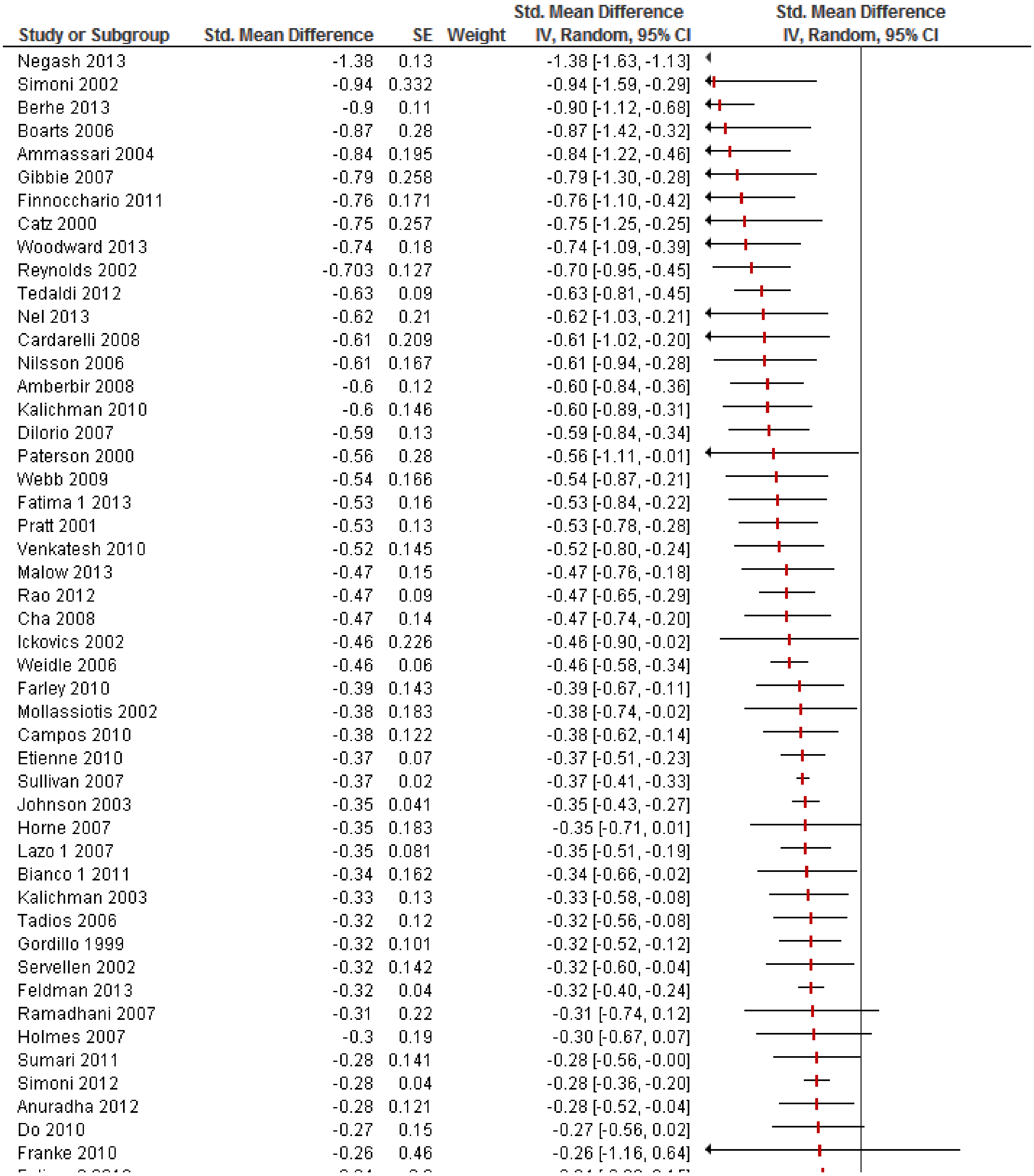

[illegible]	[illegible]	[illegible]	[illegible]
Fatima 2 2013	-0.24	0.2	-0.24 [-0.63, 0.15]
Sherr 2010	-0.24	0.101	-0.24 [-0.44, -0.04]
Juday 2011	-0.24	0.101	-0.24 [-0.44, -0.04]
Bottonari 2012	-0.22	0.141	-0.22 [-0.50, 0.06]
Raboud 2011	-0.22	0.1	-0.22 [-0.42, -0.02]
Leombruni 2009	-0.22	0.181	-0.22 [-0.57, 0.13]
Mugavero 2009	-0.22	0.121	-0.22 [-0.46, 0.02]
Holzemer 1999	-0.18	0.1	-0.18 [-0.38, 0.02]
Gonzalez 2004	-0.18	0.221	-0.18 [-0.61, 0.25]
Moralejo 2006	-0.17	0.161	-0.17 [-0.49, 0.15]
Andrade 2013	-0.16	0.22	-0.16 [-0.59, 0.27]
Murri 2001	-0.16	0.181	-0.16 [-0.51, 0.19]
Kyser 2011	-0.16	0.12	-0.16 [-0.40, 0.08]
Li 2010	-0.15	0.1	-0.15 [-0.35, 0.05]
Kumar 2009	-0.14	0.06	-0.14 [-0.26, -0.02]
Kleeberger 2001	-0.14	0.08	-0.14 [-0.30, 0.02]
Protopopescu 2009	-0.14	0.06	-0.14 [-0.26, -0.02]
Wagner 2011	-0.12	0.14	-0.12 [-0.39, 0.15]
Golin 2002	-0.1	0.18	-0.10 [-0.45, 0.25]
Stirratt 2006	-0.08	0.14	-0.08 [-0.35, 0.19]
Kalichman 2008	-0.08	0.19	-0.08 [-0.45, 0.29]
Leserman 2008	-0.04	0.2	-0.04 [-0.43, 0.35]
King 2012	-0.02	0.11	-0.02 [-0.24, 0.20]
Hanif 2013	-0.02	0.08	-0.02 [-0.18, 0.14]
Lazo 2 2007	-0.02	0.06	-0.02 [-0.14, 0.10]
Johnson 2012	-0.02	0.12	-0.02 [-0.26, 0.22]
Barclay 2 2007	0	0.3	0.00 [-0.59, 0.59]
Murphy 2004	0	0.18	0.00 [-0.35, 0.35]
Silva 2009	0	0.1	0.00 [-0.20, 0.20]
Oyugi 2007	0	0.2	0.00 [-0.39, 0.39]
Kerr 2012	0	0.12	0.00 [-0.24, 0.24]
Gonzalez 2007	0	0.12	0.00 [-0.24, 0.24]
Barclay 1 2007	0	0.18	0.00 [-0.35, 0.35]
Shuter 2008	0	0.26	0.00 [-0.51, 0.51]
Chesney 2000	0	0.24	0.00 [-0.47, 0.47]
Pefura 2013	0.001	0.07	0.00 [-0.14, 0.14]
Heckman 2004	0.01	0.12	0.01 [-0.23, 0.25]
Thrasher 2008	0.02	0.04	0.02 [-0.06, 0.10]
Poquette 2013	0.05	0.25	0.05 [-0.44, 0.54]
Gifford 2000	0.11	0.18	0.11 [-0.24, 0.46]
Bianco 2 2011	0.16	0.221	0.16 [-0.27, 0.59]
Uuskula 2012	0.21	0.161	0.21 [-0.11, 0.53]

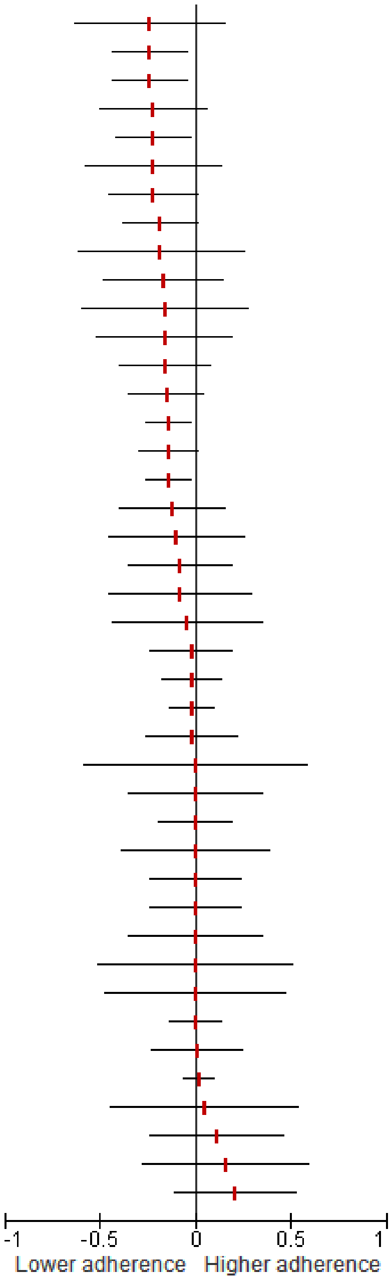

HIV stigma

Study or Subgroup	Std. Mean Difference	SE	Weight	Std. Mean Difference IV, Random, 95% CI
Fong 2003	-1.22	0.21		-1.22 [-1.63, -0.81]
Sellier 2006	-1.13	0.3		-1.13 [-1.72, -0.54]
Nagesh 2013	-1.01	0.13		-1.01 [-1.26, -0.76]
Mellins 2003	-0.84	0.37		-0.84 [-1.57, -0.11]
Fatima 2 2013	-0.74	0.2		-0.74 [-1.13, -0.35]
Sayles 2009	-0.72	0.18		-0.72 [-1.07, -0.37]
Sasaki 2012	-0.65	0.17		-0.65 [-0.98, -0.32]
Dlamini 2009	-0.46	0.06		-0.46 [-0.58, -0.34]
Nozaki 2011	-0.45	0.15		-0.45 [-0.74, -0.16]
Dilorio 2007	-0.45	0.13		-0.45 [-0.70, -0.20]
Wagner 2011	-0.43	0.15		-0.43 [-0.72, -0.14]
Rao 2012	-0.43	0.09		-0.43 [-0.61, -0.25]
Boyer 2011	-0.37	0.04		-0.37 [-0.45, -0.29]
Cardarelli 2008	-0.37	0.23		-0.37 [-0.82, 0.08]
Waite 2008	-0.37	0.14		-0.37 [-0.64, -0.10]
Lyimo 2014	-0.37	0.16		-0.37 [-0.68, -0.06]
Wasti 2012	-0.36	0.16		-0.36 [-0.67, -0.05]
Durante 2003	-0.36	0.27		-0.36 [-0.89, 0.17]
Sumari 2011	-0.33	0.15		-0.33 [-0.62, -0.04]
Stirratt 2006	-0.32	0.14		-0.32 [-0.59, -0.05]
Frain 2009	-0.3	0.3		-0.30 [-0.89, 0.29]
Kalichman 2010	-0.29	0.16		-0.29 [-0.60, 0.02]
Gokarn 2012	-0.28	0.14		-0.28 [-0.55, -0.01]
Do 2010	-0.27	0.15		-0.27 [-0.56, 0.02]
Holmes 2007	-0.23	0.19		-0.23 [-0.60, 0.14]
Peretti 3 2006	-0.19	0.11		-0.19 [-0.41, 0.03]
Peretti 2 2006	-0.14	0.09		-0.14 [-0.32, 0.04]
Spire 2002	-0.14	0.11		-0.14 [-0.36, 0.08]
Raboud 2011	-0.14	0.1		-0.14 [-0.34, 0.06]
Li 2010	-0.12	0.11		-0.12 [-0.34, 0.10]
Fatima 1 2013	-0.11	0.16		-0.11 [-0.42, 0.20]
Unge 2010	-0.1	0.18		-0.10 [-0.45, 0.25]
Rintamaki 2006	-0.1	0.15		-0.10 [-0.39, 0.19]
Birbeck 2011	-0.09	0.09		-0.09 [-0.27, 0.09]
Berhe 2013	-0.07	0.11		-0.07 [-0.29, 0.15]
Falang 2012	-0.06	0.14		-0.06 [-0.33, 0.21]
Peretti 1 2006	-0.05	0.08		-0.05 [-0.21, 0.11]
Li 2011	-0.03	0.14		-0.03 [-0.30, 0.24]
Rougemont 2009	-0.02	0.14		-0.02 [-0.29, 0.25]
Franke 2010	-0.02	0.46		-0.02 [-0.92, 0.88]
Wang 2007	0	0.19		0.00 [-0.37, 0.37]
Tadios 2006	0	0.12		0.00 [-0.24, 0.24]
Giday 2010	0	0.14		0.00 [-0.27, 0.27]
Kalichman 2008	0	0.19		0.00 [-0.37, 0.37]
Peltzer 2010	0.08	0.11		0.08 [-0.14, 0.30]
Sherr 2008	0.11	0.08		0.11 [-0.05, 0.27]
Bonolo 2005	0.27	0.12		0.27 [0.03, 0.51]

Std. Mean Difference
IV, Random, 95% CI

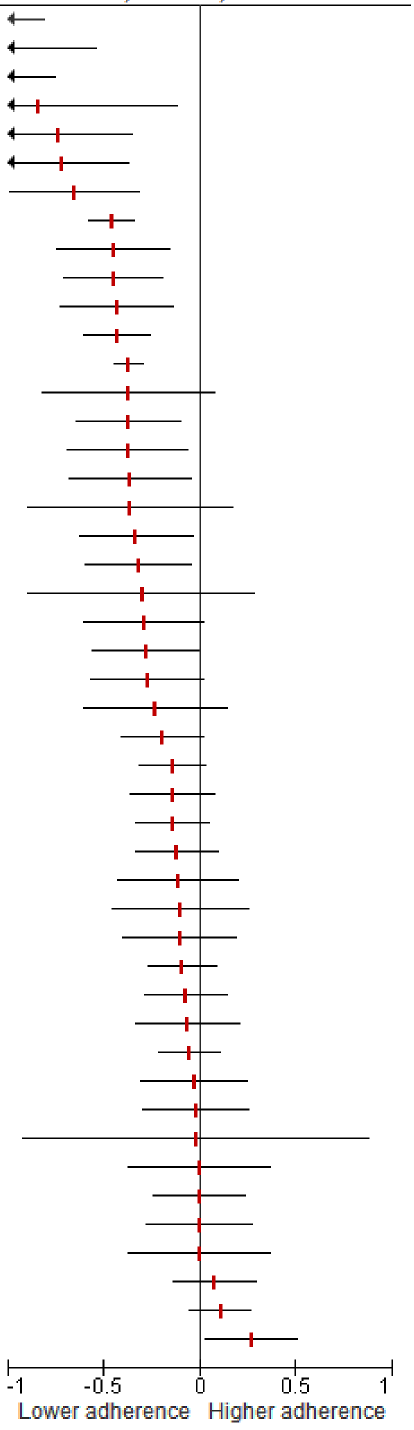

Social support

Study or Subgroup	Std. Mean Difference	SE	Weight	Std. Mean Difference IV, Random, 95% CI
Tiyou 2010	-0.41	0.122		-0.41 [-0.65, -0.17]
Garcia 2006	-0.28	0.141		-0.28 [-0.56, -0.00]
Nilsson 2006	-0.27	0.162		-0.27 [-0.59, 0.05]
Unge 2010	-0.27	0.101		-0.27 [-0.47, -0.07]
Berhe 2013	-0.14	0.11		-0.14 [-0.36, 0.08]
Mollassiotis 2002	-0.09	0.18		-0.09 [-0.44, 0.26]
Holmes 2007	-0.08	0.19		-0.08 [-0.45, 0.29]
Kacanek 2010	-0.08	0.15		-0.08 [-0.37, 0.21]
Kalichman 2008	-0.07	0.19		-0.07 [-0.44, 0.30]
Heckman 2004	-0.07	0.12		-0.07 [-0.31, 0.17]
Durante 2003	-0.02	0.26		-0.02 [-0.53, 0.49]
Thrasher 2008	0	0.04		0.00 [-0.08, 0.08]
Remien 2007	0	0.2		0.00 [-0.39, 0.39]
Holzemer 1999	0	0.1		0.00 [-0.20, 0.20]
Sherr 2008	0	0.08		0.00 [-0.16, 0.16]
Chesney 2000	0	0.24		0.00 [-0.47, 0.47]
Boyer 2011	0.01	0.04		0.01 [-0.07, 0.09]
Giday 2010	0.02	0.14		0.02 [-0.25, 0.29]
Li 2010	0.03	0.1		0.03 [-0.17, 0.23]
Kumar 2009	0.03	0.06		0.03 [-0.09, 0.15]
Weaver 2005	0.06	0.12		0.06 [-0.18, 0.30]
Reynolds 2004	0.06	0.12		0.06 [-0.18, 0.30]
Sodergard 2006	0.07	0.07		0.07 [-0.07, 0.21]
Franke 2010	0.07	0.46		0.07 [-0.83, 0.97]
Glass 2010	0.09	0.02		0.09 [0.05, 0.13]
Power 2003	0.09	0.27		0.09 [-0.44, 0.62]
Protopopescu 2009	0.12	0.06		0.12 [0.00, 0.24]
Gonzalez 2004	0.14	0.221		0.14 [-0.29, 0.57]
Anuradha 2012	0.14	0.12		0.14 [-0.10, 0.38]
Cha 2008	0.16	0.14		0.16 [-0.11, 0.43]
Peltzer 2010	0.18	0.12		0.18 [-0.06, 0.42]
Kalichman 2003	0.2	0.13		0.20 [-0.05, 0.45]
Golin 2002	0.22	0.181		0.22 [-0.13, 0.57]
Johnson 2003	0.22	0.04		0.22 [0.14, 0.30]
Simoni 2002	0.24	0.302		0.24 [-0.35, 0.83]
Bianco 2 2011	0.26	0.222		0.26 [-0.18, 0.70]
Servellen 2002	0.28	0.141		0.28 [0.00, 0.56]
Pratt 2001	0.28	0.12		0.28 [0.04, 0.52]
Godin 2005	0.3	0.101		0.30 [0.10, 0.50]
Hanif 2013	0.3	0.08		0.30 [0.14, 0.46]
Woodward 2013	0.31	0.17		0.31 [-0.02, 0.64]
Nozaki 2011	0.31	0.15		0.31 [0.02, 0.60]
Dilorio 2007	0.31	0.13		0.31 [0.06, 0.56]
Duong 2001	0.32	0.27		0.32 [-0.21, 0.85]
Vyavaharkar 2007	0.32	0.142		0.32 [0.04, 0.60]
Raboud 2011	0.33	0.1		0.33 [0.13, 0.53]
Ferguson 2002	0.34	0.162		0.34 [0.02, 0.66]
Spire 2002	0.36	0.102		0.36 [0.16, 0.56]
Huynh 2013	0.37	17		0.37 [-32.95, 33.69]
Shah 2007	0.37	0.14		0.37 [0.10, 0.64]
Gordillo 1999	0.39	0.102		0.39 [0.19, 0.59]
Barclay 2007	0.39	0.143		0.39 [0.11, 0.67]
Diabate 2007	0.4	0.09		0.40 [0.22, 0.58]
Bianco 1 2011	0.41	0.163		0.41 [0.09, 0.73]
Amberbir 2008	0.47	0.12		0.47 [0.23, 0.71]
Kalichman 1999	0.51	0.144		0.51 [0.23, 0.79]
Gifford 2000	0.51	0.186		0.51 [0.15, 0.87]
Singh 1999	0.52	0.24		0.52 [0.05, 0.99]
Gibbie 2007	0.57	0.249		0.57 [0.08, 1.06]
Murphy 2004	0.57	0.187		0.57 [0.20, 0.94]
Adewuya 2010	0.63	0.17		0.63 [0.30, 0.96]
Nakimulli 2013	0.72	0.1		0.72 [0.52, 0.92]
Ladefoged 2012	0.75	0.32		0.75 [0.12, 1.38]
Luszczynska 2007	0.87	0.218		0.87 [0.44, 1.30]
Cardarelli 2008	0.99	0.223		0.99 [0.55, 1.43]
Catz 2000	1	0.268		1.00 [0.47, 1.53]
Finnocchario 2011	1.5	0.201		1.50 [1.11, 1.89]

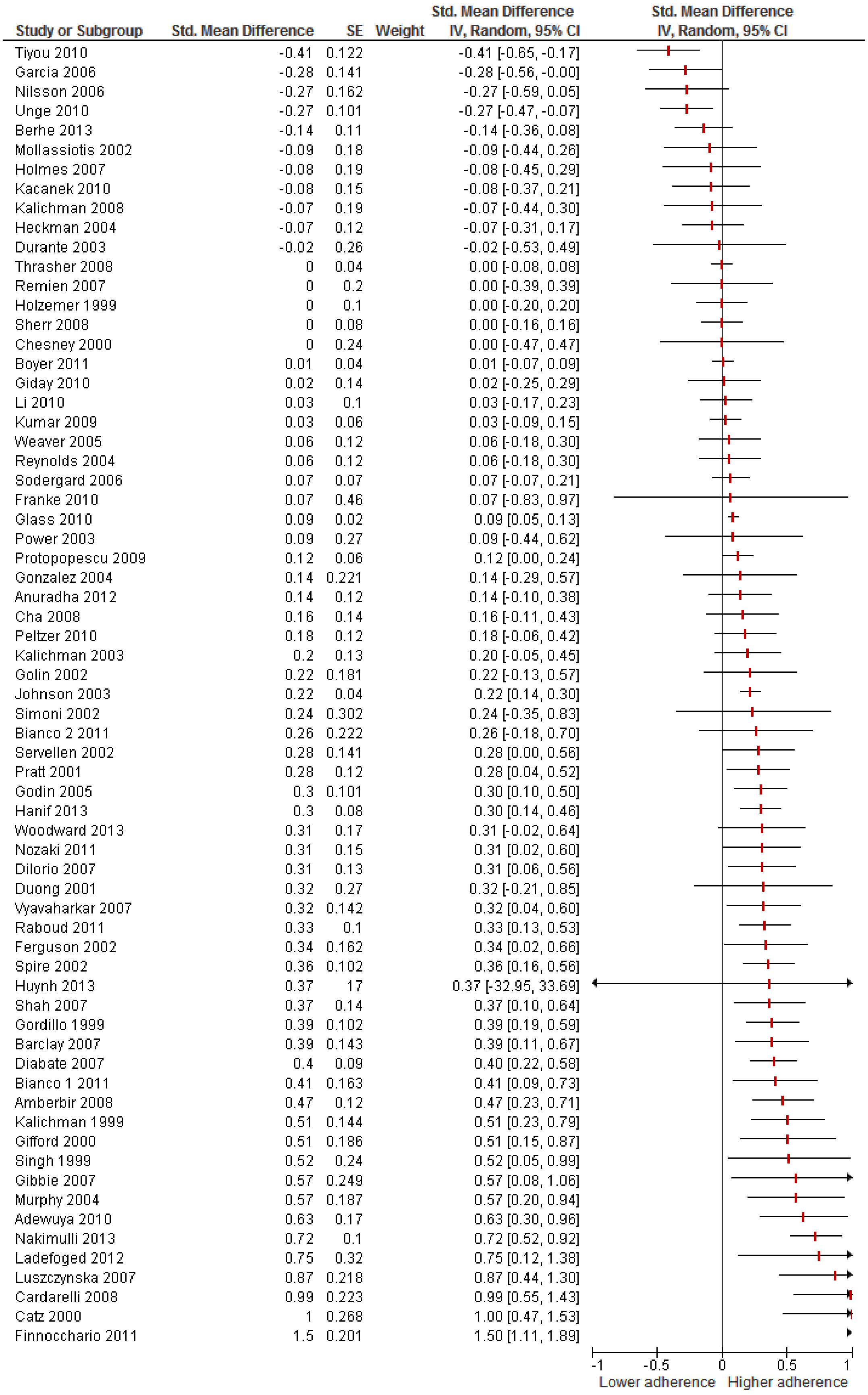

PI containing regimen

Study or Subgroup	Std. Mean Difference	SE	Weight	Std. Mean Difference IV, Random, 95% CI
Seguy 2 2007	-0.83	0.11		-0.83 [-1.05, -0.61]
Cahn 2004	-0.63	0.12		-0.63 [-0.87, -0.39]
Nelson 2013	-0.56	0.09		-0.56 [-0.74, -0.38]
Fong 2003	-0.56	0.2		-0.56 [-0.95, -0.17]
Lazo 1 2007	-0.37	0.081		-0.37 [-0.53, -0.21]
Howard 2002	-0.33	0.162		-0.33 [-0.65, -0.01]
Finocchario 2011	-0.32	0.162		-0.32 [-0.64, -0.00]
Trotta 2003	-0.28	0.08		-0.28 [-0.44, -0.12]
OConnor 2013	-0.27	0.03		-0.27 [-0.33, -0.21]
Sumari 2011	-0.26	0.141		-0.26 [-0.54, 0.02]
Lazo 2 2007	-0.23	0.06		-0.23 [-0.35, -0.11]
Raboud 2011	-0.2	0.1		-0.20 [-0.40, -0.00]
Gay 2011	-0.14	0.12		-0.14 [-0.38, 0.10]
Sullivan 2007	-0.14	0.02		-0.14 [-0.18, -0.10]
Kyser 2011	-0.12	0.12		-0.12 [-0.36, 0.12]
Mannheimer 2002	-0.12	0.06		-0.12 [-0.24, -0.00]
Protopopescu 2009	-0.12	0.06		-0.12 [-0.24, -0.00]
Etard 2007	-0.09	0.19		-0.09 [-0.46, 0.28]
Glass 2010	-0.03	0.04		-0.03 [-0.11, 0.05]
Wagner 2002	0	0.26		0.00 [-0.51, 0.51]
Orrell 2003	0	0.12		0.00 [-0.24, 0.24]
Eholie 2007	0	0.13		0.00 [-0.25, 0.25]
Nilsson 2006	0	0.16		0.00 [-0.31, 0.31]
Mollassiotis 2002	0	0.18		0.00 [-0.35, 0.35]
Parruti 2006	0.23	0.161		0.23 [-0.09, 0.55]
Seguy 1 2007	0.38	0.14		0.38 [0.11, 0.65]

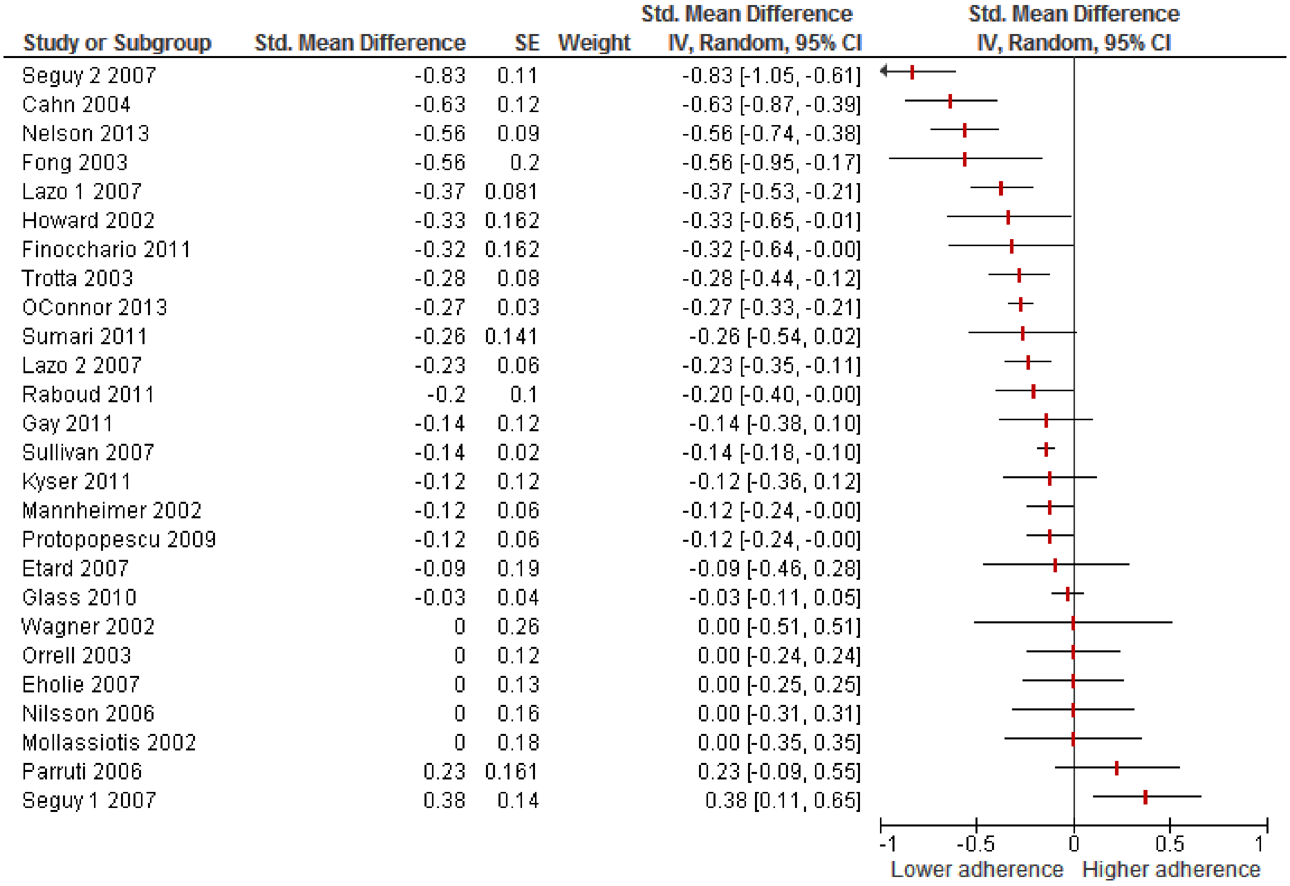

Daily dosing frequency

Study or Subgroup	Std. Mean Difference	SE	Weight	Std. Mean Difference IV, Random, 95% CI
Busher 2012	-0.84	0.21		-0.84 [-1.25, -0.43]
Falang 2012	-0.62	0.105		-0.62 [-0.83, -0.41]
Orrell 2003	-0.58	0.12		-0.58 [-0.82, -0.34]
Golin 2002	-0.51	0.186		-0.51 [-0.87, -0.15]
Cahn 2004	-0.43	0.123		-0.43 [-0.67, -0.19]
Howard 2002	-0.32	0.162		-0.32 [-0.64, -0.00]
Pinheiro 2002	-0.31	0.142		-0.31 [-0.59, -0.03]
Blackstock 2012	-0.27	0.162		-0.27 [-0.59, 0.05]
Raboud 2011	-0.27	0.1		-0.27 [-0.47, -0.07]
Maggiolo 2002	-0.25	0.08		-0.25 [-0.41, -0.09]
Glass 2010	-0.2	0.02		-0.20 [-0.24, -0.16]
Protopopescu 2009	-0.2	0.06		-0.20 [-0.32, -0.08]
Aragones 2011	-0.19	0.08		-0.19 [-0.35, -0.03]
Uuskula 2012	-0.19	0.161		-0.19 [-0.51, 0.13]
OConnor 2013	-0.19	0.03		-0.19 [-0.25, -0.13]
Fong 2003	-0.17	0.2		-0.17 [-0.56, 0.22]
Kyser 2011	-0.06	0.12		-0.06 [-0.30, 0.18]
Sodergard 2006	-0.05	0.07		-0.05 [-0.19, 0.09]
Pratt 2001	-0.05	0.12		-0.05 [-0.29, 0.19]
Johnson 2003	-0.03	0.04		-0.03 [-0.11, 0.05]
Cooper 2010	-0.02	0.21		-0.02 [-0.43, 0.39]
Gianotti 2012	-0.01	0.06		-0.01 [-0.13, 0.11]
Finocchario 2011	0	0.16		0.00 [-0.31, 0.31]
Wagner 2002	0	0.26		0.00 [-0.51, 0.51]
Nilsson 2006	0	0.16		0.00 [-0.31, 0.31]
Gifford 2000	0	0.18		0.00 [-0.35, 0.35]
Rougemont 2009	0.06	0.14		0.06 [-0.21, 0.33]
Mollassiotis 2002	0.1	0.18		0.10 [-0.25, 0.45]
Gao 2000	0.12	0.24		0.12 [-0.35, 0.59]

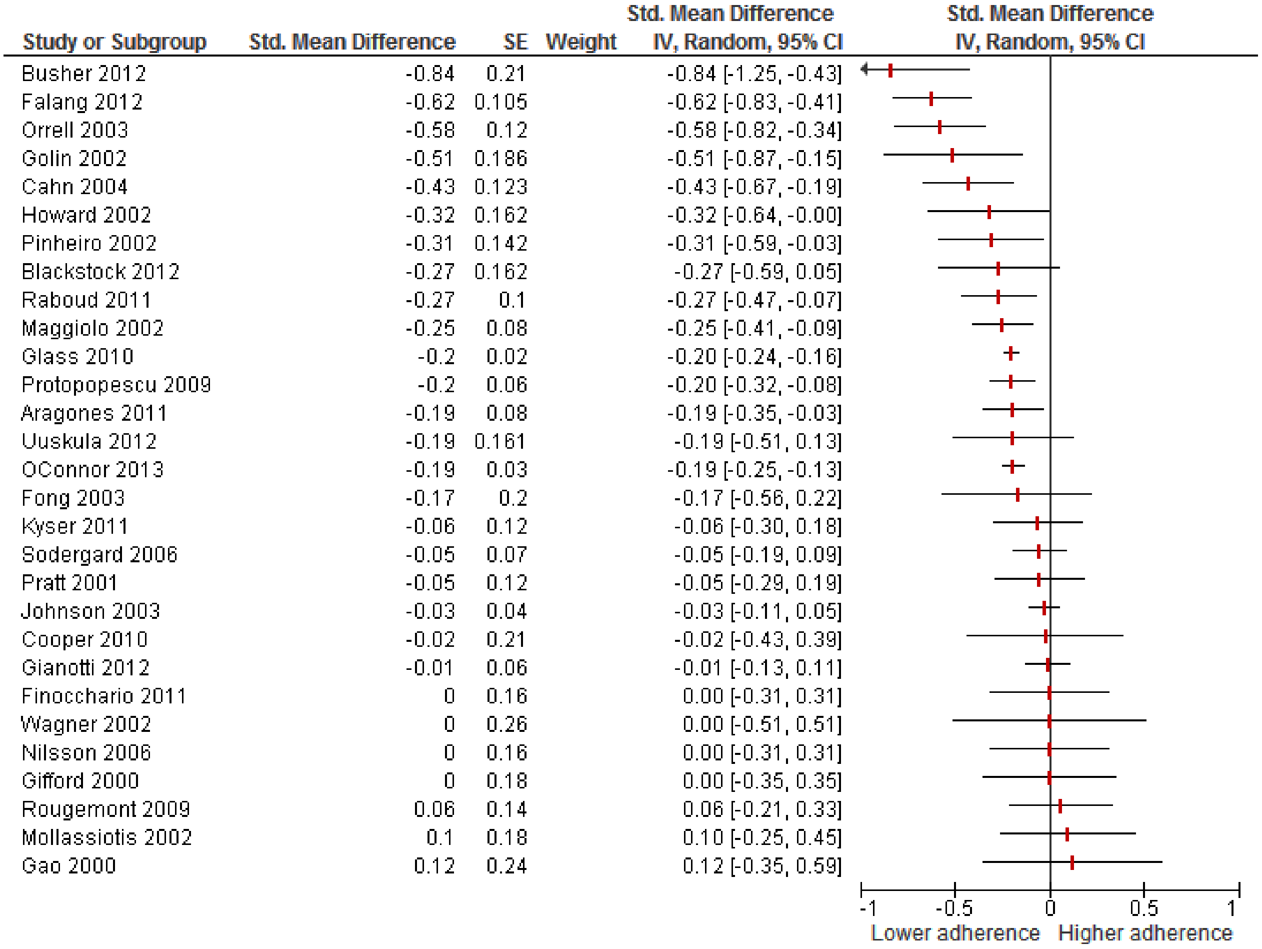

Financial constraints

Study or Subgroup	Std. Mean Difference	SE	Weight	Std. Mean Difference IV, Random, 95% CI
Alakija 2010	-1.47	0.149		-1.47 [-1.76, -1.18]
McAllister 2013	-0.98	0.12		-0.98 [-1.22, -0.74]
Ladefoged 2012	-0.95	0.33		-0.95 [-1.60, -0.30]
Hanif 2013	-0.75	0.08		-0.75 [-0.91, -0.59]
Giday 2010	-0.7	0.14		-0.70 [-0.97, -0.43]
Wasti 2012	-0.68	0.127		-0.68 [-0.93, -0.43]
Unge 2010	-0.64	0.105		-0.64 [-0.85, -0.43]
Barclay 2 2007	-0.62	0.315		-0.62 [-1.24, -0.00]
Ickovics 2002	-0.59	0.229		-0.59 [-1.04, -0.14]
Arrivillaga 2009	-0.58	0.13		-0.58 [-0.83, -0.33]
Paterson 2000	-0.58	0.28		-0.58 [-1.13, -0.03]
Kyser 2011	-0.53	0.12		-0.53 [-0.77, -0.29]
Blackstock 2012	-0.52	0.165		-0.52 [-0.84, -0.20]
Howard 2002	-0.51	0.165		-0.51 [-0.83, -0.19]
Yun 2005	-0.48	0.103		-0.48 [-0.68, -0.28]
Tran 2013	-0.47	0.07		-0.47 [-0.61, -0.33]
Duggan 2009	-0.46	0.185		-0.46 [-0.82, -0.10]
Holmes 2007	-0.45	0.19		-0.45 [-0.82, -0.08]
Lazo 1 2007	-0.44	0.082		-0.44 [-0.60, -0.28]
Ammassari 2004	-0.43	0.184		-0.43 [-0.79, -0.07]
Campos 2010	-0.41	0.122		-0.41 [-0.65, -0.17]
Carballo 2004	-0.38	0.13		-0.38 [-0.63, -0.13]
Oku 2014	-0.38	0.1		-0.38 [-0.58, -0.18]
Berhe 2013	-0.37	0.11		-0.37 [-0.59, -0.15]
Graham 2007	-0.36	0.22		-0.36 [-0.79, 0.07]
Golin 2002	-0.35	0.183		-0.35 [-0.71, 0.01]
Kleeberger 2001	-0.34	0.1		-0.34 [-0.54, -0.14]
Bonolo 2005	-0.33	0.12		-0.33 [-0.57, -0.09]
Barclay 1 2007	-0.33	0.182		-0.33 [-0.69, 0.03]
Kacanek 2010	-0.32	0.15		-0.32 [-0.61, -0.03]
Holstad 2006	-0.32	0.182		-0.32 [-0.68, 0.04]
Spire 2002	-0.32	0.101		-0.32 [-0.52, -0.12]
Colbert 2013	-0.27	0.19		-0.27 [-0.64, 0.10]
Peretti 2 2006	-0.27	0.081		-0.27 [-0.43, -0.11]
Singh 1999	-0.25	0.24		-0.25 [-0.72, 0.22]
Nozaki 2011	-0.25	0.15		-0.25 [-0.54, 0.04]
Fatima 1 2013	-0.25	0.16		-0.25 [-0.56, 0.06]
Nakimulli 2013	-0.25	0.1		-0.25 [-0.45, -0.05]
Birbeck 2011	-0.24	0.101		-0.24 [-0.44, -0.04]
Fatima 2 2013	-0.24	0.2		-0.24 [-0.63, 0.15]
Brown 2013	-0.23	0.19		-0.23 [-0.60, 0.14]
Peretti 3 2006	-0.22	0.101		-0.22 [-0.42, -0.02]
Finnocchario 2011	-0.22	0.161		-0.22 [-0.54, 0.10]
Adewuya 2010	-0.21	0.17		-0.21 [-0.54, 0.12]
Falang 2012	-0.2	0.101		-0.20 [-0.40, -0.00]
Gokarn 2012	-0.19	0.14		-0.19 [-0.46, 0.08]
Bottonari 2012	-0.19	0.141		-0.19 [-0.47, 0.09]
Boyer 2011	-0.18	0.04		-0.18 [-0.26, -0.10]
Silva 2009	-0.17	0.1		-0.17 [-0.37, 0.03]
Trasher 2008	-0.16	0.05		-0.16 [-0.26, -0.06]
Cauldbeck 2009	-0.16	0.281		-0.16 [-0.71, 0.39]
Uuskula 2012	-0.16	0.161		-0.16 [-0.48, 0.16]
Pinheiro 2002	-0.16	0.14		-0.16 [-0.43, 0.11]
Simoni 2012	-0.16	0.04		-0.16 [-0.24, -0.08]
Peretti 1 2006	-0.15	0.08		-0.15 [-0.31, 0.01]
Raboud 2011	-0.15	0.1		-0.15 [-0.35, 0.05]
Wagner 2011	-0.14	0.14		-0.14 [-0.41, 0.13]
Parruti 2006	-0.14	0.16		-0.14 [-0.45, 0.17]
Franke 2010	-0.14	0.46		-0.14 [-1.04, 0.76]

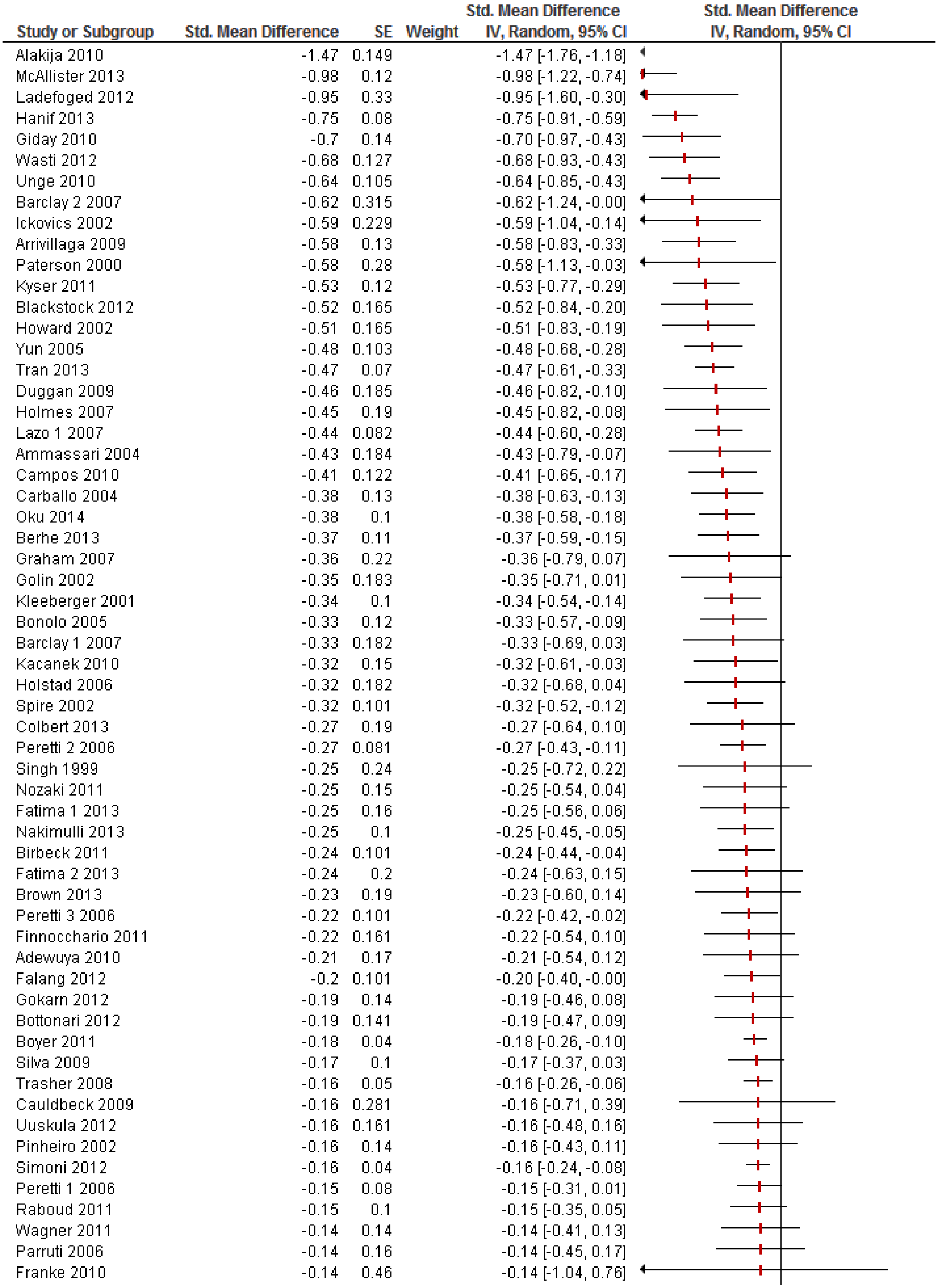

Johnson 2003	-0.13	0.04	-0.13 [-0.21, -0.05]
Tiyou 2010	-0.12	0.12	-0.12 [-0.36, 0.12]
Gibbie 2007	-0.1	0.24	-0.10 [-0.57, 0.37]
Leserman 2008	-0.09	0.2	-0.09 [-0.48, 0.30]
Lazo 2 2007	-0.08	0.06	-0.08 [-0.20, 0.04]
Weaver 2005	-0.08	0.12	-0.08 [-0.32, 0.16]
Gao 2000	-0.08	0.24	-0.08 [-0.55, 0.39]
Ukwe 2010	-0.07	0.12	-0.07 [-0.31, 0.17]
Protopopescu 2009	-0.06	0.06	-0.06 [-0.18, 0.06]
Frain 2009	-0.06	0.24	-0.06 [-0.53, 0.41]
Garcia 2006	-0.06	0.14	-0.06 [-0.33, 0.21]
Huynh 2013	-0.06	0.17	-0.06 [-0.39, 0.27]
Li 2010	-0.05	0.1	-0.05 [-0.25, 0.15]
Dilorio 2007	-0.02	0.22	-0.02 [-0.45, 0.41]
Gay 2011	-0.01	0.12	-0.01 [-0.25, 0.23]
Sayles 2009	-0.01	0.17	-0.01 [-0.34, 0.32]
Juday 2011	-0.01	0.1	-0.01 [-0.21, 0.19]
Kamau 2011	0	0.1	0.00 [-0.20, 0.20]
Anuradha 2012	0	0.12	0.00 [-0.24, 0.24]
Oyugi 2007	0	0.2	0.00 [-0.39, 0.39]
Wang 2007	0	0.19	0.00 [-0.37, 0.37]
Gonzalez 2004	0	0.22	0.00 [-0.43, 0.43]
Bianco 2011	0	0.12	0.00 [-0.24, 0.24]
Eholie 2007	0	0.13	0.00 [-0.25, 0.25]
Tadios 2006	0	0.12	0.00 [-0.24, 0.24]
Safren 2005	0	0.13	0.00 [-0.25, 0.25]
Do 2010	0	0.15	0.00 [-0.29, 0.29]
Holzemer 1999	0	0.1	0.00 [-0.20, 0.20]
Remien 2007	0	0.2	0.00 [-0.39, 0.39]
Wagner 2002	0	0.26	0.00 [-0.51, 0.51]
Heckman 2004	0	0.12	0.00 [-0.24, 0.24]
Vyavaharkar 2007	0	0.14	0.00 [-0.27, 0.27]
Gonzalez 2007	0	0.12	0.00 [-0.24, 0.24]
Orrell 2003	0	0.12	0.00 [-0.24, 0.24]
Peltzer 2010	0	0.11	0.00 [-0.22, 0.22]
Chesney 2000	0	0.24	0.00 [-0.47, 0.47]
Kerr 2012	0	0.12	0.00 [-0.24, 0.24]
Catz 2000	0	0.24	0.00 [-0.47, 0.47]
Rodrigues 2012	0.001	0.24	0.00 [-0.47, 0.47]
Kalichman 2010	0.01	0.14	0.01 [-0.26, 0.28]
Dortz 2003	0.01	0.3	0.01 [-0.58, 0.60]
Servellen 2002	0.04	0.14	0.04 [-0.23, 0.31]
Sherr 2010	0.05	0.1	0.05 [-0.15, 0.25]
Kalichman 1999	0.06	0.14	0.06 [-0.21, 0.33]
Busher 2012	0.14	0.2	0.14 [-0.25, 0.53]
Shah 2007	0.18	0.14	0.18 [-0.09, 0.45]
Kumar 2009	0.2	0.06	0.20 [0.08, 0.32]
Brigido 2001	0.23	0.342	0.23 [-0.44, 0.90]
Elul 2013	0.27	0.06	0.27 [0.15, 0.39]
Venkatesh 2010	0.33	0.142	0.33 [0.05, 0.61]
Sellier 2006	1.64	0.32	1.64 [1.01, 2.27]

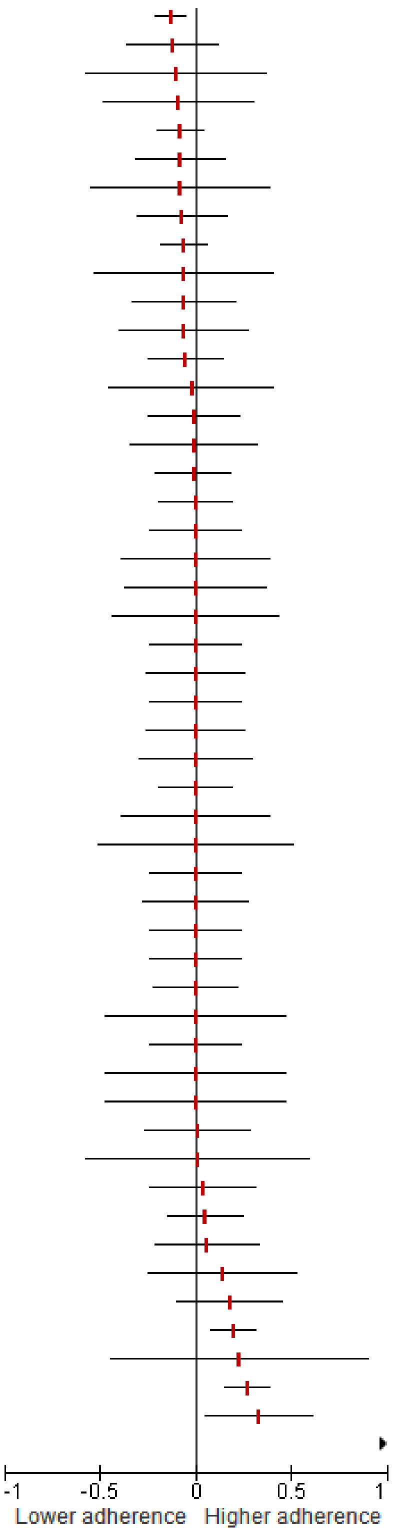

Pill burden

Study or Subgroup	Std. Mean Difference	SE	Weight	Std. Mean Difference IV, Random, 95% CI
Carmody 2003	-1.07	0.36		-1.07 [-1.78, -0.36]
Cauldbeck 2009	-0.76	0.3		-0.76 [-1.35, -0.17]
Kleeberger 2001	-0.54	0.083		-0.54 [-0.70, -0.38]
Brown 2013	-0.53	0.19		-0.53 [-0.90, -0.16]
Carballo 2004	-0.45	0.15		-0.45 [-0.74, -0.16]
Cahn 2004	-0.44	0.123		-0.44 [-0.68, -0.20]
Bonolo 2005	-0.41	0.12		-0.41 [-0.65, -0.17]
Wasti 2012	-0.4	0.122		-0.40 [-0.64, -0.16]
Sullivan 2007	-0.37	0.02		-0.37 [-0.41, -0.33]
Juday 2011	-0.35	0.122		-0.35 [-0.59, -0.11]
Gonzalez 2007	-0.34	0.182		-0.34 [-0.70, 0.02]
Diabate 2007	-0.3	0.09		-0.30 [-0.48, -0.12]
Horne 2007	-0.28	0.182		-0.28 [-0.64, 0.08]
Fatima 1 2013	-0.24	0.16		-0.24 [-0.55, 0.07]
Aragones 2011	-0.22	0.08		-0.22 [-0.38, -0.06]
Busher 2012	-0.22	0.2		-0.22 [-0.61, 0.17]
Fong 2003	-0.21	0.2		-0.21 [-0.60, 0.18]
Silva 2009	-0.2	0.1		-0.20 [-0.40, -0.00]
Maggiolo 2002	-0.19	0.08		-0.19 [-0.35, -0.03]
Parutti 2006	-0.18	0.161		-0.18 [-0.50, 0.14]
Weaver 2005	-0.16	0.12		-0.16 [-0.40, 0.08]
Nakimulli 2013	-0.16	0.1		-0.16 [-0.36, 0.04]
Woods 2009	-0.14	0.221		-0.14 [-0.57, 0.29]
Lazo 2 2007	-0.12	0.06		-0.12 [-0.24, -0.00]
Fatima 2 2013	-0.11	0.19		-0.11 [-0.48, 0.26]
Protopopescu 2009	-0.1	0.06		-0.10 [-0.22, 0.02]
Lazo 1 2007	-0.1	0.08		-0.10 [-0.26, 0.06]
Pratt 2001	-0.09	0.12		-0.09 [-0.33, 0.15]
Sellier 2006	-0.07	0.28		-0.07 [-0.62, 0.48]
Gianotti 2012	-0.06	0.06		-0.06 [-0.18, 0.06]
Boyer 2011	-0.04	0.04		-0.04 [-0.12, 0.04]
Gay 2011	-0.04	0.12		-0.04 [-0.28, 0.20]
Johnson 2003	-0.03	0.04		-0.03 [-0.11, 0.05]
OConnor 2013	-0.02	0.03		-0.02 [-0.08, 0.04]
Kerr 2012	0	0.12		0.00 [-0.24, 0.24]
Gifford 2000	0	0.18		0.00 [-0.35, 0.35]
Mellins 2003	0	0.26		0.00 [-0.51, 0.51]
Nilsson 2006	0	0.16		0.00 [-0.31, 0.31]
Do 2010	0	0.15		0.00 [-0.29, 0.29]
Mannheimer 2002	0	0.06		0.00 [-0.12, 0.12]
Wagner 2002	0	0.26		0.00 [-0.51, 0.51]
Gauchet 2007	0	0.18		0.00 [-0.35, 0.35]
Catz 2000	0	0.24		0.00 [-0.47, 0.47]
Cardarelli 2008	0	0.2		0.00 [-0.39, 0.39]
Gonzalez 2004	0	0.22		0.00 [-0.43, 0.43]
Campbell 2010	0	0.31		0.00 [-0.61, 0.61]
Stirratt 2006	0.04	0.14		0.04 [-0.23, 0.31]
Uuskula 2012	0.05	0.16		0.05 [-0.26, 0.36]
Golin 2002	0.06	0.18		0.06 [-0.29, 0.41]
Poquette 2013	0.07	0.25		0.07 [-0.42, 0.56]
Rintamaki 2006	0.08	0.14		0.08 [-0.19, 0.35]
Seguy 2 2007	0.09	0.1		0.09 [-0.11, 0.29]
Thrasher 2008	0.1	0.04		0.10 [0.02, 0.18]
Falang 2012	0.14	0.1		0.14 [-0.06, 0.34]
Seguy 1 2007	0.19	0.14		0.19 [-0.08, 0.46]
Shah 2007	0.3	0.14		0.30 [0.03, 0.57]
Colbert 2013	0.45	0.19		0.45 [0.08, 0.82]

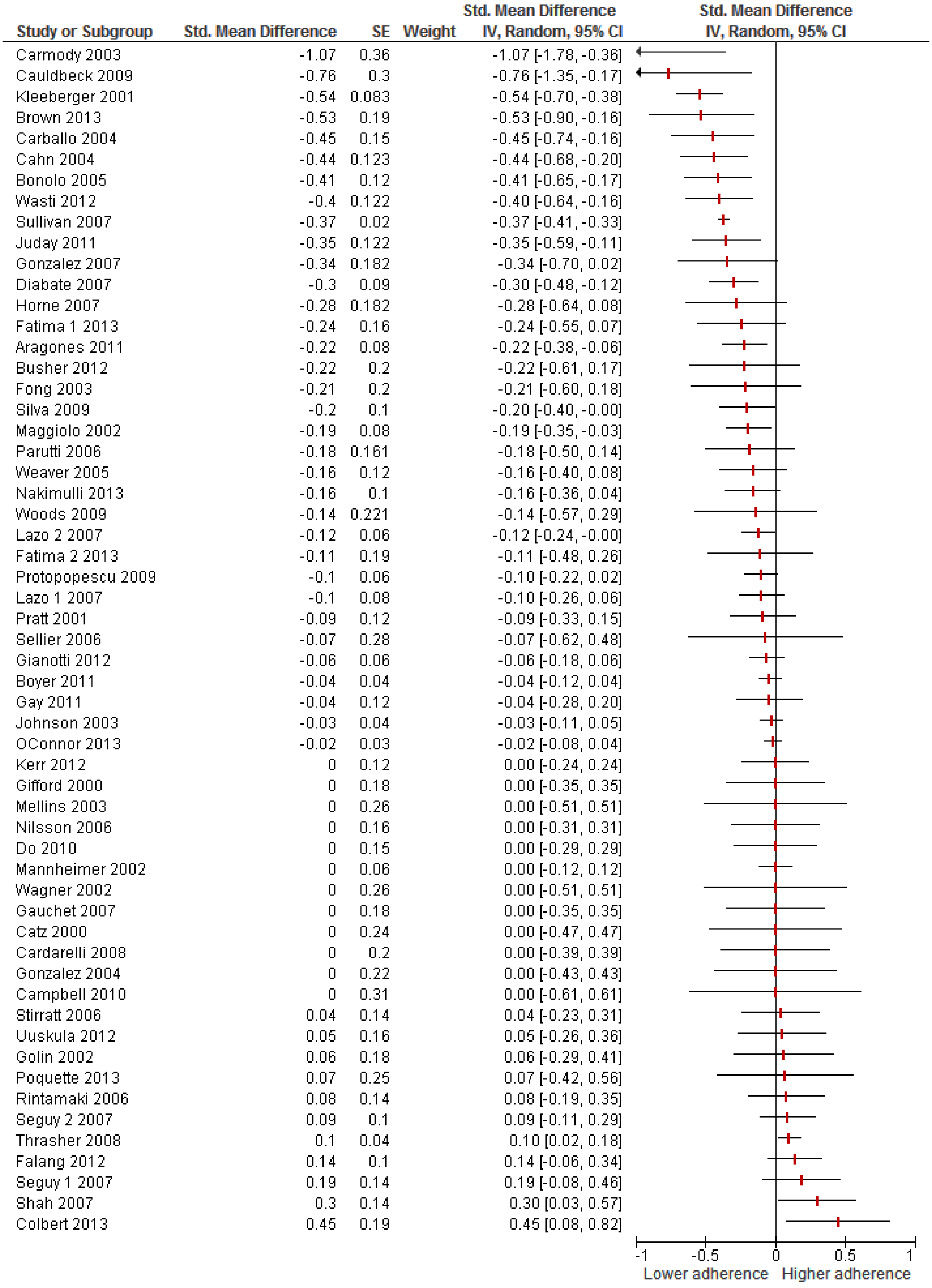

Age

Study or Subgroup	Std. Mean Difference	SE	Weight	Std. Mean Difference IV, Random, 95% CI
Ladefoged 2012	-1.04	0.33		-1.04 [-1.69, -0.39]
Nozaki 2011	-0.5	0.15		-0.50 [-0.79, -0.21]
Alakija 2010	-0.46	0.123		-0.46 [-0.70, -0.22]
Wasti 2012	-0.4	0.122		-0.40 [-0.64, -0.16]
Dortz 2003	-0.38	0.3		-0.38 [-0.97, 0.21]
McAllister 2013	-0.22	0.11		-0.22 [-0.44, -0.00]
Plankey 2009	-0.17	0.04		-0.17 [-0.25, -0.09]
Brigido 2001	-0.16	0.14		-0.16 [-0.43, 0.11]
Sasaki 2012	-0.16	0.16		-0.16 [-0.47, 0.15]
Yun 2005	-0.15	0.08		-0.15 [-0.31, 0.01]
Weidle 2006	-0.12	0.06		-0.12 [-0.24, -0.00]
Seguy 1 2007	-0.11	0.14		-0.11 [-0.38, 0.16]
Seguy 2 2007	-0.11	0.1		-0.11 [-0.31, 0.09]
Nelsen 2013	-0.11	0.14		-0.11 [-0.38, 0.16]
Mollassiotis 2002	-0.09	0.18		-0.09 [-0.44, 0.26]
Kacanek 2010	-0.08	0.15		-0.08 [-0.37, 0.21]
Campos 2010	-0.08	0.12		-0.08 [-0.32, 0.16]
Glass 2010	-0.07	0.02		-0.07 [-0.11, -0.03]
Singh 1999	-0.06	0.24		-0.06 [-0.53, 0.41]
Brown 2013	-0.05	0.19		-0.05 [-0.42, 0.32]
Ammassari 2004	-0.04	0.18		-0.04 [-0.39, 0.31]
Gao 2000	-0.02	0.24		-0.02 [-0.49, 0.45]
Orell 2003	-0.02	0.12		-0.02 [-0.26, 0.22]
King 2012	-0.02	0.11		-0.02 [-0.24, 0.20]
Protopopescu 2009	-0.02	0.06		-0.02 [-0.14, 0.10]
Maqutu 2011	-0.01	0.08		-0.01 [-0.17, 0.15]
Peltzer 2010	-0.01	0.11		-0.01 [-0.23, 0.21]
Gianotti 2012	-0.01	0.06		-0.01 [-0.13, 0.11]
Rodriques 2012	-0.01	0.24		-0.01 [-0.48, 0.46]
Garcia 2006	0	0.14		0.00 [-0.27, 0.27]
Safren 2005	0	0.13		0.00 [-0.25, 0.25]
Eholie 2007	0	0.13		0.00 [-0.25, 0.25]
Silva 2009	0	0.1		0.00 [-0.20, 0.20]
Oyugi 2007	0	0.2		0.00 [-0.39, 0.39]
Kamau 2011	0	0.1		0.00 [-0.20, 0.20]
Ukwe 2010	0	0.12		0.00 [-0.24, 0.24]
Duong 2001	0	0.26		0.00 [-0.51, 0.51]
Remien 2007	0	0.2		0.00 [-0.39, 0.39]
Ramadhani 2007	0	0.22		0.00 [-0.43, 0.43]
Do 2010	0	0.15		0.00 [-0.29, 0.29]
Tadios 2006	0	0.12		0.00 [-0.24, 0.24]
Power 2003	0	0.27		0.00 [-0.53, 0.53]
Giday 2010	0	0.14		0.00 [-0.27, 0.27]
Wang 2007	0	0.19		0.00 [-0.37, 0.37]
Simoni 2002	0	0.3		0.00 [-0.59, 0.59]
Ingersoll 2004	0	0.18		0.00 [-0.35, 0.35]
Nilsson 2006	0	0.16		0.00 [-0.31, 0.31]
Vyavaharkar 2007	0	0.14		0.00 [-0.27, 0.27]
Gifford 2000	0	0.18		0.00 [-0.35, 0.35]
Gay 2011	0	0.12		0.00 [-0.24, 0.24]
Holzemer 1999	0	0.1		0.00 [-0.20, 0.20]
Pinheiro 2002	0	0.14		0.00 [-0.27, 0.27]
Godin 2005	0	0.1		0.00 [-0.20, 0.20]
Kerr 2012	0	0.12		0.00 [-0.24, 0.24]
Haubrich 1999	0	0.16		0.00 [-0.31, 0.31]
Catz 2000	0	0.24		0.00 [-0.47, 0.47]
Cahn 2004	0	0.12		0.00 [-0.24, 0.24]
Heckman 2004	0	0.12		0.00 [-0.24, 0.24]
Mathews 2002	0	0.16		0.00 [-0.31, 0.31]
Li 2010	0.01	0.1		0.01 [-0.19, 0.21]
Gibbie 2007	0.01	0.24		0.01 [-0.46, 0.48]
Parutti 2006	0.01	0.16		0.01 [-0.30, 0.32]
Kleeberger 2001	0.01	0.08		0.01 [-0.15, 0.17]
Unge 2010	0.01	0.1		0.01 [-0.19, 0.21]
Venkatesh 2010	0.01	0.14		0.01 [-0.26, 0.28]
Sodergard 2006	0.02	0.07		0.02 [-0.12, 0.16]
Leserman 2008	0.02	0.2		0.02 [-0.37, 0.41]
Cooper 2010	0.02	0.21		0.02 [-0.39, 0.43]
Franke 2010	0.02	0.46		0.02 [-0.88, 0.92]
Jacquet 2010	0.03	0.08		0.03 [-0.13, 0.19]
Dilorio 2007	0.03	0.32		0.03 [-0.60, 0.66]
Thrasher 2008	0.05	0.04		0.05 [-0.03, 0.13]

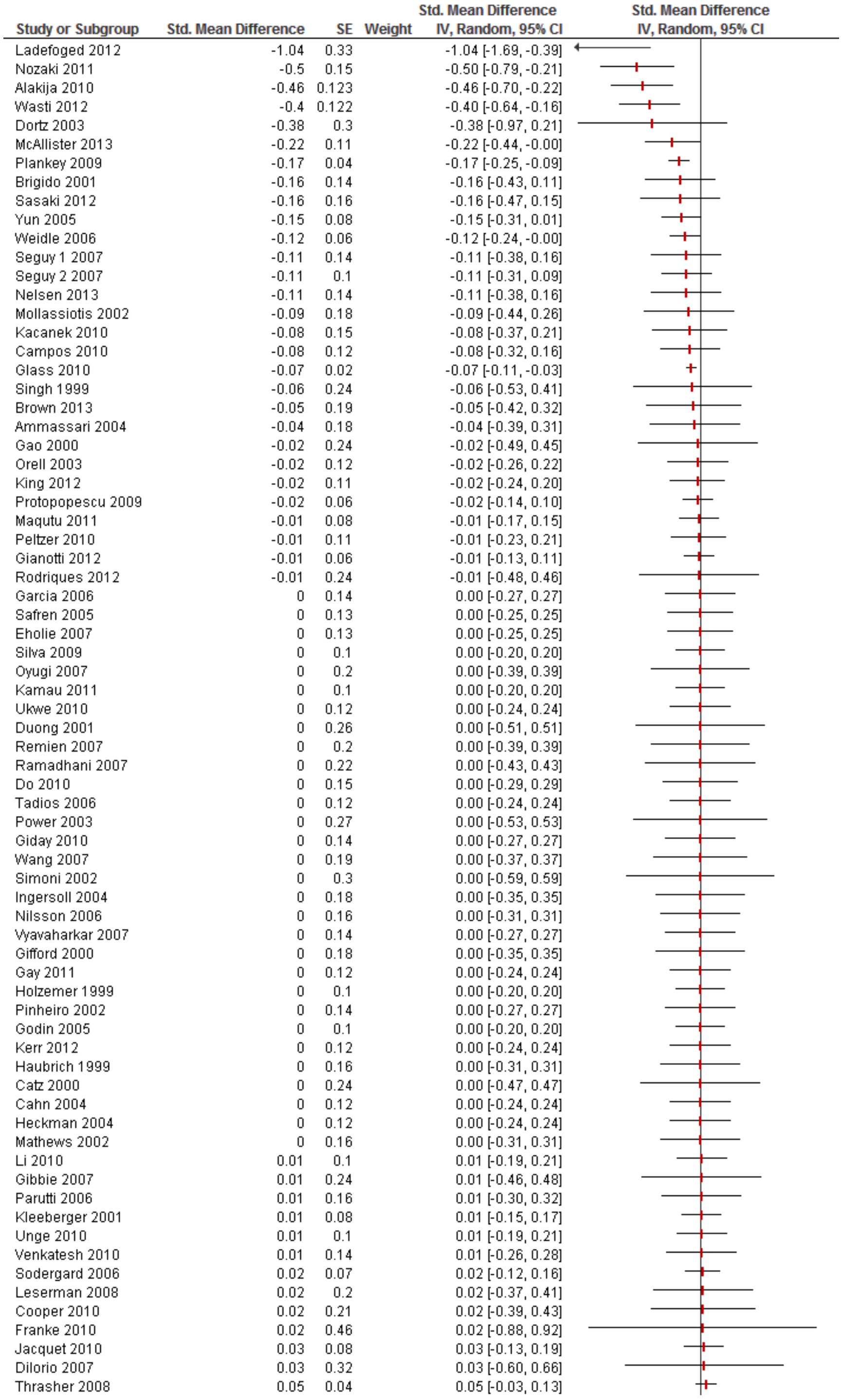

Blackstock 2012	0.06	0.16	0.06 [-0.25, 0.37]
Boyer 2011	0.06	0.04	0.06 [-0.02, 0.14]
Fatima 1 2013	0.06	0.16	0.06 [-0.25, 0.37]
Sumari 2011	0.07	0.14	0.07 [-0.20, 0.34]
Etard 2007	0.07	0.19	0.07 [-0.30, 0.44]
OConnor 2013	0.07	0.03	0.07 [0.01, 0.13]
Uuskula 2012	0.07	0.16	0.07 [-0.24, 0.38]
Murphy 2004	0.08	0.18	0.08 [-0.27, 0.43]
Colbert 2013	0.08	0.19	0.08 [-0.29, 0.45]
Sellier 2006	0.08	0.28	0.08 [-0.47, 0.63]
Gordillo 1999	0.09	0.1	0.09 [-0.11, 0.29]
Cambiano 2010	0.09	0.04	0.09 [0.01, 0.17]
Lazo 2 2007	0.1	0.06	0.10 [-0.02, 0.22]
Oku 2014	0.11	0.1	0.11 [-0.09, 0.31]
Rintamaki 2006	0.11	0.14	0.11 [-0.16, 0.38]
Adewuya 2010	0.12	0.17	0.12 [-0.21, 0.45]
Johnson 2003	0.13	0.04	0.13 [0.05, 0.21]
Anuradha 2012	0.13	0.12	0.13 [-0.11, 0.37]
Kalichman 2010	0.14	0.14	0.14 [-0.13, 0.41]
Mellins 2003	0.14	0.261	0.14 [-0.37, 0.65]
Nakimulli 2013	0.16	0.1	0.16 [-0.04, 0.36]
Sayles 2009	0.17	0.17	0.17 [-0.16, 0.50]
Fatima 2 2013	0.18	0.2	0.18 [-0.21, 0.57]
Simoni 2012	0.18	0.04	0.18 [0.10, 0.26]
Fong 2003	0.18	0.2	0.18 [-0.21, 0.57]
Aragones 2011	0.19	0.08	0.19 [0.03, 0.35]
Murri 2001	0.19	0.181	0.19 [-0.16, 0.54]
Feldman 2013	0.2	0.04	0.20 [0.12, 0.28]
Andrade 2013	0.2	0.22	0.20 [-0.23, 0.63]
Sherr 2008	0.2	0.08	0.20 [0.04, 0.36]
Servellen 2002	0.2	0.141	0.20 [-0.08, 0.48]
Mannheimer 2002	0.2	0.06	0.20 [0.08, 0.32]
Ford 2010	0.2	0.141	0.20 [-0.08, 0.48]
Bianco 1 2011	0.2	0.161	0.20 [-0.12, 0.52]
Woods 2009	0.2	0.221	0.20 [-0.23, 0.63]
Kyser 2011	0.21	0.12	0.21 [-0.03, 0.45]
Sullivan 2007	0.21	0.02	0.21 [0.17, 0.25]
Mugavero 2009	0.21	0.121	0.21 [-0.03, 0.45]
Malow 2013	0.22	0.14	0.22 [-0.05, 0.49]
Juday 2011	0.22	0.101	0.22 [0.02, 0.42]
Finnocchario 2011	0.22	0.161	0.22 [-0.10, 0.54]
Luszczynska 2007	0.22	0.201	0.22 [-0.17, 0.61]
Peretti 1 2006	0.23	0.08	0.23 [0.07, 0.39]
Boarts 2006	0.23	0.27	0.23 [-0.30, 0.76]
Gauchet 2007	0.23	0.181	0.23 [-0.12, 0.58]
Raboud 2011	0.24	0.1	0.24 [0.04, 0.44]
Nakimuli 2009	0.24	0.2	0.24 [-0.15, 0.63]
Moralejo 2006	0.25	0.161	0.25 [-0.07, 0.57]
Trotta 2003	0.26	0.08	0.26 [0.10, 0.42]
Peretti 2 2006	0.27	0.081	0.27 [0.11, 0.43]
Lazo 1 2007	0.27	0.081	0.27 [0.11, 0.43]
Shuter 2008	0.27	0.263	0.27 [-0.25, 0.79]
Kalichman 1999	0.28	0.141	0.28 [0.00, 0.56]
Diabate 2007	0.29	0.09	0.29 [0.11, 0.47]
Falang 2012	0.29	0.101	0.29 [0.09, 0.49]
Wagner 2011	0.3	0.142	0.30 [0.02, 0.58]
Schneider 2004	0.3	0.09	0.30 [0.12, 0.48]
Duggan 2009	0.3	0.182	0.30 [-0.06, 0.66]
Howard 2002	0.31	0.162	0.31 [-0.01, 0.63]
Kumar 2009	0.31	0.061	0.31 [0.19, 0.43]
Spire 2002	0.32	0.101	0.32 [0.12, 0.52]
Peretti 3 2006	0.33	0.101	0.33 [0.13, 0.53]
Cardarelli 2008	0.34	0.203	0.34 [-0.06, 0.74]
Gonzalez 2007	0.34	0.122	0.34 [0.10, 0.58]
Bottonari 2012	0.37	0.143	0.37 [0.09, 0.65]
Bianco 2 2011	0.38	0.224	0.38 [-0.06, 0.82]
Golin 2002	0.38	0.183	0.38 [0.02, 0.74]
Sherr 2010	0.42	0.102	0.42 [0.22, 0.62]
Hansana 2013	0.42	0.11	0.42 [0.20, 0.64]
Leombruni 2009	0.42	0.184	0.42 [0.06, 0.78]
Weaver 2005	0.43	0.123	0.43 [0.19, 0.67]
Gokarn 2012	0.47	0.14	0.47 [0.20, 0.74]
Kunutsor 2010	0.47	0.11	0.47 [0.25, 0.69]
Ettenhofer 2009	0.51	0.1	0.51 [0.31, 0.71]
Paterson 2000	0.52	0.27	0.52 [-0.01, 1.05]
Gonzalez 2004	0.52	0.227	0.52 [0.08, 0.96]
Carballo 2004	0.54	0.13	0.54 [0.29, 0.79]
Poquette 2013	0.56	0.25	0.56 [0.07, 1.05]
Shah 2007	0.56	0.14	0.56 [0.29, 0.83]
Ickovics 2002	0.59	0.229	0.59 [0.14, 1.04]
Carmody 2003	0.6	0.35	0.60 [-0.09, 1.29]
Nieuwkerk 2001	0.63	0.168	0.63 [0.30, 0.96]
Cauldbeck 2009	0.79	0.302	0.79 [0.20, 1.38]
Horne 2007	0.8	0.194	0.80 [0.42, 1.18]
Wagner 2002	0.84	0.282	0.84 [0.29, 1.39]

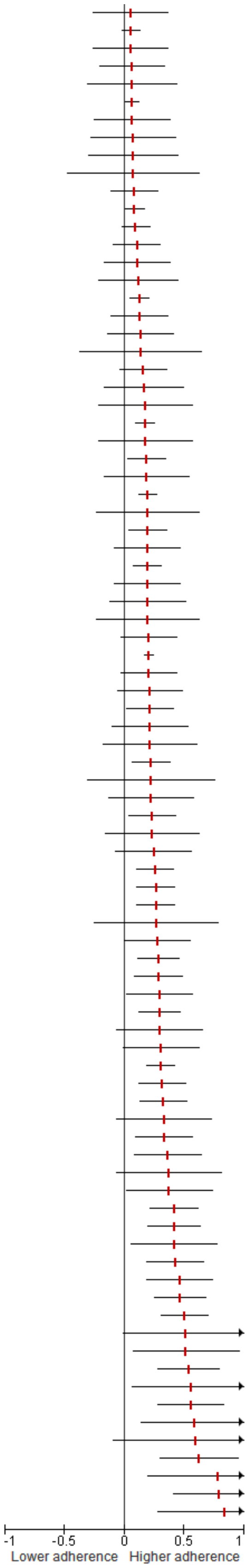

Time since HIV diagnosis

Study or Subgroup	Std. Mean Difference	SE	Weight	Std. Mean Difference IV, Random, 95% CI
Fong 2003	-0.77	0.2		-0.77 [-1.16, -0.38]
Cauldbeck 2009	-0.68	0.296		-0.68 [-1.26, -0.10]
Holstad 2006	-0.47	0.185		-0.47 [-0.83, -0.11]
Huynh 2013	-0.47	0.18		-0.47 [-0.82, -0.12]
Bonolo 2005	-0.45	0.12		-0.45 [-0.69, -0.21]
Horne 2007	-0.44	0.184		-0.44 [-0.80, -0.08]
Weaver 2005	-0.41	0.122		-0.41 [-0.65, -0.17]
Gauchet 2007	-0.38	0.183		-0.38 [-0.74, -0.02]
Blackstock 2012	-0.36	0.163		-0.36 [-0.68, -0.04]
Glass 2010	-0.35	0.02		-0.35 [-0.39, -0.31]
Kyser 2011	-0.32	0.12		-0.32 [-0.56, -0.08]
Woods 2009	-0.32	0.223		-0.32 [-0.76, 0.12]
Silva 2009	-0.31	0.1		-0.31 [-0.51, -0.11]
Carballo 2004	-0.27	0.13		-0.27 [-0.52, -0.02]
Leombruni 2009	-0.25	0.181		-0.25 [-0.60, 0.10]
Kalichman 2008	-0.23	0.19		-0.23 [-0.60, 0.14]
Adewuya 2010	-0.22	0.17		-0.22 [-0.55, 0.11]
Tedaldi 2012	-0.22	0.09		-0.22 [-0.40, -0.04]
Seguy 2 2007	-0.2	0.1		-0.20 [-0.40, -0.00]
Bianco 2 2011	-0.2	0.221		-0.20 [-0.63, 0.23]
Nelsen 2013	-0.2	0.14		-0.20 [-0.47, 0.07]
Golin 2002	-0.18	0.181		-0.18 [-0.53, 0.17]
Juday 2011	-0.17	0.1		-0.17 [-0.37, 0.03]
Peretti 1 2006	-0.13	0.08		-0.13 [-0.29, 0.03]
Sayles 2009	-0.11	0.17		-0.11 [-0.44, 0.22]
Sasaki 2012	-0.1	0.16		-0.10 [-0.41, 0.21]
Peretti 2 2006	-0.1	0.08		-0.10 [-0.26, 0.06]
Wagner 2011	-0.08	0.14		-0.08 [-0.35, 0.19]
Raboud 2011	-0.06	0.1		-0.06 [-0.26, 0.14]
Aragones 2011	-0.04	0.08		-0.04 [-0.20, 0.12]
Kalichman 1999	-0.03	0.14		-0.03 [-0.30, 0.24]
Spire 2002	-0.03	0.1		-0.03 [-0.23, 0.17]
Godin 2005	0	0.1		0.00 [-0.20, 0.20]
Gonzalez 2007	0	0.12		0.00 [-0.24, 0.24]
Thrasher 2008	0	0.04		0.00 [-0.08, 0.08]
Nilsson 2006	0	0.16		0.00 [-0.31, 0.31]
Barclay 2007	0	0.14		0.00 [-0.27, 0.27]
Simoni 2002	0	0.3		0.00 [-0.59, 0.59]
Kerr 2012	0	0.12		0.00 [-0.24, 0.24]
Mollassiotis 2002	0	0.18		0.00 [-0.35, 0.35]
Catz 2000	0	0.24		0.00 [-0.47, 0.47]
Eholie 2007	0	0.13		0.00 [-0.25, 0.25]
Gonzalez 2004	0	0.22		0.00 [-0.43, 0.43]
Boyer 2011	0	0.04		0.00 [-0.08, 0.08]
Rodrigues 2012	0.01	0.24		0.01 [-0.46, 0.48]
Sumari 2011	0.02	0.14		0.02 [-0.25, 0.29]
Li 2010	0.03	0.1		0.03 [-0.17, 0.23]
Johnson 2003	0.06	0.04		0.06 [-0.02, 0.14]
Mellins 2003	0.08	0.26		0.08 [-0.43, 0.59]
Uuskula 2012	0.11	0.16		0.11 [-0.20, 0.42]
Peretti 3 2006	0.12	0.1		0.12 [-0.08, 0.32]
Protopopescu 2009	0.13	0.06		0.13 [0.01, 0.25]
Seguy 1 2007	0.13	0.14		0.13 [-0.14, 0.40]
Gay 2011	0.13	0.12		0.13 [-0.11, 0.37]
Hansana 2013	0.14	0.11		0.14 [-0.08, 0.36]
Bianco 1 2011	0.16	0.161		0.16 [-0.16, 0.48]
Peltzer 2010	0.57	0.12		0.57 [0.33, 0.81]

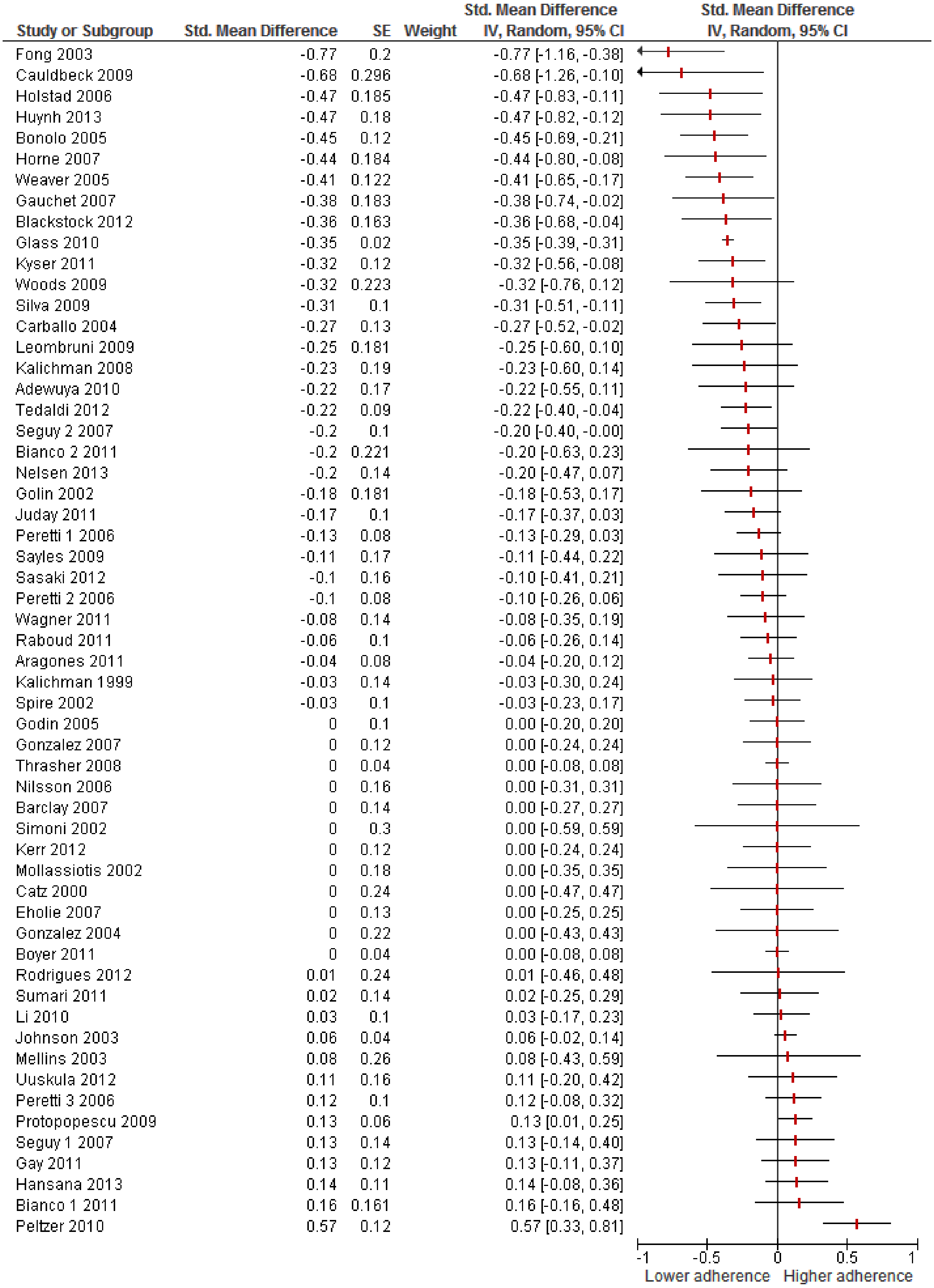

CD4 cell count

Study or Subgroup	Std. Mean Difference	SE	Weight	Std. Mean Difference IV, Random, 95% CI
Fatima 1 2013	-0.76	0.16		-0.76 [-1.07, -0.45]
Howard 2002	-0.62	0.168		-0.62 [-0.95, -0.29]
Gifford 2000	-0.6	0.188		-0.60 [-0.97, -0.23]
Weidle 2006	-0.59	0.07		-0.59 [-0.73, -0.45]
Shuter 2008	-0.58	0.271		-0.58 [-1.11, -0.05]
Lazo 1 2007	-0.38	0.081		-0.38 [-0.54, -0.22]
Dale 2014	-0.36	0.17		-0.36 [-0.69, -0.03]
Nelsen 2013	-0.34	0.14		-0.34 [-0.61, -0.07]
Sayles 2009	-0.34	0.17		-0.34 [-0.67, -0.01]
Campos 2010	-0.33	0.142		-0.33 [-0.61, -0.05]
Bonolo 2005	-0.33	0.12		-0.33 [-0.57, -0.09]
Trotta 2003	-0.29	0.08		-0.29 [-0.45, -0.13]
Diabate 2007	-0.28	0.09		-0.28 [-0.46, -0.10]
Mellins 2003	-0.28	0.263		-0.28 [-0.80, 0.24]
Venkatesh 2010	-0.25	0.141		-0.25 [-0.53, 0.03]
Lazo 2 2007	-0.23	0.06		-0.23 [-0.35, -0.11]
Nieuwkerk 2001	-0.21	0.161		-0.21 [-0.53, 0.11]
Golin 2002	-0.18	0.181		-0.18 [-0.53, 0.17]
Leombruni 2009	-0.15	0.181		-0.15 [-0.50, 0.20]
Busher 2012	-0.15	0.2		-0.15 [-0.54, 0.24]
Huynh 2013	-0.15	0.17		-0.15 [-0.48, 0.18]
Horne 2007	-0.1	0.18		-0.10 [-0.45, 0.25]
Pefura 2014	-0.09	0.07		-0.09 [-0.23, 0.05]
Gokarn 2012	-0.09	0.14		-0.09 [-0.36, 0.18]
Fatima 2 2013	-0.08	0.19		-0.08 [-0.45, 0.29]
Protopopescu 2009	-0.06	0.06		-0.06 [-0.18, 0.06]
Kyser 2011	-0.05	0.12		-0.05 [-0.29, 0.19]
Jacquet 2010	-0.03	0.08		-0.03 [-0.19, 0.13]
Franke 2010	-0.02	0.46		-0.02 [-0.92, 0.88]
Rodrigues 2012	-0.01	0.24		-0.01 [-0.48, 0.46]
Orrell 2003	0	0.12		0.00 [-0.24, 0.24]
Maqutu 2011	0	0.08		0.00 [-0.16, 0.16]
Frain 2009	0	0.24		0.00 [-0.47, 0.47]
Godin 2005	0	0.1		0.00 [-0.20, 0.20]
Ramadhani 2007	0	0.22		0.00 [-0.43, 0.43]
Nilsson 2006	0	0.16		0.00 [-0.31, 0.31]
Oyugi 2007	0	0.2		0.00 [-0.39, 0.39]
Wagner 2002	0	0.26		0.00 [-0.51, 0.51]
Gonzalez 2004	0	0.22		0.00 [-0.43, 0.43]
Mannheimer 2002	0	0.06		0.00 [-0.12, 0.12]
Brigido 2001	0.01	0.14		0.01 [-0.26, 0.28]
Cooper 2010	0.01	0.21		0.01 [-0.40, 0.42]
Spire 2002	0.03	0.1		0.03 [-0.17, 0.23]
Elul 2013	0.03	0.06		0.03 [-0.09, 0.15]
Andrade 2013	0.04	0.22		0.04 [-0.39, 0.47]
Seguy 1 2007	0.05	0.14		0.05 [-0.22, 0.32]
Ford 2010	0.05	0.14		0.05 [-0.22, 0.32]
Johnson 2003	0.06	0.04		0.06 [-0.02, 0.14]
Rougemont 2009	0.07	0.14		0.07 [-0.20, 0.34]
Thrasher 2008	0.08	0.04		0.08 [0.00, 0.16]
Glass 2010	0.09	0.2		0.09 [-0.30, 0.48]
Boyer 2011	0.1	0.04		0.10 [0.02, 0.18]
Gianotti 2012	0.12	0.06		0.12 [0.00, 0.24]
Woods 2009	0.13	0.221		0.13 [-0.30, 0.56]
Peltzer 2010	0.14	0.12		0.14 [-0.10, 0.38]
Gay 2011	0.18	0.12		0.18 [-0.06, 0.42]
Carmody 2003	0.23	0.34		0.23 [-0.44, 0.90]
Anuradha 2012	0.25	0.121		0.25 [0.01, 0.49]
Kalichman 1999	0.3	0.142		0.30 [0.02, 0.58]
Kalichman 2010	0.35	0.16		0.35 [0.04, 0.66]
Finocchario 2011	0.36	0.163		0.36 [0.04, 0.68]
Kleeberger 2001	0.4	0.082		0.40 [0.24, 0.56]
Seguy 2 2007	0.49	0.1		0.49 [0.29, 0.69]
Uuskula 2012	0.52	0.165		0.52 [0.20, 0.84]
Gordillo 1999	0.59	0.104		0.59 [0.39, 0.79]
Ickovics 2002	0.73	0.234		0.73 [0.27, 1.19]
Berhe 2013	1.38	0.12		1.38 [1.14, 1.62]

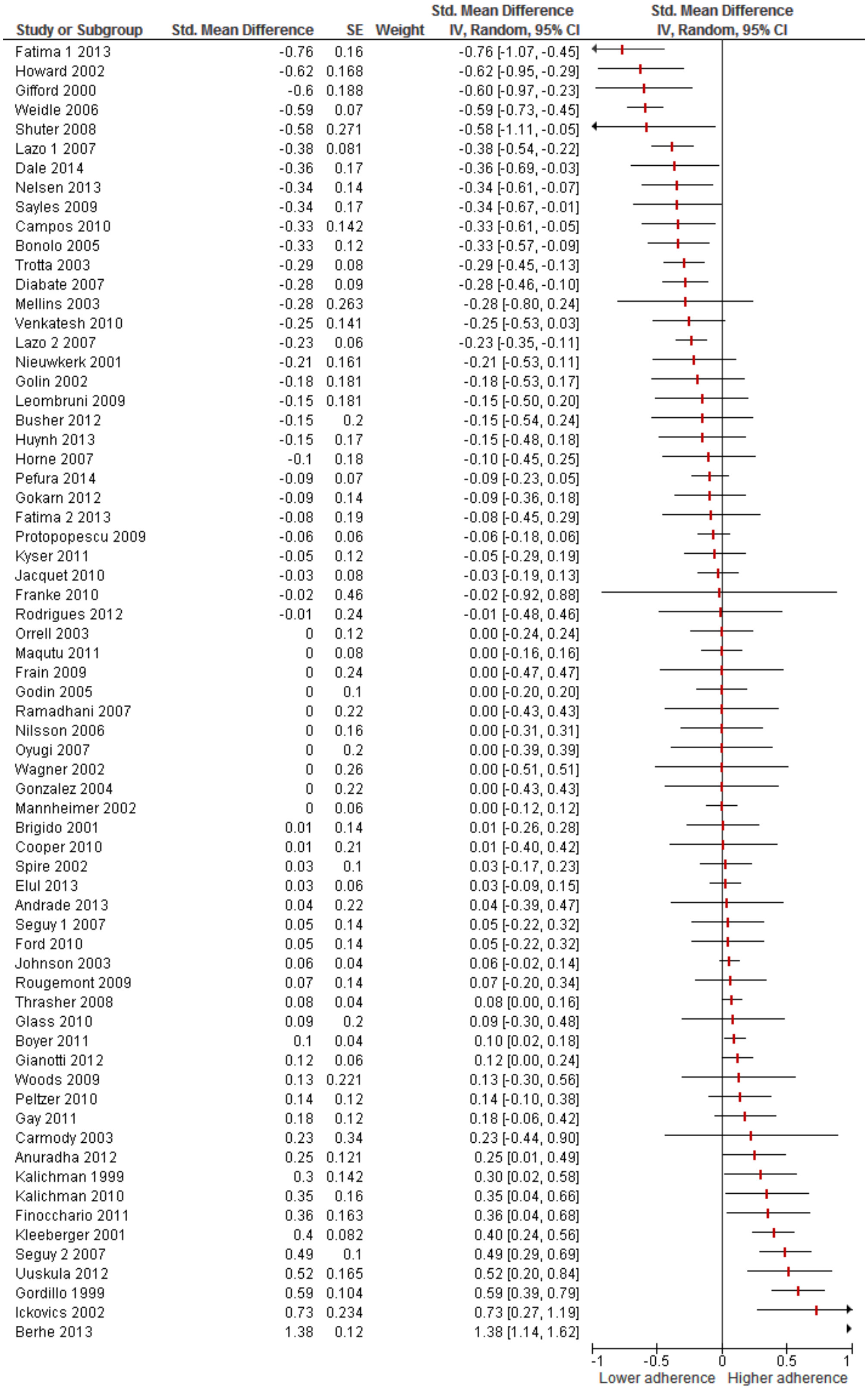

Male gender

Study or Subgroup	Std. Mean Difference	SE	Weight	Std. Mean Difference IV, Random, 95% CI
Ramadhani 2007	-1.99	0.25		-1.99 [-2.48, -1.50]
Woods 2009	-0.73	0.234		-0.73 [-1.19, -0.27]
Busher 2012	-0.62	0.21		-0.62 [-1.03, -0.21]
Tedaldi 2012	-0.58	0.09		-0.58 [-0.76, -0.40]
Sasaki 2012	-0.47	0.16		-0.47 [-0.78, -0.16]
Gauchet 2007	-0.45	0.184		-0.45 [-0.81, -0.09]
Fong 2003	-0.43	0.2		-0.43 [-0.82, -0.04]
Finnocchario 2011	-0.38	0.163		-0.38 [-0.70, -0.06]
Weidle 2006	-0.35	0.06		-0.35 [-0.47, -0.23]
Nelsen 2013	-0.35	0.14		-0.35 [-0.62, -0.08]
Ladefoged 2012	-0.34	0.32		-0.34 [-0.97, 0.29]
Rougemont 2009	-0.33	0.142		-0.33 [-0.61, -0.05]
King 2012	-0.3	0.11		-0.30 [-0.52, -0.08]
Bell 2007	-0.28	0.263		-0.28 [-0.80, 0.24]
Pinheiro 2002	-0.28	0.141		-0.28 [-0.56, -0.00]
Poquette 2013	-0.26	0.25		-0.26 [-0.75, 0.23]
Kamau 2011	-0.25	0.101		-0.25 [-0.45, -0.05]
Adewuya 2010	-0.24	0.17		-0.24 [-0.57, 0.09]
Negash 2013	-0.22	0.12		-0.22 [-0.46, 0.02]
Cooper 2010	-0.21	0.22		-0.21 [-0.64, 0.22]
Protopopescu 2009	-0.19	0.06		-0.19 [-0.31, -0.07]
Gokarn 2012	-0.18	0.14		-0.18 [-0.45, 0.09]
Shuter 2008	-0.18	0.261		-0.18 [-0.69, 0.33]
Blackstock 2012	-0.18	0.161		-0.18 [-0.50, 0.14]
Brigido 2001	-0.17	0.141		-0.17 [-0.45, 0.11]
Dortz 2003	-0.17	0.3		-0.17 [-0.76, 0.42]
Uuskula 2012	-0.16	0.161		-0.16 [-0.48, 0.16]
Venkatesh 2010	-0.14	0.14		-0.14 [-0.41, 0.13]
Boyer 2011	-0.13	0.04		-0.13 [-0.21, -0.05]
Pefura 2013	-0.11	0.07		-0.11 [-0.25, 0.03]
Juday 2011	-0.1	0.1		-0.10 [-0.30, 0.10]
Graham 2007	-0.09	0.21		-0.09 [-0.50, 0.32]
Seguy 1 2007	-0.09	0.14		-0.09 [-0.36, 0.18]
Ford 2010	-0.08	0.14		-0.08 [-0.35, 0.19]
Garcia 2009	-0.08	0.14		-0.08 [-0.35, 0.19]
Mugavero 2009	-0.07	0.12		-0.07 [-0.31, 0.17]
Etienne 2010	-0.07	0.07		-0.07 [-0.21, 0.07]
Kalichman 2010	-0.07	0.14		-0.07 [-0.34, 0.20]
Li 2010	-0.04	0.1		-0.04 [-0.24, 0.16]
Pratt 2001	-0.03	0.12		-0.03 [-0.27, 0.21]
Jacquet 2010	-0.02	0.08		-0.02 [-0.18, 0.14]
Parruti 2006	-0.02	0.16		-0.02 [-0.33, 0.29]
Orell 2003	0	0.12		0.00 [-0.24, 0.24]
Giday 2010	0	0.14		0.00 [-0.27, 0.27]
Silva 2009	0	0.1		0.00 [-0.20, 0.20]
Remien 2007	0	0.2		0.00 [-0.39, 0.39]
Barclay 2007	0	0.14		0.00 [-0.27, 0.27]
Gonzalez 2004	0	0.22		0.00 [-0.43, 0.43]
Murphy 2004	0	0.19		0.00 [-0.37, 0.37]
Mathews 2002	0	0.16		0.00 [-0.31, 0.31]
Seguy 2 2007	0	0.1		0.00 [-0.20, 0.20]
Do 2010	0	0.15		0.00 [-0.29, 0.29]
Eholie 2007	0	0.13		0.00 [-0.25, 0.25]
Wang 2007	0	0.19		0.00 [-0.37, 0.37]
Haubrich 1999	0	0.16		0.00 [-0.31, 0.31]
Power 2003	0	0.27		0.00 [-0.53, 0.53]
Murri 2001	0	0.18		0.00 [-0.35, 0.35]
Nilsson 2006	0	0.16		0.00 [-0.31, 0.31]
Safren 2005	0	0.13		0.00 [-0.25, 0.25]
Catz 2000	0	0.24		0.00 [-0.47, 0.47]
Heckman 2004	0	0.12		0.00 [-0.24, 0.24]
Holzemer 1999	0	0.1		0.00 [-0.20, 0.20]
Oyugi 2007	0	0.2		0.00 [-0.39, 0.39]
Mannheimer 2002	0	0.06		0.00 [-0.12, 0.12]
Simoni 2002	0	0.3		0.00 [-0.59, 0.59]
Kerr 2012	0	0.12		0.00 [-0.24, 0.24]
Ingersoll 2004	0	0.18		0.00 [-0.35, 0.35]
Mollassiotis 2002	0	0.18		0.00 [-0.35, 0.35]

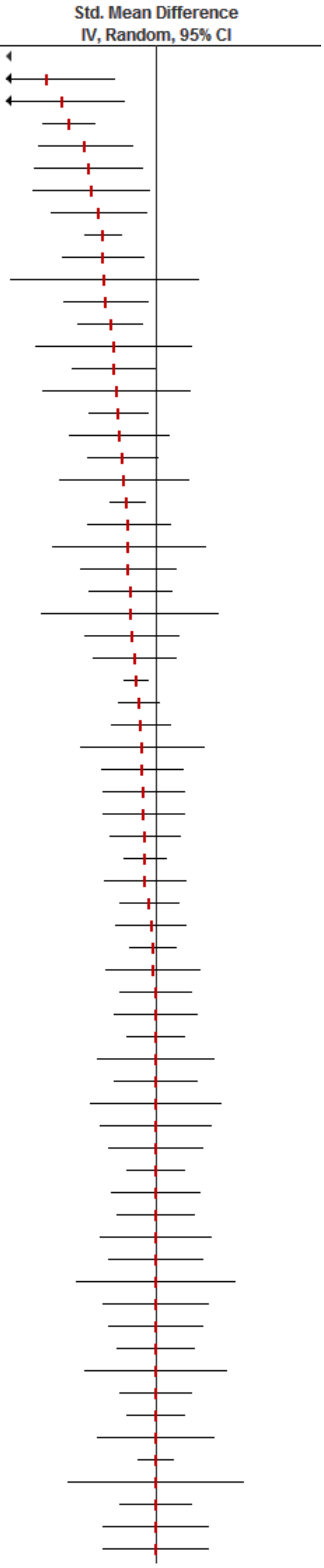

McAllister 2013	0.001	0.11	0.00 [-0.21, 0.22]
Kunutsor 2010	0.01	0.11	0.01 [-0.21, 0.23]
Dilorio 2007	0.02	0.13	0.02 [-0.23, 0.27]
Elul 2013	0.03	0.06	0.03 [-0.09, 0.15]
Spire 2002	0.04	0.1	0.04 [-0.16, 0.24]
Ammassari 2004	0.04	0.18	0.04 [-0.31, 0.39]
Rintamaki 2006	0.05	0.14	0.05 [-0.22, 0.32]
Wanchu 2006	0.06	0.16	0.06 [-0.25, 0.37]
Peltzer 2010	0.07	0.13	0.07 [-0.18, 0.32]
Ferguson 2002	0.07	0.16	0.07 [-0.24, 0.38]
OConnor 2013	0.08	0.03	0.08 [0.02, 0.14]
Etard 2007	0.08	0.17	0.08 [-0.25, 0.41]
Bottonari 2012	0.08	0.14	0.08 [-0.19, 0.35]
Holstad 2006	0.08	0.18	0.08 [-0.27, 0.43]
Bianco 2011	0.08	0.12	0.08 [-0.16, 0.32]
Golin 2002	0.09	0.18	0.09 [-0.26, 0.44]
Anuradha 2012	0.09	0.12	0.09 [-0.15, 0.33]
Johnson 2003	0.09	0.04	0.09 [0.01, 0.17]
Aragones 2011	0.09	0.08	0.09 [-0.07, 0.25]
Sherr 2010	0.09	0.1	0.09 [-0.11, 0.29]
Cauldbeck 2009	0.1	0.28	0.10 [-0.45, 0.65]
Falang 2012	0.1	0.1	0.10 [-0.10, 0.30]
Colbert 2013	0.11	0.19	0.11 [-0.26, 0.48]
Simoni 2012	0.12	0.04	0.12 [0.04, 0.20]
Feldman 2013	0.12	0.04	0.12 [0.04, 0.20]
Nakimuli 2009	0.13	0.2	0.13 [-0.26, 0.52]
Servellen 2002	0.13	0.14	0.13 [-0.14, 0.40]
Nakimulli 2013	0.14	0.1	0.14 [-0.06, 0.34]
Glass 2010	0.19	0.02	0.19 [0.15, 0.23]
Moralejo 2006	0.2	0.161	0.20 [-0.12, 0.52]
Raboud 2011	0.2	0.1	0.20 [0.00, 0.40]
Ubbiali 2008	0.23	0.101	0.23 [0.03, 0.43]
Ukwe 2010	0.24	0.121	0.24 [0.00, 0.48]
Thrasher 2008	0.25	0.04	0.25 [0.17, 0.33]
Nieuwkerk 2001	0.26	0.161	0.26 [-0.06, 0.58]
Kumar 2009	0.27	0.061	0.27 [0.15, 0.39]
Yun 2005	0.27	0.081	0.27 [0.11, 0.43]
Oku 2014	0.27	0.1	0.27 [0.07, 0.47]
Luszczynska 2007	0.28	0.202	0.28 [-0.12, 0.68]
Kyser 2011	0.29	0.12	0.29 [0.05, 0.53]
Cahn 2004	0.3	0.121	0.30 [0.06, 0.54]
Leombruni 2009	0.3	0.182	0.30 [-0.06, 0.66]
Nozaki 2011	0.31	0.15	0.31 [0.02, 0.60]
Tiyou 2010	0.31	0.122	0.31 [0.07, 0.55]
Rodrigues 2012	0.32	0.24	0.32 [-0.15, 0.79]
Kalichman 1999	0.32	0.142	0.32 [0.04, 0.60]
Birbeck 2011	0.32	0.101	0.32 [0.12, 0.52]
Cardarelli 2008	0.34	0.203	0.34 [-0.06, 0.74]
Campos 2010	0.35	0.122	0.35 [0.11, 0.59]
Bonolo 2005	0.35	0.12	0.35 [0.11, 0.59]
Gifford 2000	0.36	0.183	0.36 [0.00, 0.72]
Malow 2013	0.37	0.14	0.37 [0.10, 0.64]
Huynh 2013	0.39	0.17	0.39 [0.06, 0.72]
Wasti 2012	0.4	0.122	0.40 [0.16, 0.64]
Sayles 2009	0.4	0.17	0.40 [0.07, 0.73]
Andrade 2013	0.43	0.23	0.43 [-0.02, 0.88]
Leserman 2008	0.43	0.2	0.43 [0.04, 0.82]
Duong 2001	0.43	0.27	0.43 [-0.10, 0.96]
Duggan 2009	0.44	0.184	0.44 [0.08, 0.80]
Sumari 2011	0.46	0.144	0.46 [0.18, 0.74]
Wagner 2002	0.51	0.268	0.51 [-0.02, 1.04]
Hanif 2013	0.52	0.08	0.52 [0.36, 0.68]
Unge 2010	0.53	0.103	0.53 [0.33, 0.73]
Sellier 2006	0.56	0.28	0.56 [0.01, 1.11]
Brown 2013	0.58	0.19	0.58 [0.21, 0.95]
Paterson 2000	0.58	0.28	0.58 [0.03, 1.13]
Kacanek 2010	0.63	0.15	0.63 [0.34, 0.92]
Franke 2010	0.7	0.46	0.70 [-0.20, 1.60]
Alakija 2010	0.74	0.128	0.74 [0.49, 0.99]
Gay 2011	0.74	0.128	0.74 [0.49, 0.99]
Godin 2005	0.84	0.109	0.84 [0.63, 1.05]
Maqutu 2011	0.95	0.08	0.95 [0.79, 1.11]
Ickovics 2002	1.05	0.249	1.05 [0.56, 1.54]
Carmody 2003	1.09	0.36	1.09 [0.38, 1.80]

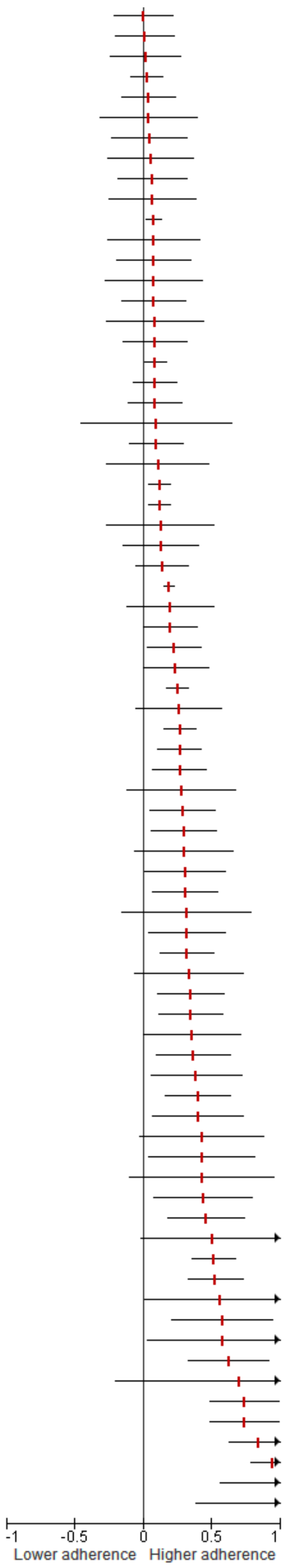

Duration of ART

Study or Subgroup	Std. Mean Difference	SE	Weight	Std. Mean Difference IV, Random, 95% CI
Brown 2013	-0.47	0.19		-0.47 [-0.84, -0.10]
Holstad 2006	-0.43	0.184		-0.43 [-0.79, -0.07]
Garcia 2006	-0.41	0.143		-0.41 [-0.69, -0.13]
Blackstock 2012	-0.33	0.162		-0.33 [-0.65, -0.01]
Venkatesh 2010	-0.31	0.142		-0.31 [-0.59, -0.03]
Pefura 2013	-0.27	0.07		-0.27 [-0.41, -0.13]
Maggiolo 2002	-0.27	0.08		-0.27 [-0.43, -0.11]
Sellier 2006	-0.25	0.28		-0.25 [-0.80, 0.30]
Poquette 2013	-0.25	0.25		-0.25 [-0.74, 0.24]
Golin 2002	-0.22	0.181		-0.22 [-0.57, 0.13]
Pinheiro 2002	-0.2	0.141		-0.20 [-0.48, 0.08]
Bell 2007	-0.18	0.261		-0.18 [-0.69, 0.33]
Etard 2007	-0.16	0.19		-0.16 [-0.53, 0.21]
Sullivan 2007	-0.14	0.2		-0.14 [-0.53, 0.25]
Anuradha 2012	-0.13	0.12		-0.13 [-0.37, 0.11]
Kacanek 2010	-0.08	0.15		-0.08 [-0.37, 0.21]
Tran 2013	-0.07	0.07		-0.07 [-0.21, 0.07]
Sodergard 2006	-0.05	0.07		-0.05 [-0.19, 0.09]
Woods 2009	-0.05	0.22		-0.05 [-0.48, 0.38]
Raboud 2011	-0.04	0.1		-0.04 [-0.24, 0.16]
Cauldbeck 2009	-0.04	0.28		-0.04 [-0.59, 0.51]
Glass 2010	-0.01	0.02		-0.01 [-0.05, 0.03]
Safren 2005	0	0.13		0.00 [-0.25, 0.25]
Catz 2000	0	0.24		0.00 [-0.47, 0.47]
Silva 2009	0	0.1		0.00 [-0.20, 0.20]
Ramadhani 2007	0	0.22		0.00 [-0.43, 0.43]
Do 2010	0	0.15		0.00 [-0.29, 0.29]
Gonzalez 2004	0	0.22		0.00 [-0.43, 0.43]
Kerr 2012	0	0.12		0.00 [-0.24, 0.24]
Campbell 2010	0	0.31		0.00 [-0.61, 0.61]
Barclay 2007	0	0.14		0.00 [-0.27, 0.27]
Wang 2007	0	0.19		0.00 [-0.37, 0.37]
Boyer 2011	0.01	0.04		0.01 [-0.07, 0.09]
Aragones 2011	0.01	0.08		0.01 [-0.15, 0.17]
Rodrigues 2012	0.02	0.24		0.02 [-0.45, 0.49]
Cambiano 2010	0.02	0.15		0.02 [-0.27, 0.31]
Sumari 2011	0.02	0.14		0.02 [-0.25, 0.29]
Jacquet 2010	0.04	0.08		0.04 [-0.12, 0.20]
Fong 2003	0.08	0.2		0.08 [-0.31, 0.47]
Nozaki 2011	0.08	0.15		0.08 [-0.21, 0.37]
Adewuya 2010	0.2	0.17		0.20 [-0.13, 0.53]
Nakimuli 2009	0.27	0.2		0.27 [-0.12, 0.66]
Uuskula 2012	0.29	0.162		0.29 [-0.03, 0.61]
Kunutsor 2010	0.3	0.11		0.30 [0.08, 0.52]
Hanif 2013	0.32	0.08		0.32 [0.16, 0.48]
Andrade 2013	0.43	0.23		0.43 [-0.02, 0.88]
Wasti 2012	0.44	0.123		0.44 [0.20, 0.68]
Howard 2002	0.44	0.164		0.44 [0.12, 0.76]
Alakija 2010	0.46	0.123		0.46 [0.22, 0.70]
Unge 2010	0.46	0.103		0.46 [0.26, 0.66]
Ladefoged 2012	0.52	0.32		0.52 [-0.11, 1.15]

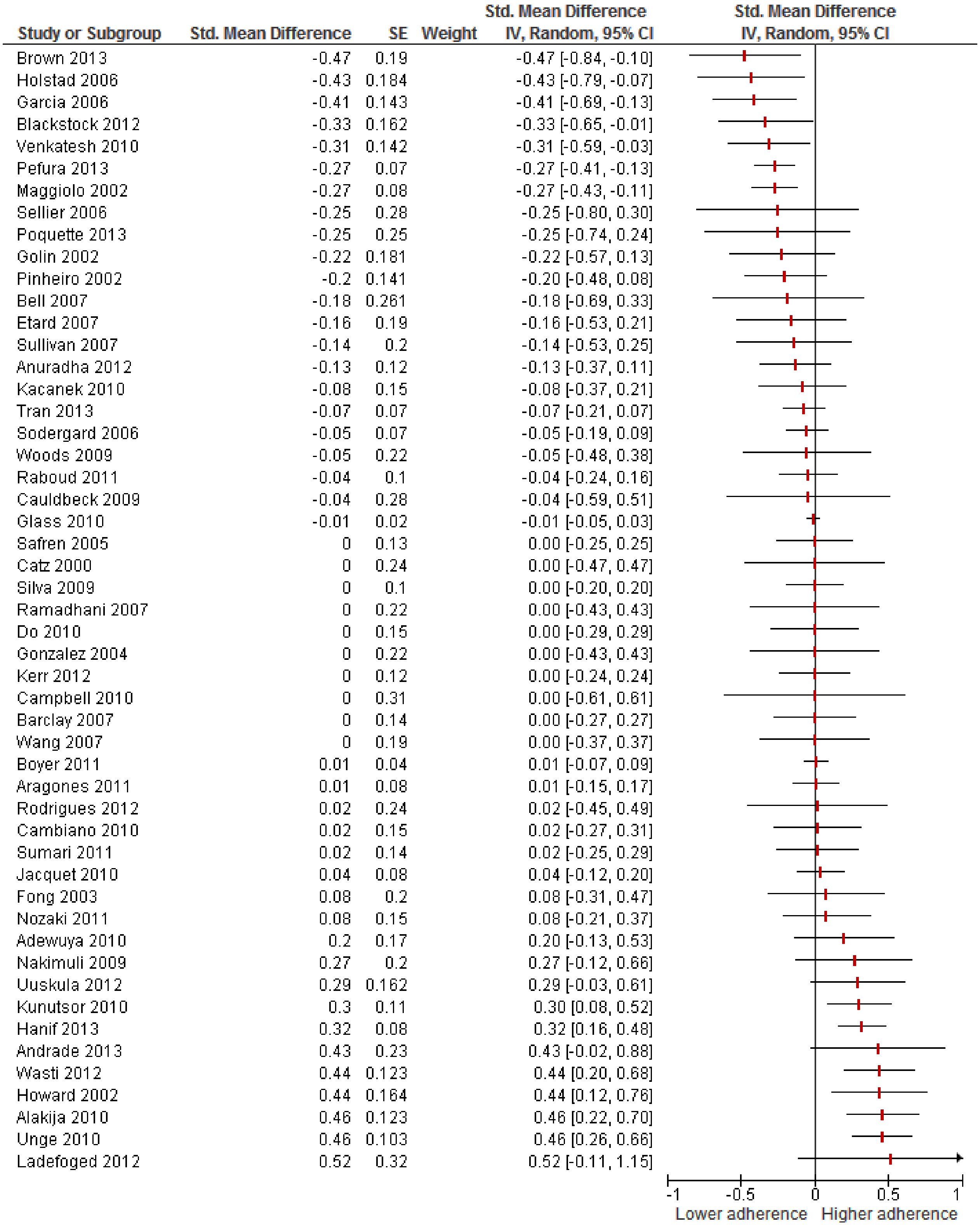

Supplement: Supplementary file 2 — Additional file 2: Forest plots of individual studies examining predictors/correlates.(PDF 432 KB) [file 12916_2014_142_MOESM2_ESM.pdf]
