# Supplementary material for: Predictors and correlates of adherence to combination antiretroviral therapy (ART) for chronic HIV infection: a meta-analysis
Source: BMC Med. 2014 Aug 21;12:142. doi: 10.1186/s12916-014-0142-1 (PMC4148019; doi:10.1186/s12916-014-0142-1)
Supplement: Supplementary file 3 — Additional file 3: Scoring of studies according to Strengthening the Reporting of Observational Studies in Epidemiology (STROBE) criteria.(PDF 270 KB) [file 12916_2014_142_MOESM3_ESM.pdf]

Additional file 3: Scoring of studies according to STROBE criteria

	1	2	3	4	5	6	7	8	9	10	11	12	13	14	15	16	17	18	19	20	21	22
Adewuya 2010	√	√	√	√	√	√	√	√	√	-	√	√	√	√	√	√	√	√	√	√	-	-
Alakija 2010	√	√	√	√	√	√	√	√	-	√	√	√	√	√	√	√	√	-	-	-	-	√
Amberbir 2008	√	√	√	√	√	√	√	√	√	-	√	√	√	√	√	√	√	√	√	√	√	√
Ammasari 2004	-	√	√	√	√	√	√	√	-	-	√	√	-	√	√	√	√	√	√	-	-	√
Andrade 2013	√	√	√	√	√	√	√	√	-	-	√	√	√	√	√	√	√	√	√	√	√	√
Anuradha 2011	√	√	√	√	√	√	√	√	-	√	√	√	√	√	√	√	√	√	-	√	-	√
Aragones 2011	√	√	√	√	√	√	√	√	-	√	√	√	√	√	√	√	√	√	√	√	√	√
Arrivillage 2009	√	√	√	√	√	√	√	√	-	-	√	√	√	√	√	√	√	√	√	-	-	-
Babson 2012	√	√	√	√	√	√	√	√	-	√	√	√	√	√	√	√	√	√	√	√	-	√
Barclay 2007	√	√	√	√	-	-	√	√	-	-	√	-	√	√	√	√	√	√	-	-	-	-
Beach 2005	√	√	√	√	√	√	√	√	-	-	√	√	√	√	√	√	√	√	√	√	√	√
Bell 2007	√	√	√	-	√	√	√	√	-	-	√	-	√	√	√	√	√	√	√	√	-	-
Berhe 2013	√	√	√	√	√	√	√	√	-	√	√	√	√	√	√	√	√	√	√	-	-	√
Bianco 2010	√	√	√	√	√	√	√	√	-	-	√	√	√	√	√	√	√	√	√	√	-	√
Birbeck 2011	√	√	√	√	√	√	√	√	√	-	√	√	√	√	√	√	√	√	√	√	-	√
Blackstock 2012	√	√	√	√	√	√	√	√	-	√	√	√	√	√	√	√	√	√	√	√	-	√
Boarts 2006	√	√	√	√	√	√	√	√	√	√	√	√	√	√	√	√	√	√	√	√	√	√
De Boer 2008	-	√	-	√	√	√	√	√	-	√	√	√	-	√	√	√	-	√	-	√	-	-
Bonolo 2005	√	√	√	√	√	√	√	√	-	√	√	√	√	√	√	√	√	√	-	-	-	√
Bottonari 2012	√	√	√	√	√	√	√	√	√	-	√	√	√	√	√	√	√	√	√	√	-	√
Boyer 2011	√	√	√	√	√	√	√	√	√	√	√	√	√	√	√	√	√	√	√	√	-	√
Brigido 2001	√	√	√	√	√	√	√	√	√	-	√	√	-	√	√	√	√	√	√	√	-	-
Brown 2013	√	√	√	√	√	√	√	√	-	-	√	√	√	√	√	√	√	√	√	√	√	-
Buscher 2012	√	√	√	√	√	√	√	√	-	-	√	√	√	√	√	√	√	√	√	√	√	√
Cahn 2004	√	√	√	√	√	√	√	√	√	-	√	√	-	√	√	√	√	√	√	√	-	√
Campbell 2010	√	√	√	√	√	√	√	-	-	√	√	√	√	√	√	√	√	√	√	√	-	√
Cambiano 2010	√	√	√	√	√	√	√	√	-	-	√	√	√	√	√	√	√	√	√	√	-	√
Campos 2010	√	√	√	√	√	√	√	√	-	-	√	√	√	√	√	√	√	√	√	√	-	√
Carballo 2004	√	√	√	√	√	√	√	√	-	√	√	√	√	√	√	√	√	√	-	√	-	√

	1	2	3	4	5	6	7	8	9	10	11	12	13	14	15	16	17	18	19	20	21	22
Cardarelli 2008	√	√	√	√	-	√	√	√	-	-	-	√	-	√	√	√	-	√	-	√	-	-
Carmody 2003	√	√	√	√	√	√	√	√	-	-	√	√	√	√	√	√	√	√	√	√	-	-
Catz 2000	√	√	√	-	√	√	√	√	-	-	√	√	-	-	√	√	√	√	√	-	-	-
Cauldbeck 2009	√	√	√	√	√	√	-	√	-	-	-	√	√	√	√	√	√	√	√	√	-	√
Cha 2008	√	√	√	√	√	√	√	√	-	-	√	√	√	√	√	√	√	√	-	√	-	√
Chesney 2000	√	√	√	√	√	√	√	√	-	-	√	√	√	√	√	√	√	√	-	√	-	√
Colbert 2013	√	√	√	√	√	√	√	√	-	-	√	√	√	√	√	√	√	√	√	-	-	√
Cooper 2010	√	√	√	√	√	√	√	√	-	√	√	√	√	√	√	√	√	√	√	√	-	√
Cooper 2011	√	√	√	√	√	√	√	√	-	√	√	√	√	√	√	√	√	√	√	√	√	√
Corless 2013	√	√	-	√	√	√	√	√	-	-	√	√	√	√	√	√	√	√	√	-	-	-
Dale 2014	√	√	√	√	√	√	√	√	-	-	√	√	√	√	√	√	√	√	√	√	√	√
Diabate 2007	√	√	√	√	√	√	√	√	-	√	√	√	√	√	√	√	√	√	-	-	-	√
DiIorio 2009	√	√	√	√	√	√	√	√	-	-	√	√	√	√	√	√	√	√	√	√	√	√
Dlamini 2009	√	√	√	√	√	√	√	√	√	√	√	√	√	√	√	√	√	√	√	√	√	√
Do 2010	√	√	√	√	√	√	√	√	-	√	√	√	√	√	√	√	√	√	√	√	-	√
Dorz 2003	√	√	√	√	√	√	√	√	-	-	√	√	√	√	√	√	√	√	√	√	√	√
Duggan 2009	√	√	√	√	√	√	√	-	√	-	√	√	-	√	√	√	√	√	-	√	-	-
Duong 2001	√	√	√	√	√	√	√	√	-	-	√	√	√	√	√	√	√	√	√	√	-	-
Durante 2003	√	√	√	√	√	√	√	√	-	-	√	√	√	√	√	√	√	√	-	√	-	√
Eholie 2007	√	√	√	√	√	√	√	√	-	-	√	√	√	√	√	√	√	√	-	-	-	-
Elul 2013	√	√	-	√	√	√	√	√	-	√	√	√	√	√	√	√	√	√	√	√	√	√
Etard 2007	√	√	√	√	√	√	√	√	-	-	√	√	√	√	√	√	√	√	-	√	-	-
Etienne 2010	√	√	√	√	√	√	√	√	-	-	√	√	√	√	√	√	√	√	-	-	-	√
Ettenhofer 2009	√	√	√	√	√	√	√	√	-	√	√	√	√	√	√	√	√	√	√	√	√	√
Falang 2012	√	√	√	√	√	√	√	√	-	√	√	√	√	√	√	√	√	√	√	√	-	-
Farley 2010	√	√	√	√	√	√	√	√	-	-	√	√	-	√	√	√	√	√	-	√	-	√
Fatima Bonolo 2013	√	√	√	√	√	√	√	√	-	-	√	√	√	√	√	√	√	√	-	√	-	√
Feldman 2013	√	√	√	√	√	√	√	√	-	-	√	√	√	√	√	√	√	√	√	√	√	√
Ferguson 2002	√	√	√	√	√	√	√	√	-	-	√	√	-	√	√	√	√	√	-	√	√	√
Finnocchario 2011	√	√	√	√	√	√	√	√	-	-	√	√	-	-	√	√	√	√	√	√	-	√

	1	2	3	4	5	6	7	8	9	10	11	12	13	14	15	16	17	18	19	20	21	22
Fong 2003	√	√	-	√	√	√	√	√	-	√	√	√	√	√	√	√	√	√	√	√	-	-
Ford 2010	√	√	√	√	√	√	√	√	-	-	√	√	-	√	√	√	√	√	√	√	-	√
Frain 2009	-	√	-	-	-	-	√	√	-	√	√	√	√	-	√	√	√	√	-	-	√	-
Franke 2010	√	√	√	√	√	√	√	√	-	-	√	√	√	√	√	√	√	√	√	√	√	√
Garcia 2006	√	√	√	√	√	√	√	√	-	-	√	√	√	√	√	√	-	√	-	√	-	-
Gao 2000	√	√	√	√	√	√	√	√	-	-	√	√	√	√	√	√	√	√	-	-	-	-
Gauchet 2007	√	√	√	√	√	√	√	√	-	√	√	√	-	-	√	√	√	√	-	√	-	-
Gay 2011	√	√	√	√	√	√	√	√	-	-	√	√	√	√	√	√	√	√	√	√	√	-
Gianotti 2013	√	√	√	√	√	√	√	√	-	-	√	√	√	√	√	√	√	√	√	√	√	√
Gibbie 2007	√	√	√	-	√	-	√	√	-	-	√	√	-	-	√	√	√	-	-	√	-	√
Giday 2010	√	√	√	√	√	√	√	√	-	-	√	-	√	√	√	√	√	√	-	-	-	√
Gifford 2000	√	√	√	√	-	-	√	√	-	-	√	√	-	√	√	√	√	√	-	-	-	√
Glass 2010	√	√	√	√	√	√	√	√	√	√	√	√	√	√	√	√	√	√	√	√	√	-
Godin 2005	√	√	√	√	√	√	√	√	√	√	√	√	√	√	√	√	√	√	√	√	-	√
Golin 2002	√	√	√	√	√	√	√	√	-	-	√	√	√	√	√	√	√	√	√	√	-	√
Gokarn 2012	√	√	√	√	√	√	√	√	-	-	√	√	√	√	√	√	√	√	-	-	-	-
Gonzalez 2004	√	√	-	√	√	√	√	√	-	-	√	-	√	√	√	√	√	√	-	√	√	-
Gonzalez 2007	-	√	√	√	-	√	√	√	-	-	√	√	√	-	√	√	√	√	-	√	-	-
Gordillo 1999	√	√	√	√	√	-	√	√	-	-	√	√	-	√	√	√	√	√	-	√	-	-
Graham 2007	√	√	√	√	√	√	√	√	-	√	√	√	√	√	√	√	√	√	√	√	-	√
Hanif 2013	√	√	√	√	√	√	√	√	√	-	√	√	√	√	√	√	√	√	√	√	-	√
Hansana 2013	√	√	-	√	√	√	√	√	-	√	√	√	√	√	√	√	√	√	√	√	√	√
Haubrich 1999	√	√	-	√	√	-	√	√	-	-	√	√	√	√	√	√	√	√	√	√	√	-
Heckman 2004	√	√	-	√	√	-	√	√	-	√	√	√	-	√	√	√	√	-	-	-	-	√
Holmes 2007	√	√	√	√√	√	√	√	√	-	-	√	√	√	√	√	√	√	√	√	√	-	-
Holzemer 1999	√	√	√	√	√	√	√	√	√	-	√	√	√	√	√	√	√	√	√	√	√	√
Horne 2007	√	√	√	√	√	√	√	√	-	-	√	√	√	√	√	√	√	√	√	√	√	-
Howard 2002	√	√	√	√	√	√	√	√	-	-	√	√	√	√	√	√	√	√	√	√	√	√
Huang 2013	√	√	√	√	√	√	√	√	-	-	√	√	√	√	√	√	√	√	-	√	-	√
Huyngh 2013	√	√	√	√	√	√	√	√	-	-	√	√	√	√	√	√	√	√	√	-	-	√
Ickovics 2002	√	√	√	√	√	√	√	√	-	-	√	√	√	√	√	√	√	√	√	√	√	-

	1	2	3	4	5	6	7	8	9	10	11	12	13	14	15	16	17	18	19	20	21	22
Ingersoll 2004	√	√	√	√	√	√	√	√	√	-	√	√	√	√	√	√	√	√	√	√	-	√
Ingersoll 2005	√	√	√	√	√	√	√	√	√	-	√	√	√	√	√	√	√	√	√	√	-	√
Johnson 2003	√	√	√	√	√	√	√	√	-	-	√	√	√	√	√	√	-	√	√	√	-	√
Johnson 2005	√	√	√	-	√	√	√	√	-	-	√	√	√	√	√	√	√	√	√	√	-	√
Johnson 2007	√	√	√	√	√	√	√	-	-	-	√	√	√	√	√	√	√	√	√	√	-	-
Johnson 2012	√	√	√	√	√	√	√	√	-	-	√	√	√	√	√	√	√	√	√	√	√	√
Juday 2011	√	√	√	√	√	√	√	√	√	-	√	√	-	√	√	√	√	√	√	√	-	√
Kacanek 2010	√	√	√	√	√	√	√	√	-	-	√	√	√	√	√	√	√	√	√	√	-	√
Kalichman 1999	√	√	√	-	√	-	√	√	-	-	√	√	√	√	√	√	√	√	-	-	-	√
Kalichman 2008	√	√	√	-	√	√	√	√	-	-	√	√	-	√	√	√	√	√	√	√	-	√
Kalichman 2010	√	√	√	-	√	√	√	√	-	-	√	√	√	√	√	√	√	√	√	√	√	-
Kamau 2011	√	√	√	-	√	√	√	√	√	-	-	√	-	√	√	√	√	√	√	-	-	√
Kerr 2012	√	√	√	-	√	√	√	√	-	-	√	√	√	√	√	√	√	√	√	√	-	√
King 2012	√	√	√	√	√	√	√	√	-	-	√	√	√	√	√	√	√	√	√	-	-	√
Kleeberger 2001	√	√	√	-	√	-	√	√	-	-	√	√	√	√	√	√	√	√	-	√	-	√
Kleeberger 2004	√	√	√	√	√	-	-	√	-	-	-	-	√	√	√	√	√	√	-	√	-	√
Kumar 2009	√	√	√	√	√	-	-	√	√	-	√	√	-	-	√	√	-	√	-	-	√	-
Kunutsor 2010	√	√	√	√	√	√	√	√	-	-	√	√	√	√	√	√	√	√	√	√	-	-
Kyser 2011	√	√	√	√	√	√	√	√	-	-	√	√	√	√	√	√	√	√	√	√	-	√
Ladefoged 2012	√	√	√	√	√	√	√	√	√	-	√	√	√	√	√	√	√	√	-	-	-	√
Lazo 2007	√	√	√	√	√	-	√	√	-	-	√	-	√	√	√	-	√	√	√	√	-	√
Leombruni 2009	√	√	√	√	√	-	√	√	-	-	√	√	√	√	√	√	√	√	√	√	-	-
Leserman 2008	√	√	√	√	√	√	√	√	-	-	√	√	√	√	√	√	√	√	√	√	-	√
Li 2010	-	-	√	-	-	√	-	√	-	-	√	√	√	√	√	√	√	√	√	√	-	√
Li 2011	√	√	√	√	√	√	√	√	-	-	√	√	√	√	√	√	√	√	√	√	-	√
Luszczynska 2007	√	√	√	-	√	-	√	√	-	-	√	√	√	-	√	-	-	√	√	√	-	-
Lyimo 2014	-	√	√	√	√	√	√	√	-	-	√	√	√	√	√	√	√	√	√	√	√	√

	1	2	3	4	5	6	7	8	9	10	11	12	13	14	15	16	17	18	19	20	21	22
Lynam 2009	√	√	√	√	√	√	√	√	-	-	√	√	-	√	-	√	-	√	√	√	-	√
Malow 2013	√	√	√	√	√	√	√	√	-	-	√	√	√	√	√	√	√	√	√	√	√	√
Mannheimer 2002	√	√	√	√	-	-	-	-	-	-	√	√	√	√	√	√	√	√	√	√	-	-
Mathews 2002	√	√	√	√	√	√	√	-	-	-	√	√	-	√	√	√	√	√	√	√	-	√
McAllister 2013	√	√	√	√	√	√	√	√	-	-	√	√	√	√	√	√	-	√	√	-	-	√
McDonnell Holstad 2006	√	√	√	√	√	-	√	√	-	√	√	√	-	√	√	√	√	-	-	√	-	√
Mellins 2003	√	√	√	√	√	√	√	√	-	-	√	√	√	√	√	√	√	√	√	√	-	√
Molassiotis 2002	√	√	√	√	√	√	√	√	-	-	√	√	√	√	√	√	√	√	-	√	-	√
Moralejo 2006	√	√	√	√	√	-	√	√	-	√	√	√	-	-	√	√	√	-	-	√	-	-
Mugavero 2009	√	√	√	√	√	√	√	√	-	-	√	√	√	√	√	√	√	√	√	-	√	-
Murphy 2004	√	√	√	-	√	√	√	√	-	-	√	√	√	-	√	√	-	√	-	√	-	√
Murri 2001	√	√	√	√	√	√	-	√	-	-	-	√	√	√	√	√	√	√	-	√	-	-
Nakimuli 2009	√	√	√	√	√	√	√	√	-	-	√	√	√	√	√	√	√	√	√	√	√	-
Nel 2013	√	√	√	√	√	√	√	√	-	-	√	√	√	√	√	√	√	√	√	√	√	-
Nelsen 2013	√	√	√	√	√	√	√	√	-	-	√	√	√	√	√	√	√	√	√	√	√	√
Negash 2013	√	√	√	√	√	√	√	√	-	√	√	√	√	√	√	√	√	√	√	√	√	√
Nieuwkerk 2001	√	√	√	√	√	√	√	√	-	-	√	√	√	√	√	√	√	√	√	√	-	√
Nilsson Schonnesson 2004	-	√	√	-	√	√	√	√	-	√	√	√	-	-	√	√	√	√	√	√	√	-
Nozaki 2011	√	√	√	√	√	√	√	√	-	-	√	√	√	√	√	√	√	√	√	-	-	√
O'Connor 2013	-	√	√	√	√	√	√	√	-	-	√	√	√	√	√	√	√	√	√	√	√	√
Oguyi 2007	√	√	√	√	√	√	√	√	-	-	√	√	√	√	√	√	√	√	√	√	√	√
Oku 2014	√	√	√	√	√	√	√	√	-	√	√	√	√	√	√	√	√	√	√	√	-	√
Parutti 2006	√	√	√	√	√	-	-	√	-	-	-	√	√	√	√	√	√	√	-	√	-	-
Paterson 2000	√	√	√	√	√	√	√	√	-	-	√	√	√	√	√	√	√	√	-	√	-	-
Peltzer 2010	√	√	√	√	√	√	√	√	-	-	√	√	√	√	√	√	√	√	√	√	√	√
Peretti-Watel 2006	√	√	√	√	√	√	√	√	√	-	√	√	√	√	√	√	√	√	√	√	-	√

	1	2	3	4	5	6	7	8	9	10	11	12	13	14	15	16	17	18	19	20	21	22
Perfuro-Yone 2013	√	√	√	√	√	√	√	√	-	-	√	√	√	√	√	√	√	√	√	√	-	√
Pinheiro 2002	√	√	√	√	√	√	√	√	-	-	√	√	√	√	√	√	√	√	-	√	-	-
Plankey 2009	√	√	√	√	√	√	√	√	-	-	√	√	√	-	√	√	√	√	√	√	√	√
Poquette 2012	√	√	-	√	√	√	√	√	-	-	√	√	√	√	√	√	√	√	√	√	√	√
Protopopescu 2009	√	√	√	√	√	-	-	√	-	-	-	-	-	√	√	√	-	√	-	-	√	√
Raboud 2011	√	√	√	√	√	√	√	√	-	√	√	√	√	√	√	√	√	√	√	√	√	√
Ramadhani 2007	√	√	√	√	√	√	√	√	-	-	√	√	√	√	√	√	√	√	√	√	-	√
Rao 2012	√	√	√	√	√	√	√	√	-	√	√	√	√	√	√	√	√	√	√	√	√	√
Remien 2007	√	√	√	√	√	√	√	√	-	√	√	√	√	√	√	√	√	√	√	√	-	-
Reynolds 2002	√	√	√	√	√	√	√	√	√	√	√	√	√	√	√	√	√	√	√	√	√	-
Reynolds 2004	√	√	√	√	√	√	√	√	-	√	√	√	√	-	√	√	√	√	-	√	-	√
Rintamaki 2006	√	√	√	-	√	√	√	√	-	√	√	√	√	√	√	√	√	-	-	-	-	√
Rodrigues 2012	√	√	√	√	√	√	√	√	-	-	√	√	√	√	√	√	√	√	-	-	-	√
Rougemont 2009	√	√	√	√	√	√	√	√	-	-	√	√	√	√	√	√	√	√	√	√	√	√
Safren 2005	√	√	√	√	√	√	√	√	-	-	√	-	√	√	√	√	√	√	-	-	-	-
Sasaki 2012	√	√	√	√	√	√	√	√	-	-	√	√	√	√	√	√	√	√	√	√	√	√
Sayles 2008	√	√	√	√	√	√	√	√	-	√	√	√	√	√	√	√	√	√	√	√	√	√
Schneider 2004	√	√	√	√	√	√	√	√	-	√	√	√	√	√	√	√	√	√	√	√	√	√
Seguy 2007	√	√	√	√	√	√	√	√	-	√	√	√	√	√	√	√	√	√	√	√	-	√
Sellier 2006	√	√	√	√	√	√	-	-	-	-	-	-	√	√	√	√	√	√	-	√	√	-
Servellen van 2002	√	√	√	√	√	√	√	√	-	-	√	√	√	√	√	√	√	-	√	√	√	√
Sithinamsuwan 2008	√	√	√	√	√	√	√	√	-	-	√	√	√	√	√	√	√	√	-	√	-	-
Shah 2007	√	√	√	√	√	√	√	√	-	-	√	√	√	√	√	√	√	√	√	√	√	√
Sherr 2008	√	√	√	√	√	√	√	√	-	-	√	√	√	√	√	√	√	√	√	√	-	√
Shuter 2008	√	√	√	√	√	`-	√	√	-	-	√	√	-	√	√	√	√	√	√	√	√	√
Silva 2009	√	√	√	√	√	√	√	√	-	√	√	√	√	√	√	√	√	√	√	√	-	-
Simoni 2002	-	√	-	√	√	√	√	√	-	-	√	√	-	-	√	√	-	√	-	-	-	√
Simoni 2012	√	√	√	√	√	√	√	√	-	-	√	√	√	√	√	√	√	√	√	√	-	-

	1	2	3	4	5	6	7	8	9	10	11	12	13	14	15	16	17	18	19	20	21	22
Singh 1999	√	√	√	√	√	√	√	√	-	-	√	√	√	√	√	√	√	√	√	√	√	-
Sodergard 2006	√	√	√	√	√	√	√	√	√	-	√	√	√	√	√	√	√	√	√	√	-	√
Spire 2002	√	√	√	√	√	-	√	√	-	√	√	√	√	-	√	√	√	√	√	√	-	√
Stirratt 2006	√	√	√	√	√	√	√	√	-	-	√	√	√	√	√	√	√	√	√	√	-	√
Sullivan 2007	√	√	√	√	√	√	√	√	√	-	√	√	√	√	√	√	√	√	√	√	√	√
Sumari-de Boer 2011	√	√	√	√	√	√	√	√	-	-	√	√	√	√	√	√	√	√	√	√	-	√
Tadios 2006	-	√	√	√	√	√	√	√	-	√	-	√	√	-	√	√	√	√	√	√	√	√
Tedaldi 2012	√	√	√	√	√	√	√	√	-	-	√	√	√	√	√	√	√	√	√	√	√	√
Teixeira 2013	-	√	√	√	√	√	√	√	-	-	√	√	-	√	√	√	√	√	√	-	-	√
Thrasher 2008	√	√	√	√	√	√	√	√	√	-	√	√	√	√	√	√	√	√	√	√	-	√
Tiyou 2010	√	√	√	√	√	√	√	√	-	-	√	√	√	√	√	√	√	√	√	√	-	√
Tran 2013	√	√	-	√	√	√	√	√	-	-	√	√	-	√	√	√	√	√	√	√	√	√
Ubbiali 2008	√	√	-	-	-	-	√	√	-	√	√	√	-	-	-	-	-	√	-	√	√	-
Ukwe 2010	√	√	√	√	√	√	√	√	-	-		-	-	√	-	-	-	-	√	√	-	-
Unge 2010	√	√	√	√	√	√	√	√	-	-	√	√	√	√	√	√	√	√	√	√	-	-
Uuskula 2012	√	√	√	√	√	√	√	√	-	-	√	√	√	√	√	√	√	√	√	√	-	√
Venkatesh 2010	√	√	√	√	√	√	√	√	-	-	√	√	√	√	√	√	√	√	√	√	-	√
Vyavakahar 2007	√	√	√	√	√	√	√	√	-	-	√	√	√	√	√	√	√	√	√	√	√	√
Wagner 2002	√	√	√	√	√	√	√	√	-	-	√	-	√	√	√	√	√	√	-	√	-	√
Wagner 2003	√	√	√	√	√	√	√	√	-	-	√	√	√	√	√	√	√	√	-	√	-	√
Wagner 2011	√	√	√	-	√	√	√	√	-	-	√	√	-	√	√	√	√	√	√	√	√	√
Waite 2008	√	√	√	√	√	√	√	√	-	-	√	√	√	√	√	√	√	√	√	√	√	√
Wamai 2006	√	√	√	√	√	√	-	-	-	-	√	√	√	√	√	√	√	√	-	√	-	√
Wanchu 2007	√	√	√	√	√	√	√	√	-	-	√	√	√	√	√	√	√	√	√	√	-	-
Wang 2007	√	√	-	√	√	√	√	√	-	-	√	√	√	√	√	√	√	√	√	√	√	√
Wasti 2012	√	√	√	√	√	√	√	√	-	-	√	√	√	√	√	√	√	√	√	√	-	-
Watt 2010	√	√	√	√	√	√	√	√	-	-	√	√	√	√	√	√	√	√	√	√	√	√
Weaver 2005	-	√	√	√	-	√	√	√	-	√	√	√	-	-	√	√	√	-	-	√	-	-
Webb 2009	√	√	√	√	√	-	√	√	-	-	√	√	√	√	√	√	√	√	√	√	-	-

	1	2	3	4	5	6	7	8	9	10	11	12	13	14	15	16	17	18	19	20	21	22
Weidle 2006	√	√	-	√	√	√	-	-	-	-	√	√	√	-	√	√	√	-	-	√	√	√
Willard 2006	√	√	√	√	-	-	√	√	-	-	√	√	√	√	√	√	√	√	-	-	-	-
Woods 2008	√	√	√	-	-	-	√	√	-	-	√	√	√	√	√	√	√	-	√	√	-	√
Woods 2009	√	√	√	-	√	√	√	√	-	-	-	-	√	-	√	√	√	√	√	√	-	√
Woodward 2012	√	√	√	√	√	√	√	√	-	-	√	√	√	√	√	√	√	√	√	√	√	√
Yun 2005	√	√	√	√	√	-	√	√	√	√	-	-	√	-	-	-	-	-	-	-	-	-

1)Title/abstract: Introduction 2) Background/rationale 3) Objectives; Methods 4) Study design 5) Setting 6) Inclusion/exclusion criteria 7) Variables 8) Data sources 9) Bias 10) Study size 11) Quantitative variables 12) Statistical; Results 13) Participants 14) Descriptive data 15) Outcome data 16) Main results 17) Other analyses; Discussion 18) Key results 19) Limitations 20) Interpretation 21) Generalizability; Other information 22) Funding

√ = Good description; - = Incomplete/No description;
