# Supplementary figures and images for: Predictors and correlates of adherence to combination antiretroviral therapy (ART) for chronic HIV infection: a meta-analysis
Source: BMC Med. 2014 Aug 21;12:142. doi: 10.1186/s12916-014-0142-1 (PMC4148019; doi:10.1186/s12916-014-0142-1)

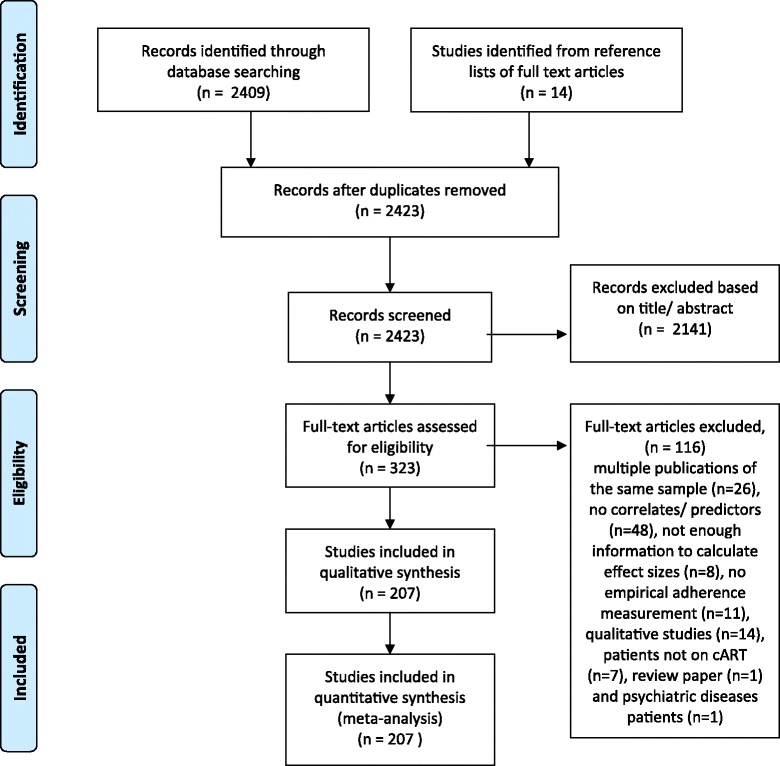

Supplement: Supplementary file 4 — Authors’ original file for figure 1 [file 12916_2014_142_MOESM4_ESM.gif]

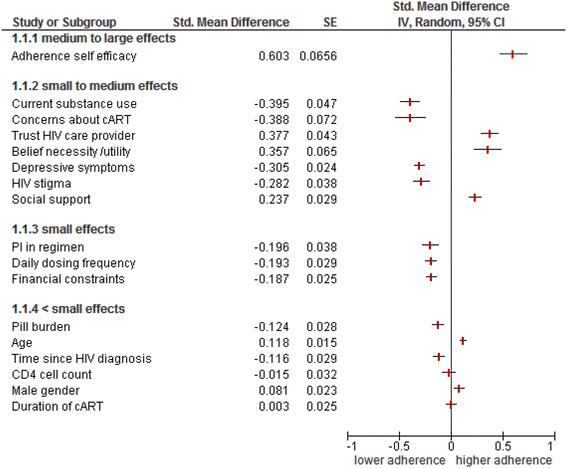

Supplement: Supplementary file 5 — Authors’ original file for figure 2 [file 12916_2014_142_MOESM5_ESM.gif]

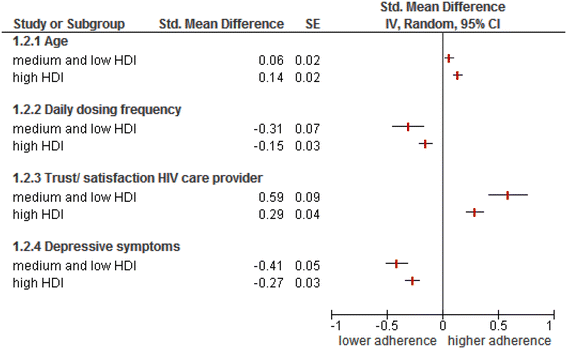

Supplement: Supplementary file 6 — Authors’ original file for figure 3 [file 12916_2014_142_MOESM6_ESM.gif]

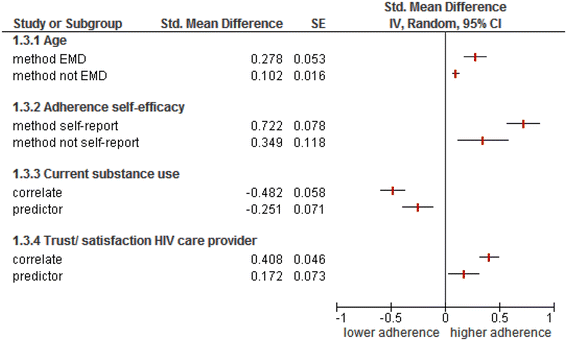

Supplement: Supplementary file 7 — Authors’ original file for figure 4 [file 12916_2014_142_MOESM7_ESM.gif]
